# Circular RNAs increase during vascular cell differentiation and are biomarkers for vascular disease

**DOI:** 10.1093/cvr/cvaf013

**Published:** 2025-02-04

**Authors:** Bernd H Northoff, Andreas Herbst, Catharina Wenk, Lena Weindl, Gabor Gäbel, Andre Brezski, Kathi Zarnack, Alina Küpper, Stefanie Dimmeler, Alessandra Moretti, Karl-Ludwig Laugwitz, Stefan Engelhardt, Lars Maegdefessel, Reinier A Boon, Stefanie Doppler, Martina Dreßen, Harald Lahm, Rüdiger Lange, Markus Krane, Knut Krohn, Alexander Kohlmaier, Lesca M Holdt, Daniel Teupser

**Affiliations:** Institute of Laboratory Medicine, LMU University Hospital, LMU Munich, Marchioninistr. 15, 81377 Munich, Germany; Institute of Laboratory Medicine, LMU University Hospital, LMU Munich, Marchioninistr. 15, 81377 Munich, Germany; Institute of Laboratory Medicine, LMU University Hospital, LMU Munich, Marchioninistr. 15, 81377 Munich, Germany; Institute of Laboratory Medicine, LMU University Hospital, LMU Munich, Marchioninistr. 15, 81377 Munich, Germany; Department of Vascular Medicine, HELIOS Klinikum Krefeld, Krefeld, Germany; Buchmann Institute for Molecular Life Sciences (BMLS), Faculty of Biological Sciences, Goethe University Frankfurt, Frankfurt, Germany; Buchmann Institute for Molecular Life Sciences (BMLS), Faculty of Biological Sciences, Goethe University Frankfurt, Frankfurt, Germany; Institute of Laboratory Medicine, LMU University Hospital, LMU Munich, Marchioninistr. 15, 81377 Munich, Germany; Institute of Cardiovascular Regeneration, Centre of Molecular Medicine, Goethe University, Frankfurt, Germany; Department of Internal Medicine I, Cardiology, Klinikum rechts der Isar, School of Medicine and Health, Technical University of Munich (TUM), Munich, Germany; Department of Internal Medicine I, Cardiology, Klinikum rechts der Isar, School of Medicine and Health, Technical University of Munich (TUM), Munich, Germany; Institute of Pharmacology and Toxicology, Technical University of Munich (TUM), Munich, Germany; Department of Vascular and Endovascular Surgery, Technical University Munich, Munich, Germany; Institute of Cardiovascular Regeneration, Centre of Molecular Medicine, Goethe University, Frankfurt, Germany; Department of Cardiovascular Surgery, German Heart Center Munich, Technical University Munich, Munich, Germany; Institute for Translational Cardiac Surgery (INSURE), German Heart Center Munich, Technical University Munich, Munich, Germany; Department of Cardiovascular Surgery, German Heart Center Munich, Technical University Munich, Munich, Germany; Institute for Translational Cardiac Surgery (INSURE), German Heart Center Munich, Technical University Munich, Munich, Germany; Department of Cardiovascular Surgery, German Heart Center Munich, Technical University Munich, Munich, Germany; Institute for Translational Cardiac Surgery (INSURE), German Heart Center Munich, Technical University Munich, Munich, Germany; Department of Cardiovascular Surgery, German Heart Center Munich, Technical University Munich, Munich, Germany; Institute for Translational Cardiac Surgery (INSURE), German Heart Center Munich, Technical University Munich, Munich, Germany; DZHK (German Center for Cardiovascular Research), Partner Site Munich Heart Alliance, Munich, Germany; Department of Cardiovascular Surgery, German Heart Center Munich, Technical University Munich, Munich, Germany; Institute for Translational Cardiac Surgery (INSURE), German Heart Center Munich, Technical University Munich, Munich, Germany; DZHK (German Center for Cardiovascular Research), Partner Site Munich Heart Alliance, Munich, Germany; Division of Cardiac Surgery, Department of Surgery, Yale School of Medicine, New Haven, CT, USA; Core Unit DNA Technologies, Medical Faculty, University of Leipzig, Leipzig, Germany; Institute of Laboratory Medicine, LMU University Hospital, LMU Munich, Marchioninistr. 15, 81377 Munich, Germany; Institute of Laboratory Medicine, LMU University Hospital, LMU Munich, Marchioninistr. 15, 81377 Munich, Germany; Institute of Laboratory Medicine, LMU University Hospital, LMU Munich, Marchioninistr. 15, 81377 Munich, Germany

**Keywords:** Aneurysm, Atherosclerosis, Biomarker, Circular RNA (circRNA), Differentiation, Endothelial cells (ECs), Induced pluripotent stem cells (iPSCs), Smooth muscle cells (SMCs), Blood, Splicing, Transcription, Transcriptomics, Vascular, Single-cell

## Abstract

**Aims:**

The role of circular RNAs (circRNAs) and their regulation in health and disease are poorly understood. Here, we systematically investigated the temporally resolved transcriptomic expression of circRNAs during differentiation of human induced pluripotent stem cells (iPSCs) into vascular endothelial cells (ECs) and smooth muscle cells (SMCs) and explored their potential as biomarkers for human vascular disease.

**Methods and results:**

Using high-throughput RNA sequencing and a *de novo* circRNA detection pipeline, we quantified the daily levels of 31 369 circRNAs in a 2-week differentiation trajectory from human stem cells to proliferating mesoderm progenitors to quiescent, differentiated EC and SMC. We detected a significant global increase in RNA circularization, with 397 and 214 circRNAs up-regulated greater than two-fold (adjusted *P* < 0.05) in mature EC and SMC, compared with undifferentiated progenitor cells. This global increase in circRNAs was associated with up-regulation of host genes and their promoters and a parallel down-regulation of splicing factors. Underlying this switch, the proliferation-regulating transcription factor MYC decreased as vascular cells matured, and inhibition of MYC led to down-regulation of splicing factors such as SRSF1 and SRSF2 and changes in vascular circRNA levels. Examining the identified circRNAs in arterial tissue samples and in peripheral blood mononuclear cells (PBMCs) from patients, we found that circRNA levels decreased in atherosclerotic disease, in contrast to their increase during iPSC maturation into EC and SMC. Using machine learning, we determined that a set of circRNAs derived from *COL4A1*, *COL4A2*, *HSPG2*, and *YPEL2* discriminated atherosclerotic from healthy tissue with an area under the receiver operating characteristic curve (AUC) of 0.79. circRNAs from *HSPG2* and *YPEL2* in blood PBMC samples detected atherosclerosis with an AUC of 0.73.

**Conclusion:**

Time-resolved transcriptional profiling of linear and circRNA species revealed that circRNAs provide granular molecular information for disease profiling. The identified circRNAs may serve as blood biomarkers for atherosclerotic vascular disease.

## Graphical Abstract

**Graphical Abstract:**
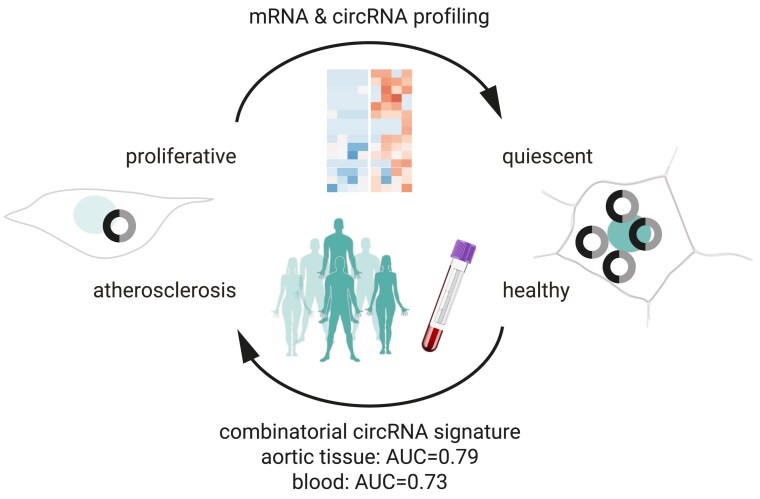

**Time of primary review: 67 days**

## 1. Introduction

High-throughput RNA sequencing (RNA-seq) has revealed that more than 50% of eukaryotic genes express circular RNAs (circRNAs).^1^ circRNAs originate from backsplicing, whereby the cellular spliceosome fuses the end of a downstream exon to the start of an upstream exon. This results in covalently closed, single-stranded circular ribonucleic acids lacking poly(A) tails typical for messenger RNAs (mRNAs).^2–4^ Backsplicing is a rare event because transcription and splicing are organized in ways that favour colinear splicing. Therefore, circRNA steady-state levels represent <10% of host mRNA levels.^2–4^

circRNA biogenesis is considered a biologically controlled event, as cells express cell-type-specific circRNAs at cell-type-specific levels.^5,6^ Generally, RNA-binding proteins can assist backfolding of inverted repeats in flanking introns to promote backsplicing.^7^ Regulators of circRNAs have so far been found mostly from static cell states in cultured cell lines, while the mode of regulation of circRNAs in differentiating systems or during disease onset is less clear.^5–7^

Given their circular nature and the consequent intrinsic molecular stability against cellular RNA degradation complexes, which are largely of 3′–5′ exonucleolytic nature, circRNAs have long half-lives and, due to this property, are currently explored as diagnostic biomarkers for several diseases.^5,6,8^

Most studies on circRNAs as biomarkers have focused on cancer, whereas only a few studies reported circRNA profiles related to cardiovascular diseases.^5^ For example, RNA-seq has identified circRNAs expressed in the myocardium, and differential expression of circRNAs from the *TITIN*, *NPPA*, *CORIN*, *RYR2*, *MYH6*, or *QKI* gene loci has been associated with hypertrophic or dilated cardiomyopathies, heart failure, or arrhythmia.^9^ Even fewer studies focused on circRNAs expressed in vascular tissue such as arteries and veins. A prominent example is *circANRIL*, which is activated at the chromosome 9p21 risk locus and promotes atherosclerotic cardiovascular disease.^10^  *circANRIL* has been identified when studying the molecular consequences of a vascular disease-linked single nucleotide polymorphism on splicing of its primary linear host transcript.^5,9–11^

Apart from the application of circRNA as biomarkers, they might also have merits as molecular features for cell state characterization in basic research. For example, in the vascular system, dedifferentiation in endothelial cell (EC) and smooth muscle cell (SMC) lineages is recently emerging as a factor contributing to the formation of vascular diseases like atherosclerosis and aneurysm.^12,13^ Therefore, circRNA might serve as new molecular tags for such states or other state changes by reporting on transcription-linked splicing decisions not featured by classical transcriptomic analysis.

Here, we systematically determined the previously unknown set of cell-type-specific differentiation-linked vascular circRNAs using a temporally resolved high-throughput RNA-seq approach in a human stem cell differentiation model *in vitro*. The aim was to explore their properties and to test if EC and SMC differentiation-associated circRNAs have a value in describing vascular tissue and cell states for purposes of basic research, and, beyond that, if circRNAs have a diagnostic value in detecting vascular disease.

## 2. Methods

### 2.1 Cell culture

#### 2.1.1 Vascular differentiation protocols from iPSCs

The induced pluripotent stem cell (iPSC) lines ISFi001-A (https://hpscreg.eu/cell-line/ISFi001-A), MRIi003-A (https://hpscreg.eu/cell-line/MRIi003-A), and MRIi001-A (https://hpscreg.eu/cell-line/MRIi001-A) were maintained on hESC-qualified Matrigel-coated dishes (BD Biosciences, Heidelberg, Germany) in mTeSR 1 medium, mTeSR 1 5× supplement, and 1% Pen/Strep (STEMCELL Technologies, Cologne, Germany). For characteristics of iPSC donors and cell type of origin for iPSC generation, see Supplementary material online, *Table S5*. For differentiation, iPSCs were plated on hESC-qualified Matrigel at a density of 32 000 cells/cm^2^ in mTeSR 1 with 10 µM ROCK inhibitor Y-27632 (Santa Cruz Biotechnology, Heidelberg, Germany). After 24 h, the medium was replaced with N2B27 medium [1:1 (*v*/*v*) mixture of DMEM:F12] (Thermo Fisher Scientific, Waltham, MA, USA) containing GlutaMAX and Neurobasal Media supplemented with N2 and B27 (Life Technologies, Thermo Fisher Scientific, Waltham, MA, USA) with 7 µM CHIR-99021 (Tocris, Bio-Techne, Wiesbaden, Germany) and 25 ng/mL BMP4 (R&D Systems, Abingdon, UK) for lateral mesoderm induction.

#### 2.1.2 EC and SMC differentiation trajectory

EC induction medium consisted of StemPro-34 SFM medium (Life Technologies, Thermo Fisher Scientific, Waltham, MA, USA) with GlutaMAX supplemented with 200 ng/mL VEGF-A (R&D Systems, Abingdon, UK) and 2 µM forskolin (Tocris, Bio-Techne, Wiesbaden, Germany). On Day 5 of differentiation, ECs were isolated by MACS using CD144 MicroBeads (Miltenyi Biotec, Bergisch-Gladbach, Germany) and replated on human fibronectin-coated dishes (Sigma-Aldrich, Merck, Darmstadt, Germany) at a density of 26 000 cells/cm^2^ in StemPro-34 SFM with GlutaMAX, supplemented with 50 ng/mL VEGF-A. SMCs were differentiated in N2B27 medium supplemented with 10 ng/mL PDGF-BB (R&D Systems, Abingdon, UK) and 2 ng/mL Activin A (R&D Systems, Abingdon, UK). On Day 5, SMCs were replated at 30 000 cells/cm^2^ on gelatin-coated wells in N2B27 supplemented with 10 ng/mL PDGF-BB.

#### 2.1.3 Functional cell assays

For contact inhibition experiments of ECs, HUVEC-*TERT2* (Evercyte, Vienna, Austria) was seeded at 14 500 cells/cm^2^, and the medium was gradually switched from EGM-2 (Lonza, Basel, Switzerland) on Day 0 to a medium containing 50% M199 (Thermo Fisher Scientific, Waltham, MA, USA)/50% EGM-2 (*v*/*v*). Cells were re-seeded on Day 6 in EGM-2 medium to release cells from contact inhibition. Confluence was determined in 96-well plates with the IncuCyte ZOOM (Sartorius, Göttingen, Germany).

For contact inhibition experiments of SMCs, primary human aortic SMCs (Cat.# ACBRI 716-R, PELOBiotech, Martinsried, Germany) were seeded at 350.000 cells/well in a 6-well dish in SMC cell culture medium (complete classic medium with serum and culture boost, Cat.# 4Z0-500, PELOBiotech). After 48 h, the medium was replaced with DMEM/F12 (Thermo Fisher Scientific) containing 0.2% bovine serum albumin and cells were further cultured for 120 h (‘contact inhibition’). SMCs were released from contact inhibition using trypsin/ethylenediaminetetraacetic acid, reseeded at a density of 150 000 cells/well in a 6-well dish and cultured for a further 30 h in SMC cell culture medium (‘release’). In parallel, SMCs from a low-density culture were seeded in SMC cell culture medium at 150.000 cells/well in a 6-well dish and harvested 30 h later (‘proliferating cells’).

For MYC inhibition, HUVEC-*MYC/ID1/ID2* (CI-huVEC, InSCREENeX, Braunschweig, Germany) was seeded in 6-well plates and grown for 24 h before adding the small-molecule inhibitor 10058-F4 (Merck, Millipore #475956, Darmstadt, Germany). Equal volumes of DMSO served as a control. To account for cell density effects, 200 000 cells were seeded for the 24-h inhibition and 175 000 for the 48-h inhibition.

### 2.2 Human tissue and blood samples

#### 2.2.1 Human AAA samples

Samples for aneurysm profiling were from patients undergoing open abdominal aortic aneurysm (AAA) repair^14^ (project no. EK 316122008 of the Technical University of Dresden). Full-thickness AAA biopsies were obtained from the anterior aneurysm sac and snap frozen in liquid nitrogen. Maximum infrarenal aortic diameter was quantified in patients with AAA from axial computed tomography angiography images or duplex sonography. The AAA cohort comprises 63 men with a mean age of 69.1 years. The median diameter of the aorta was 60.0 mm, with an interquartile range of 21 mm. The cohort was divided into 49 patients who underwent elective repair surgeries and had a mean age of 68.9 years and 14 patients with ruptured AAA who had a mean age of 70.1 years.

#### 2.2.2 Human atherosclerotic aortic tissue samples

Samples from the atherosclerotic aorta or the atherosclerosis-free *aorta mammaria interna* were obtained from individuals of the Munich Study on Mechanisms of Atherosclerosis in Human Tissues MyTi (DHM project no. 5943/13 of the German Heart Center). MyTi is a cross-sectional study of patients undergoing cardiosurgical interventions (samples were provided by the KaBi DHM Biobank, project nos. 5943/13 and 571/16 S). The MyTi tissue cohort includes 48 males and 6 females with an average age of 68.0 and 75.0 years, respectively.

#### 2.2.3 Human PBMCs

Human peripheral blood mononuclear cells (PBMCs) were obtained from independent individuals of the Munich Study on Mechanisms of Atherosclerosis in Human Tissues MyTi (DHM project no. 5943/13 of the German Heart Center). The MyTi PBMC cohort comprises 166 subjects, including 89 coronary artery disease (CAD) patients and 77 control subjects. The CAD patients were 75 males and 14 women, and the control group consisted of 52 men and 25 women.

#### 2.2.4 Ethics approval and consent to participate

Utilization of biopsies from human vascular tissues followed written patient consent to participate, and the cohort studies were approved by the ethics committees of the Hospital of the Technical University of Dresden (EK 316122008), of the Medical Faculty of the Technical University of Munich (Project-Nr. 5943/13), of the German Heart Center (DHM Project-Nr. 5943/13), and of the Technical University Munich (TUM-MRI) (project-Nrs. 2799/10 and 2799/13). All studies were conducted in accordance with the principles of the Declaration of Helsinki.

### 2.3 RNA isolation, reverse transcription, and PCR

#### 2.3.1 RNA extraction of cells and tissue samples

RNA from the snap-frozen aneurysm samples was isolated using the RNeasy Mini Kit (Qiagen, Hilden, Germany). For atherosclerotic tissues, snap-frozen tissues were homogenized with the Precellys 24 Tissue Homogenizer (Bertin Instruments, Montigny-le-Bretonneux, France) and RNA was isolated with TRIzol (Thermo Fisher Scientific, Waltham, MA, USA). Total RNA from human PBMCs and cells was isolated with TRIzol (Thermo Fisher Scientific, Waltham, MA, USA).

#### 2.3.2 RNA quality control

The purity of the isolated RNA was analysed by measuring the absorbance ratio at 260 nm (RNA) and 280 nm (contaminants) using the NanoDrop 2000c spectrophotometer (Thermo Fisher Scientific, Waltham, MA, USA). The integrity of RNA was analysed by measuring the RNA integrity number using the RNA 6000 Nano Kit with the Agilent 2100 Bioanalyzer System (Agilent Technologies, Santa Clara, CA, USA).

#### 2.3.3 Quantitative real-time PCR

Five hundred to 2000 ng total RNA were reverse transcribed with random hexamer primers using 200 U SuperScript II Reverse Transcriptase for 60 min (Thermo Fisher Scientific, Waltham, MA, USA). Qualitative reverse transcriptase–polymerase chain reactions (qRT–PCRs) were performed on a ViiA7 real-time PCR system (Applied Biosystems, Thermo Fisher Scientific, Waltham, MA, USA) using AmpliTaq Gold DNA Polymerase (Life Technologies, Thermo Fisher Scientific, Waltham, MA, USA) and FAM-TAMRA-labelled Taqman probes (Eurofins Genomics, Ebersberg, Germany). For absolute quantification, the qRT-PCR target sequence was cloned into the TOPO II vector, and the concentration of the linearized vector was adjusted for the standard curve. The qRT-PCR results were normalized to housekeeping genes. To validate the circularity of a circRNA, Taqman probes were designed spanning the backsplice junction (BSJ), and outward-facing primers were used that produced a PCR product only in a backsplice event.^15^ Primer and probe sequences are listed in Supplementary material online, *Table S13*.

### 2.4 High-throughput RNA-seq for combined quantification of linear and circRNA

#### 2.4.1 General RNA-seq setup

RNA-seq was performed for parallel quantification of circular and linear RNAs for each time point of *in vitro* differentiation (see Supplementary material online, *Figure S4A*). For circRNA identification, total RNA was pre-treated with RNase R (Lucigen, Biosearch Technologies, LGC, Teddington, UK) (8 U/µg RNA, 120 min, 37°C, 0.5 mM MgCl_2_) and purified with Agencourt RNAClean XP beads (Beckman Coulter, Brea, CA, USA). RNA-seq libraries were prepared using the Ovation Universal RNASeq System Kit (Nugen, Waukesha, WI, USA) with unique dual indices, using 100 ng RNA as input and depleting rRNAs. Libraries were sequenced on a NextSeq550 (Illumina, San Diego, CA, USA), and 20 M paired-end reads were generated on average per sample with a length of 150 nt (see Supplementary material online, *Figure S4C*).

#### 2.4.2 Identification of circRNAs

Sequenced reads of RNase R-treated samples were pre-processed as described in the Supplementary material. Reads were merged and mapped to the primary assembly of the human reference genome GRCh37 using segemehl,^16^ STAR,^17^ and Bowtie 2^18^ software. Splice sites identified by segemehl, and chimeric junctions identified by STAR, were used to identify circRNAs in data of RNase R-treated samples by CIRCexplorer2.^19^ Unmapped reads of RNase R-treated samples identified by Bowtie 2, were realigned to the reference genome (--score-min=C,-15,0 -reorder) and used to identify circular splice sites using find_circ.^20^ The coordinates of backsplicing events of each circRNAs were constrained to be located within one annotated gene of the Gencode database (release 34, GRCh37) and to have a genomic distance between 50 nt and 100 kb. The union of circRNAs identified in all three approaches was used for further analyses.

#### 2.4.3 Quantification of circRNAs and definition of robustly expressed circRNAs

Sequenced reads of RNase R untreated samples were mapped using segemehl with default parameters to a junction library consisting of the human reference genome GRCh37 and contigs representing backsplicing events of circRNAs. For this purpose, 100 nt of the circRNAs at each site of each backsplicing event were merged as contigs. Reads located on the backsplicing event were counted using featureCounts in the paired-end mode (-p) if the sequenced reads covered at least 8 nt (--minOverlap 8) of a 10 nt window located at the backsplicing event, also when reads were mapped multiple (-M). To be considered for quantification, circRNAs with an expression of at least two reads in at least three replicates of at least one time point were considered as robustly expressed.

#### 2.4.4 Linear mRNA expression analysis of annotated genes and transcripts

RNA-seq reads of RNase R-untreated samples were aligned to GRCh37 using HiSAT2 with output for transcript assemblers (--dta). Genes and transcripts were quantified by the StringTie software according to Gencode annotation (release 34, GRCh37) (-e) and reported as Ballgown table files (-B). Expression tables were generated using R packages ballgown and tximport. Hostgenes of circRNAs were identified using R package rtracklayer. Unsupervised hierarchical clustering of cell fate markers was performed for quality control (see Supplementary material online, *Figure S5B*).

### 2.5 Statistics and bioinformatics

#### 2.5.1 Statistical testing

Full details of statistical testing are given in the figure legends. Unless otherwise stated, statistical analyses are two-tailed tests and *P*-values were adjusted for multiple testing using the Benjamini–Hochberg procedure.

#### 2.5.2 Calculation of the ratio of circRNAs to their linear parental mRNAs

A number of reads located on the BSJ and the reads located on the forward splice junctions (FSJs) spanning the 5′ and the 3′ adjacent linear splice junctions were used to calculate the circular-to-linear ratio as CLR = #BSJ/(mean(#FSJ) + 1).

#### 2.5.3 Calculation of cumulative circularization and diversity of circRNA species

To calculate the cumulative circularization, the total number of counts per million (CPM)-normalized BSJs was counted individually for each RNA-seq replicate. The mean cumulative circularization was determined for each differentiation stage. The total number of distinct circRNA species (diversity in terms of different backsplice coordinates involved) was calculated individually for each RNA-seq replicate.

#### 2.5.4 Differential expression analyses of circRNAs, genes, and transcripts

Differential expression changes were analysed using the R package DESeq2.^21^ Based on marker gene expression analysis, the following days of *in vitro* differentiation were selected to analyse differential gene expression: d0 (iPSC state), d02, d03 (mesoderm progenitor-like state), d6, d7, d10, and d12 (vascular EC state or vascular SMC state).

#### 2.5.5 Gene set enrichment analysis

Gene set enrichment analysis was performed with the Molecular Signatures Database from https://www.gsea-msigdb.org/gsea/msigdb using default parameters and DESeq2-normalized linear and circRNA reads to compare days belonging to one of the four phases with the other three phases (iPSC: d0/mesoderm: d2, d3/EC: d6, d7, d10, d12/SMC: d6, d7, d10, d12). Presented data represent a subset of the REACTOME gene sets, or C2 CPG_collapsed gene sets, with an FDR cut-off < 0.2, except for circRNAs of iPSC where no gene set passed the cut-off.

#### 2.5.6 Upstream regulator analyses

Upstream regulators were identified using QIAGEN Ingenuity Pathway Analysis (QIAGEN Inc., https://digitalinsights.qiagen.com/IPA) based on expression changes of annotated target genes.

#### 2.5.7 ROC analyses

For binary classification of human aortic tissue samples into healthy and atherosclerotic samples, the support vector machine (SVM) algorithm^22^ implemented in the mlr3 framework^23^ was applied using circRNA expression levels as features. For this purpose, an SVM model was trained using the expression levels during iPSC > EC differentiation (less differentiated: d0, d2, d3; differentiated: d6, d7, d10, d12). For feature selection, differentially expressed circRNAs with |log_2_FC| > 1 and adjusted *P* < 0.05 between iPSC/mesoderm and mature ECs samples were regarded. These features were ranked in parallel by (i) entropy-based filters (mlr3 filter: ‘information_gain’) and (ii) feature importance filters (mlr3 filter: ‘importance’) using random classification forests. By combining both ranks per feature, the most informative features of the training model (iPSC > EC differentiation) were identified. Afterward, the SVM model was tested and evaluated on independent human arterial samples from individuals of the MyTi cohort (*n* = 54/54) using different combinations of top informative features. For performance measurements, atherosclerotic samples were considered less differentiated, and healthy control samples were considered differentiated according to the iPSC > EC differentiation trajectory. Finally, hyperparameters of the SVM were optimized by tuning costs, gamma, kernel, and degrees. Probabilities of predictions were used for receiver operating characteristic (ROC) analysis and to compute the corresponding area under the ROC curve (AUC).

For the binary classification of human PBMC samples, the *ranger* algorithm implemented in the *mlr3* framework was applied using circRNA expression levels as features. The evaluation of the model was performed using 10-fold cross-validation to avoid overfitting. The AUC was calculated based on the prediction probabilities.

#### 2.5.8 Software and databases

Versions of used software, R packages, and databases are given in Supplementary material online, *Table S14*.

## 3. Results

### 3.1 Identification of novel circRNAs during differentiation of human iPSC to EC and SMC

**Figure 1:**
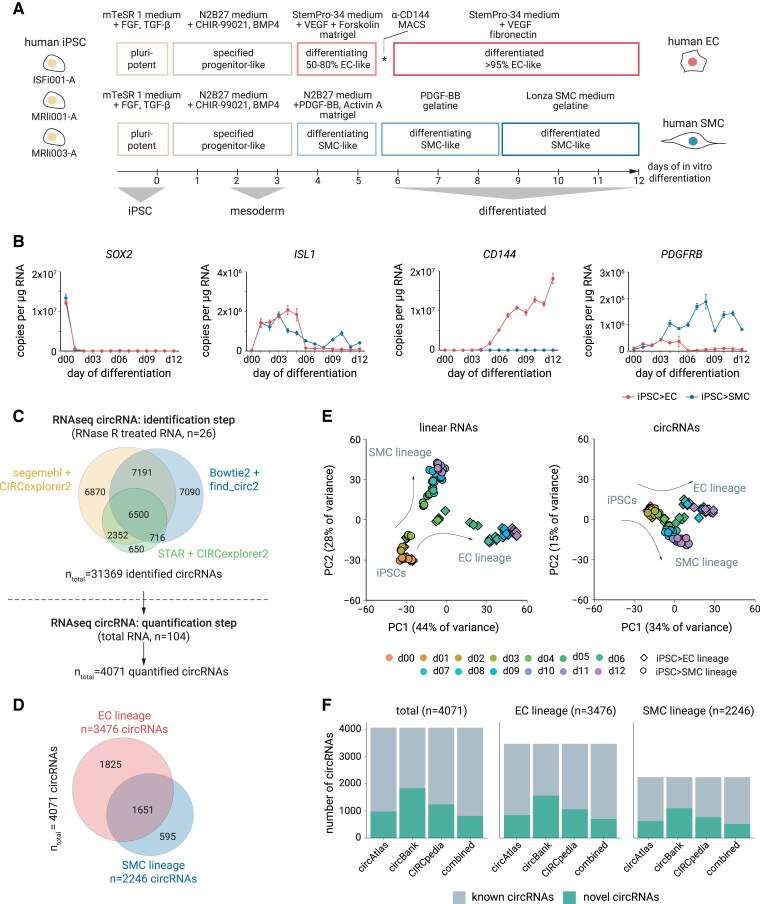
Cell state-specific circRNAs along the differentiation trajectory towards vascular EC and SMC. (*A*) Differentiation scheme of human iPSC to EC and SMC. (*B*) mRNA expression levels of cell fate markers after normalization to 1 μg total RNA and as mean ± SEM. (*C*) Top: circRNA identification pipeline from RNase R-treated total RNA of cells representing the differentiation trajectory [from iPSC and each of the 12 days of the EC (*n* = 13) and SMC (*n* = 13) differentiation schemes]. Cut-offs for identification: 50 bp ≤ BSJ ≤ 100 kb apart from each other and located in the same gene, expression threshold ≥ 2 reads/BSJ. Bottom: quantified circRNAs based on backsplices found in the previous identification step. Expression threshold ≥ 2 BSJ reads in ≥3 replicates of ≥1 day of the differentiation pathway. (*D*) Numbers of quantified circRNAs in the EC and SMC differentiation trajectories (d0–12). (*E*) Principal component (PC) analyses for linear RNAs (left) and circRNAs (right). (*F*) Number of known and novel circRNAs in the three major public circRNA libraries.

Human iPSCs (line ISFi001-A) were differentiated into an intermediate WNT/NODAL-driven mesoderm state, and from there on, in two parallel experimental trajectories, into VEGF-driven EC and PDGF-BB/Activin A-driven SMC, respectively (*Figure 1A*). The differentiated cell stages corresponded to SMC and EC,^24^ and the temporal transitions during differentiation were confirmed by marker^25^ gene expression (*Figure 1B*). At the protein level, FACS analysis (see Supplementary material online, *Figure S1*) and immunofluorescence stainings (see Supplementary material online, *Figure S2*) demonstrated that EC acquired typical EC markers CD31 (PECAM1) and CD144 (CDH5/VE-Cadherin) and did not show SMC markers CD140b (PDGFRB), while SMC acquired SMC markers and did not show CD31 or CD144. Taken together, the two differentiation schemes produced vascular EC or SMC cultures that were eventually >98% homogenous regarding these markers (see Supplementary material online, *Figures S1* and *S2*) and reached confluence at the endpoints (see Supplementary material online, *Figure S3*). We then generated daily profiles of circular and linear RNAs from the iPSC line ISFi001-A for integrated transcriptomic profiling. To this end, we performed paired-end high-throughput RNA-seq in the pluripotent state and at each of the 12 days of differentiation to EC or SMC (see Supplementary material online, *Figure S4*). We first focused on linear mRNAs to determine the specificity of the differentiation scheme. We found that stem cells differentiated to mesoderm-like lateral plate progenitors cells by qRT-PCR (positive for *EOMES*, *HAND1*, *MIXL1*, and *TBXT*) (see Supplementary material online, *Figure S5A*) and by RNA-seq (*WNT3A*, *MESP1*, *NKX2−5*, and *SNAI1*) (see Supplementary material online, *Figure S5B*) and then into maturing EC or SMC. These EC and SMC showed down-regulation of mesodermal markers (see Supplementary material online, *Figure S5A* and *B*). In the EC trajectory, we saw up-regulation of EC adhesion, signalling, and transcription factors DLL4, KDR, NOS3, and VWF (see Supplementary material online, *Figure S5A*) and *CDH5*, *ESM1*, *ERG*, *CLDN5*, and *ENG* (see Supplementary material online, *Figure S5B*). Furthermore, arterial fate markers some stalk-EC and more tip-EC markers and ultimately characteristics of densely packed quiescent phalanx-EC (*DLL4*, *KDR*, among others) were found in EC (see full RNA-seq data-set deposited in GSE210361). These markers were not induced in the SMC differentiation (see Supplementary material online, *Figure S5A* and *B*). At the same time, vascular SMC cultures were positive for typical SMC markers like *ACTA2*, *ACTG2*, *LMOD1*, and *PDGFRB* (see Supplementary material online, *Figure S5A*) or *COL3A1*, *LUM*, *MEIS2*, *BMP5*, *HAND2*, *RSPO2*, and *TBX4* (see Supplementary material online, *Figure S5B*), and these markers were not apparent in the EC differentiation trajectory (see Supplementary material online, *Figure S5A* and *B*). Thus, changes in linear mRNAs separated RNA-seq samples along the time course of differentiation (d1–d12), distinguished EC from SMC (see Supplementary material online, *Figure S5A* and *B*), and showed that we had differentiated cells to molecular EC and SMC phenotypes with markers typical of cultured EC or SMC fate, respectively.

We then identified and quantified circRNAs during differentiation of iPSCs (line ISFi001-A). To increase the number of trusted circRNAs and simultaneously annotate novel circRNAs, we combined three existing non-redundant circRNA identification algorithms. These algorithms differed in how BSJs were detected from the mapping information of multiple-split reads alignment to the genome^26^ (*Figure 1C*). Using pools of RNase R 3′–5′ exonuclease-treated and rRNA-depleted RNA from each day of the differentiation scheme, we identified 31 369 distinct BSJs. Of these, we quantified 4071 robustly expressed circRNAs (*Figure 1D*). Principal component analysis showed that the circRNA expression data contained sufficient molecular information to separate the different cell stages and to separate samples along the time course of differentiation (*Figure 1E*; Supplementary material online, *Figure S6A*). The majority of circRNAs originated from protein-coding genes (see Supplementary material online, *Figure S6B*) and involved annotated exons (see Supplementary material online, *Figure S6C*). We validated the circular nature of the identified circRNAs by resistance to RNase R (see Supplementary material online, *Figure S7*). This approach identified 816 novel circRNAs not previously described in public databases (*Figure 1F*), documenting that combinatorially using different circRNA mappers increases sensitivity in detection and that using an RNase R enrichment step on pooled cells prior to quantification further improves circRNA detectability. Taken together, we established a large and, in part, previously unknown set of thousands of circRNAs, which allowed time-resolved profiling of molecular changes during vascular differentiation.

### 3.2 Temporal profiling reveals phase- and cell-type-specific circRNAs in vascular differentiation

**Figure 2:**
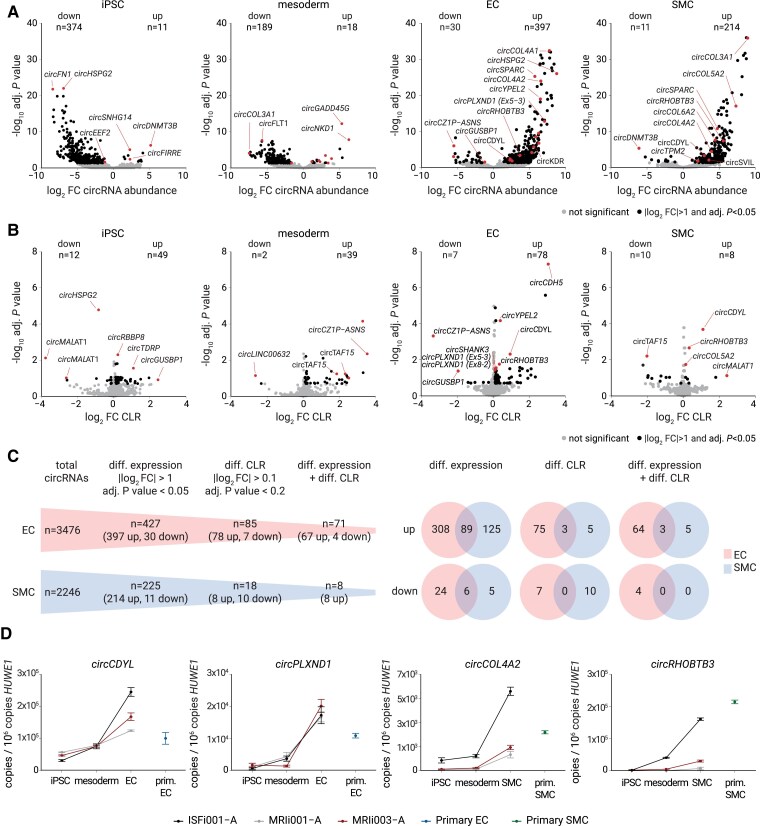
Phase- and cell-type-specific circRNA signatures in differentiating vascular EC and SMC. (*A*) Volcano plots from RNA-seq data highlight circRNAs differentially expressed in each of the four phases of the trajectory of iPSC differentiation to SMC or EC (black dots: significant circRNAs with adjusted *P* < 0.05 and ∣log_2_FC∣ > 1). (*B*) Volcano plots showing the ratio of BSJ reads and cognate FSJs (CLR for each of the four phases in the trajectory of iPSC differentiation to SMC or EC; black dots: significant circRNAs with adjusted *P* < 0.2 and ∣log_2_FC∣ > 0.1). (*C*) Summary of the numbers of identified circRNAs, circRNAs with differential abundance, and circRNAs with differential CLR. (*D*) qRT-PCR validation of expression trajectories for selected EC or SMC circRNAs by independent *in vitro* differentiation of two additional iPSC lines from different donors (iPSC line names: ISFi001-A, MRIi003-A, and MRIi001-A). Expression levels normalized to 10^6^ copies *HUWE1* are reported as mean ± SEM.

We next systematically delineated the identity and level of circRNAs for each of the three major phases of differentiation compared with the other two phases (Phase 1: proliferative pluripotent iPSC; Phase 2: proliferative committed mesoderm progenitor; Phase 3: differentiated vascular EC or SMC). Our analysis revealed that specific circRNA signatures were present in each phase (*Figure 2A* and *B*; Supplementary material online, *Tables S1* and *S2*), which allowed to determine unique circRNAs (see Supplementary material online, *Figure S8A*–*D*). circRNAs were predominantly derived from host genes known to be active in the respective differentiation phase. For example, in iPSC, we found circRNAs from host gene loci encoding the *DNMT3B* DNA methyltransferase or the lncRNA *FIRRE*, two genes known to be expressed in the pluripotent stem cell state. In the mesoderm, we found circRNAs from several loci involved in WNT signalling, such as *LEF1* and *NKD1*. These two genes encode regulators of embryonic mesoderm development, consistent with WNT as the driver of mesoderm in our system. Regarding EC differentiation, we found circRNAs from host genes encoding key signalling and transcription factors with well-established EC biology (*FLT1*, *ESAM*, *ERG*, *TEK*, *TIE1*, *KDR*, *CDH5*, *ETS1*, and *MALAT1*) (see Supplementary material online, *Figure S8*). In the SMC lineage, we found circRNAs from the *CALD1*, *COL1A1*, *CRP1*, and *TPM2* loci (see Supplementary material online, *Figure S8*). Remarkably, when differentiated to the mature vascular fate, we found 13.2 and 19.5 times more up-regulated than down-regulated circRNAs in iPSC-derived EC and SMC, respectively. Thus, 397 and 214 circRNAs were up-regulated greater than two-fold in mature EC and SMC, respectively (adjusted *P* < 0.05) (*Figure 2C*). Of the thousands of expressed circRNAs, a small fraction, 85 circRNAs in EC and 18 circRNAs in SMC, showed some degree of independence from linear host mRNAs, as evidenced by a significant increase in the CLR (*Figure 2C*; Supplementary material online, *Tables S3* and *S4*). These data demonstrate that our profiling approach delineated specific vascular EC and SMC differentiation-linked circRNAs and, in addition, revealed an overarching circRNA up-regulation during differentiation.

To test whether the specific expression and dynamic up-regulation of circRNAs were a generalizable and robust biological trait, we examined several identified circRNAs by qRT-PCR in two additional independent iPSC lines from two different donors (MRIi001-A and MRIi003-A). In contrast to the initially sequenced iPSC line ISFi001-A, these two iPSC lines stemmed from two adult donors, one male and one female, and had been derived from different cell types, from PBMCs or from dermis fibroblasts (see Supplementary material online, *Table S5*). We found by qRT-PCR that the identified vascular EC and SMC circRNAs and their up-regulation were reproducible features of the differentiation trajectory in all three genetically distinct iPSC lines (*Figure 2D*; Supplementary material online, *Figures S9–S11*). In addition, we found that circRNA levels of iPSC-derived vascular cells reached levels similar to those of independently derived primary EC and SMC from human donors (*Figure 2D*; Supplementary material online, *Figures S9–S11*). These data demonstrate the robustness of our differentiation model and circRNA changes and show that circRNAs from the *in vitro* model system represent the adult cultured EC and SMC cell states.

### 3.3 circRNAs are up-regulated during differentiation, increased in cell quiescence, and follow linear RNA expression of host genes

**Figure 3:**
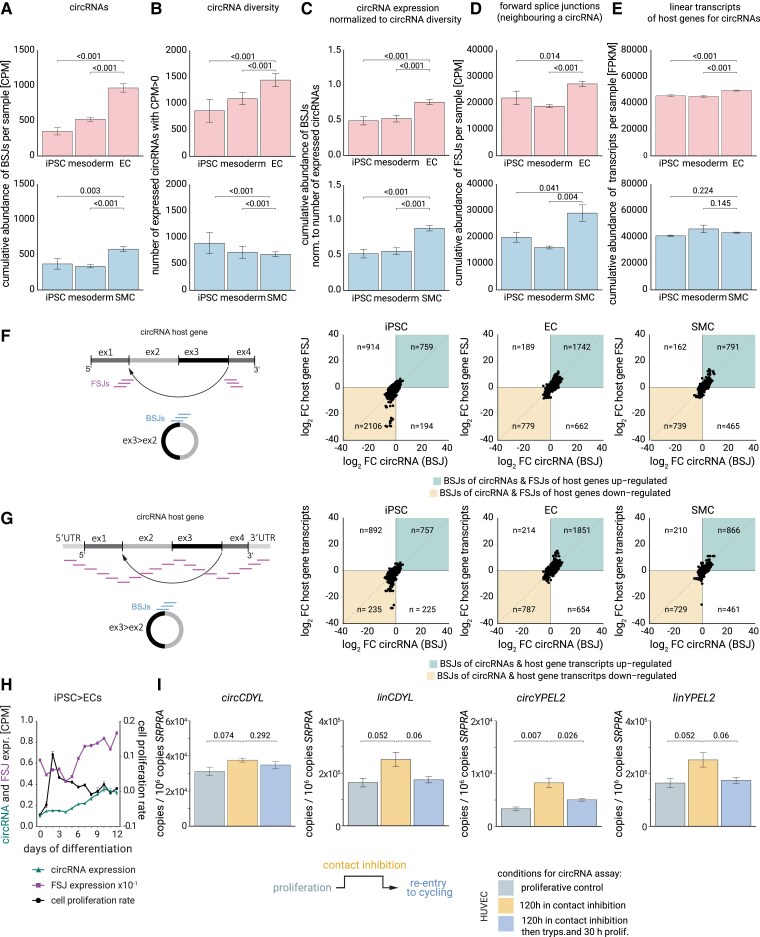
Global up-regulation of circRNAs during vascular differentiation and influence of proliferation rate on circRNAs. (*A*) Cumulative abundance of circRNAs during vascular differentiation as the sum of normalized BSJ counts per phase in EC (top) or SMC (bottom). (*B*) Number of expressed circRNA species (with unique coordinates) per stage (iPSC, mesoderm, EC/SMC). (*C*) circRNA levels from (*A*) after normalization for circRNA diversity from (*B*). (*D*) Sum of normalized FSJ counts per phase from RNA-seq and calculated in analogy to (*A*). (*E*) Sum of normalized counts per phase representing entire linear transcripts from RNA-seq. Statistical testing in *A–E* used the Wald test. (*F*) Log–log plots of state-dependent changes in circRNAs (BSJs) and their corresponding FSJs (up or down). Coherent changes (coloured quadrants) compared with opposing changes (non-coloured quadrants). Numbers denote unique circRNAs in each quadrant. (*G*) Log–log plots of state-dependent changes of circRNAs (BSJs) and their linear host transcripts (up or down). Numbers indicate unique circRNAs in each quadrant. (*H*) Plot with two *y*-axes, showing cumulative abundance of BSJ RNA-seq reads by CPM per day (left *y*-axis) and cumulative abundance of FSJ RNA-seq reads by CPM per day (left *y*-axis) as well as cell proliferation rate along the 12 days of differentiation from iPSC to EC (right *y*-axis). (*I*) qRT-PCRs from HUVEC*-TERT* cell line of exemplary circRNAs and cognate linear host mRNA transcripts, in proliferative conditions (grey), or during 120 h of contact inhibition (yellow), or when contact-inhibited cells were released into proliferation (blue). Data are mean ± SEM of *n* = 3 cell culture replicates measured by qRT-PCR in quadruplicates. *P*-values indicate significance of pairwise comparisons using unpaired two-tailed *t*-tests.

To determine whether the observed up-regulation of hundreds of individual circRNAs during differentiation reflected a confined expression set or a global trend in backsplicing at the transcriptomic scale, we calculated the cumulative abundance of all circRNAs as the sum of all quantified BSJ reads per sample. Summing up the levels for all circRNAs in each sample for each day of differentiation and comparing the differentiated vs. undifferentiated states, we found a significant 2.8-fold and 1.6-fold increase in (transcriptome-wide) backspliced reads in EC and SMC, respectively (*Figure 3A*). Because the number of unique circRNAs with unique backsplice coordinates also increased (*Figure 3B*), we then normalized circRNA abundance to the number of unique circRNAs. After normalization, the net increase in circRNA levels still persisted (*Figure 3C*; Supplementary material online, *Figure S12A* and *B*). We refer to this increase in normalized levels as the ‘global circRNA increase’.

We then asked if this global circRNA increase was due to a continuous process in our stem cell differentiation, such as aging during the 2-week cell culture. Tracking the temporal origin of circRNA up-regulation, we found that only a small fraction of the differentiation-linked circRNAs (14.5% of EC-specific circRNAs and 22.9% of SMC-specific circRNAs) were already robustly expressed in the progenitor cell states (see Supplementary material online, *Figure S12C*). Even when decreasing the detection cut-off to a single read to include weakly expressed circRNAs, substantial up-regulation of circRNA levels and *de novo* production occurred only after the vascular commitment phase. This happened after Day 3 when VEGF or PDGF-BB/Activin A signalling initiated EC or SMC differentiation (see Supplementary material online, *Figure S12D*). These dynamics indicate a non-continuous, contextual up-regulation of circRNA levels and position circRNA up-regulation to the entry of vascular specification.

To test whether the differentiation-linked increase in expression was specific for circRNAs, we quantified the sum of all linear FSJs, defined as local FSJs neighbouring a given BSJ (see methods). We found 1.2- and 1.5-fold more of these FSJs in EC and SMC, respectively, compared with the progenitor phases (*Figure 3D*). The effect for FSJs was less pronounced than the circRNA effect (*Figure 3A–C*). Finally, the sum of RNA-seq reads of the full-length linear transcripts of circRNA host genes in EC and SMC increased only 1.1-fold (*Figure 3E*). Considering the magnitudes of effect sizes, these data suggest that the global differentiation-linked increase in expression was a circRNA-specific feature. Nevertheless, the up-regulation was, to a smaller extent, also related to the expression of primary linear host genes. Corroborating this view from another perspective, we found that most BSJ-FSJ pairs, namely 75% of BSJ-FSJ pairs in EC and 71% in SMC, consistently changed in the same direction (*Figure 3F*). Similarly, 75% of EC circRNAs and 70% of SMC circRNAs changed concordantly in the same direction as their respective linear host transcript (up/up or down/down) (*Figure 3G*). These data reveal that there is a coherent change in all three features—circRNAs, host gene transcripts, and local linear splicing—during vascular differentiation. We list the names of top-regulated circRNAs from our study in *Table 1*.

**Table 1:** Top differentially expressed circRNAs in iPSC-derived EC or SMC

Lineage	circRNA	Differential expression		Circular-to-linear ratio		Host gene	circRNAs per host gene	Annotated circRNA	qRT-PCR
		log_2_FC	Adjusted *P*	log_2_FC	Adjusted *P*				
iPSC > EC	chr1: 183 083 665–183 084 772	3.02	1.87E^−06^	0.24	1.74E^−01^	*LAMC1*	3	Yes	n.d.
iPSC > EC	chr1: 22 213 912–22 214 167	8.07	2.51E^−28^	0.13	1.74E^−01^	*HSPG2*	39	No	n.d.
iPSC > EC	chr2: 111 212 427–111 213 132	4.86	1.41E^−03^	0.18	8.44E^−02^	*AC112229.3*	1	Yes	n.d.
iPSC > EC	chr2: 160 019 793–160 027 316	5.42	3.47E^−04^	0.34	9.15E^−02^	*TANC1*	4	Yes	n.d.
iPSC > EC	chr2: 160 035 066–160 042 410	4.71	7.06E^−03^	0.14	1.95E^−01^	*TANC1*	4	Yes	n.d.
iPSC > EC	chr2: 197 135 916–197 172 809	5.66	5.25E^−08^	0.18	7.40E^−02^	*HECW2*	3	Yes	n.d.
iPSC > EC	chr2: 202 010 100–202 014 558	4.99	1.05E^−03^	0.16	1.78E^−01^	*CFLAR*	2	Yes	n.d.
iPSC > EC	chr2: 36 744 469–36 744 685	5.95	5.10E^−16^	0.12	1.95E^−01^	*CRIM1*	13	Yes	n.d.
iPSC > EC	chr2: 71 753 349–71 766 369	5.48	3.43E^−05^	0.12	8.93E^−02^	*DYSF*	2	Yes	n.d.
iPSC > EC	chr2: 96 500 106–96 500 313	3.88	3.98E^−03^	0.92	3.10E^−02^	*LINC00342*	1	No	n.d.
iPSC > EC	chr3: 121 354 581–121 356 103	5.27	5.18E^−04^	0.28	1.24E^−01^	*HCLS1*	1	Yes	n.d.
iPSC > EC	chr3: 129 302 409–129 308 370	5.17	3.36E^−06^	0.1	3.61E^−02^	*PLXND1*	3	Yes	Yes
iPSC > EC	chr3: 129 304 794–129 305 562	7.07	1.56E^−13^	0.12	3.10E^−02^	*PLXND1*	3	Yes	Yes
iPSC > EC	chr3: 37 544 668–37 559 100	3.51	4.49E^−03^	0.13	1.65E^−01^	*ITGA9*	1	Yes	n.d.
iPSC > EC	chr3: 49 159 116–49 159 518	5.43	2.52E^−04^	0.11	8.89E^−02^	*LAMB2*	1	No	n.d.
iPSC > EC	chr3: 58 131 647–58 135 954	3.45	4.47E^−06^	0.28	8.44E^−02^	*FLNB*	5	Yes	n.d.
iPSC > EC	chr4: 140 599 696–140 625 350	4.27	1.80E^−03^	0.23	6.89E^−02^	*MGST2*	1	Yes	n.d.
iPSC > EC	chr4: 177 632 652–177 650 900	4.94	4.35E^−03^	0.24	1.58E^−01^	*VEGFC*	1	Yes	n.d.
iPSC > EC	chr5: 138 117 611–138 160 488	3.25	1.34E^−03^	0.14	4.86E^−02^	*CTNNA1*	1	No	n.d.
iPSC > EC	chr5: 141 037 931–141 038 046	4.93	4.30E^−03^	0.13	1.24E^−01^	*ARAP3*	2	No	n.d.
iPSC > EC	chr5: 21 491 429–21 497 305	−1.22	3.30E^−02^	−1.95	4.18E^−02^	*GUSBP1*	3	Yes	Yes
iPSC > EC	chr5: 38 971 978–38 982 138	5.2	2.42E^−03^	0.22	1.24E^−01^	*RICTOR*	2	Yes	n.d.
iPSC > EC	chr5: 55 259 179–55 264 224	4.86	4.93E^−05^	0.28	1.05E^−01^	*IL6ST*	1	Yes	n.d.
iPSC > EC	chr5: 95 091 099–95 099 324	3.68	4.47E^−04^	0.34	1.74E^−02^	*RHOBTB3*	1	Yes	Yes
iPSC > EC	chr6: 144 750 716–144 757 165	4.88	1.50E^−03^	0.11	1.19E^−01^	*UTRN*	12	No	n.d.
iPSC > EC	chr6: 151 130 240–151 130 870	4.61	9.19E^−03^	0.16	1.74E^−01^	*PLEKHG1*	1	Yes	n.d.
iPSC > EC	chr6: 166 829 529–166 829 653	3.97	7.21E^−04^	1.49	7.38E^−02^	*RPS6KA2*	3	No	n.d.
iPSC > EC	chr6: 166 829 529–166 829 724	4.18	2.95E^−06^	1.9	4.03E^−02^	*RPS6KA2*	3	No	n.d.
iPSC > EC	chr6: 166 843 940–166 845 978	6.24	1.83E^−05^	0.36	1.95E^−01^	*RPS6KA2*	3	Yes	n.d.
iPSC > EC	chr6: 168 307 850–168 363 247	2.17	1.12E^−02^	0.14	1.23E^−01^	*AFDN*	4	No	n.d.
iPSC > EC	chr6: 4 891 946–4 892 613	2.28	8.86E^−03^	0.94	4.83E^−03^	*CDYL*	1	Yes	Yes
iPSC > EC	chr7: 73 800 850–73 803 589	4.91	7.45E^−04^	0.21	1.83E^−01^	*CLIP2*	7	Yes	n.d.
iPSC > EC	chr7: 97 557 393–97 558 089	−5.5	1.05E^−06^	−3.33	4.75E^−04^	*AC079781.5*	1	Yes	Yes
iPSC > EC	chr9: 132 662 243–132 662 826	4.9	2.07E^−04^	0.21	1.03E^−01^	*FNBP1*	2	Yes	n.d.
iPSC > EC	chr10: 111 876 016–111 882 050	4.52	1.66E^−05^	0.1	1.10E^−01^	*ADD3*	4	Yes	n.d.
iPSC > EC	chr10: 15 170 348–15 183 556	4.48	7.17E^−03^	0.27	1.74E^−01^	*NMT2*	2	Yes	n.d.
iPSC > EC	chr10: 15 290 637–15 296 878	4.53	5.61E^−03^	0.19	1.65E^−01^	*FAM171A1*	2	Yes	n.d.
iPSC > EC	chr10: 15 317 853–15 326 104	3.79	1.35E^−03^	0.16	1.33E^−01^	*FAM171A1*	2	Yes	n.d.
iPSC > EC	chr10: 17 271 339–17 271 531	5.05	5.69E^−05^	1.61	3.10E^−02^	*VIM-AS1*	2	Yes	n.d.
iPSC > EC	chr10: 17 271 733–17 271 937	4.13	1.49E^−02^	1.32	1.73E^−01^	*VIM*	3	Yes	n.d.
iPSC > EC	chr11: 12 495 291–12 495 530	5.56	1.52E^−08^	0.2	7.38E^−02^	*PARVA*	1	Yes	n.d.
iPSC > EC	chr11: 62 292 887–62 293 109	3.79	7.28E^−03^	0.92	1.91E^−01^	*AHNAK*	9	No	n.d.
iPSC > EC	chr11: 65 267 115–65 267 243	2.34	2.20E^−02^	2.09	7.00E^−02^	*MALAT1*	27	No	n.d.
iPSC > EC	chr11: 65 267 869–65 268 112	2.94	1.37E^−03^	2.1	3.15E^−02^	*MALAT1*	27	Yes	n.d.
iPSC > EC	chr11: 65 267 905–65 268 121	3.67	4.95E^−03^	1.37	1.83E^−01^	*MALAT1*	27	Yes	n.d.
iPSC > EC	chr11: 65 267 946–65 268 121	1.98	4.43E^−02^	1.92	1.05E^−01^	*MALAT1*	27	No	n.d.
iPSC > EC	chr11: 65 267 972–65 268 121	5.08	2.64E^−04^	1.47	2.66E^−02^	*MALAT1*	27	No	n.d.
iPSC > EC	chr11: 65 267 990–65 268 121	6.11	1.30E^−15^	2.89	2.63E^−06^	*MALAT1*	27	Yes	n.d.
iPSC > EC	chr11: 70 265 851–70 267 686	2.21	3.72E^−04^	0.17	4.03E^−02^	*CTTN*	10	Yes	n.d.
iPSC > EC	chr12: 133 365 612–133 398 897	4.63	2.48E^−02^	0.15	1.98E^−01^	*GOLGA3*	1	Yes	n.d.
iPSC > EC	chr12: 978 86 238–97 954 825	−4.66	3.70E^−02^	−1.02	1.78E^−01^	*RMST*	1	Yes	n.d.
iPSC > EC	chr13: 110 822 055–110 823 079	7.91	1.08E^−32^	0.14	6.72E^−05^	*COL4A1*	23	No	n.d.
iPSC > EC	chr13: 110 822 893–110 823 034	7.75	4.21E^−33^	0.11	1.30E^−05^	*COL4A1*	23	No	n.d.
iPSC > EC	chr13: 111 857 635–111 862 349	5.57	2.10E^−05^	0.14	7.07E^−02^	*ARHGEF7*	2	Yes	n.d.
iPSC > EC	chr13: 72 131 155–72 147 144	4.3	1.98E^−02^	0.17	1.54E^−01^	*DACH1*	2	Yes	n.d.
iPSC > EC	chr13: 99 481 560–99 498 245	4.73	1.29E^−03^	0.14	1.43E^−01^	*DOCK9*	3	Yes	n.d.
iPSC > EC	chr15: 25 304 301–25 307 235	−2.26	5.15E^−04^	−0.55	1.77E^−01^	*SNHG14*	12	Yes	n.d.
iPSC > EC	chr15: 86 207 793–86 213 061	4.93	1.20E^−02^	0.11	1.74E^−01^	*AKAP13*	5	Yes	n.d.
iPSC > EC	chr16: 47 531 309–47 537 001	3.19	5.58E^−03^	0.16	1.95E^−01^	*PHKB*	1	Yes	n.d.
iPSC > EC	chr16: 66 437 466–66 437 690	6.49	7.30E^−19^	3.05	4.94E^−08^	*CDH5*	1	No	n.d.
iPSC > EC	chr16: 70 176 136–70 177 561	4.71	2.22E^−02^	0.23	1.91E^−01^	*PDPR*	1	Yes	n.d.
iPSC > EC	chr17: 57 430 575–57 430 887	6.5	1.07E^−19^	0.39	6.72E^−05^	*YPEL2*	1	Yes	Yes
iPSC > EC	chr18: 9 525 717–9 525 849	3.58	5.60E^−03^	0.28	1.27E^−01^	*RALBP1*	1	Yes	n.d.
iPSC > EC	chr20: 17 640 309–17 640 459	2.6	2.21E^−02^	0.85	1.95E^−01^	*RRBP1*	8	No	n.d.
iPSC > EC	chr22: 36 690 152–36 690 278	4.83	9.91E^−04^	1.46	4.03E^−02^	*MYH9*	9	No	n.d.
iPSC > EC	chr22: 51 153 344–51 160 865	6.29	1.98E^−07^	0.16	2.78E^−02^	*SHANK3*	4	Yes	Yes
iPSC > EC	chrX: 139 865 419–139 865 879	3.58	2.65E^−02^	0.83	1.95E^−01^	*LINC00632*	29	No	n.d.
iPSC > EC	chrX: 139 865 636–139 865 879	3.58	9.24E^−03^	0.93	1.83E^−01^	*LINC00632*	29	No	n.d.
iPSC > EC	chrX: 139 865 879–139 866 225	3.4	3.33E^−03^	0.88	1.05E^−01^	*LINC00632*	29	No	n.d.
iPSC > EC	chrX: 139 865 879–139 866 261	3.12	2.25E^−04^	1.01	8.93E^−02^	*LINC00632*	29	No	n.d.
iPSC > EC	chrX: 147 743 428–147 744 289	4.22	1.80E^−02^	0.61	1.95E^−01^	*AFF2*	1	Yes	n.d.
iPSC > SMC	chr2: 189 921 698–189 922 153	7.33	9.79E^−18^	0.13	1.80E^−02^	*COL5A2*	2	No	Yes
iPSC > SMC	chr3: 145 838 898–145 842 016	4.96	8.69E^−07^	0.13	9.31E^−02^	*PLOD2*	2	Yes	n.d.
iPSC > SMC	chr5: 95 091 099–95 099 324	5.58	4.53E^−08^	0.32	2.17E^−03^	*RHOBTB3*	1	Yes	Yes
iPSC > SMC	chr6: 160 819 010–160 831 878	6.17	6.23E^−06^	0.31	1.94E^−01^	*SLC22A3*	2	Yes	n.d.
iPSC > SMC	chr6: 4 891 946–4 892 613	2.93	5.57E^−04^	1.09	2.04E^−04^	*CDYL*	1	Yes	Yes
iPSC > SMC	chr8: 38 947 565–38 948 865	5.74	4.07E^−07^	0.23	1.16E^−01^	*ADAM9*	2	Yes	n.d.
iPSC > SMC	chr11: 65 267 115–65 267 243	2.91	2.51E^−03^	2.41	7.49E^−02^	*MALAT1*	28	No	n.d.
iPSC > SMC	chr17: 48 265 907–48 266 844	5.36	9.84E^−08^	1.8	9.31E^−02^	*COL1A1*	10	No	n.d.

EC- or SMC-fate associated circRNAs from RNA-seq profiling with top changes in expression (|log_2_FC| > 1 and adjusted *P* < 0.05), and in CLR (|log_2_FC| > 0.1, *P* < 0.05 and adjusted *P* < 0.2).

n.d., not determined.

A current model to explain global circRNA changes suggests that circRNAs passively decrease when cells divide more rapidly, such as in cancer, because of more rapid macromolecular dilution of the contents of one mother cell into the two daughter cells.^27^ In a similar way, circRNA levels are thought to increase when cells stop dividing, such as in neuronal differentiation.^28^ In our experimental model, cell proliferation indeed decreased during differentiation from Day 2 onwards, coinciding with an increase of global backsplicing and neighbouring forward splicing from Day 4 onwards (*Figure 3H*). In the iPSC differentiation model, differentiation is inherently linked with cell cycle exit. In order to test the macromolecular dilution model functionally, we resorted to adult EC and seeded HUVEC at high density with the aim to induce a contact inhibition-mediated arrest in cell proliferation without grossly affecting EC cell fate (*Figure 3I*; Supplementary material online, *Figure S13A*–*C*). In this way, we arrested EC proliferation for 5 days, while four to five cell divisions could have occurred in the non-inhibited control. This arrest increased circRNA levels (*Figure 3I*; Supplementary material online, *Figure S13D*, yellow bars). However, we found that whenever a circRNA increased, its host mRNA also increased (*Figure 3I*; Supplementary material online, *Figure S13D*, yellow bars). Normalizing circRNA by host gene linear RNA abundance abolished or, at least, reduced this effect for most tested circRNA loci (see Supplementary material online, *Figure S13E*). Conversely, a release from contact inhibition into a normal proliferative state caused a trend of decrease for circRNAs and/or linear FSJs compared with the contact-inhibited state for several tested genes (*Figure 3I*; Supplementary material online, *Figure S13D*, blue bars). Speaking for coherent changes of host mRNA and circRNA, the CLR changed only mildly throughout conditions and did not strongly increase by contact inhibition (see Supplementary material online, *Figure S13E*). Thus, multiple lines of experimental evidence suggest that circRNA abundance is responsive to the proliferation rate, is predetermined by linear host RNA level, and is not uniquely due to passive concentration/dilution. Specifically, these data disentangle the influence of proliferation on circRNA levels because overall differentiation markers were not affected in this experiment.

### 3.4 MYC regulates vascular splice factors and differentiation-associated vascular circRNAs

**Figure 4:**
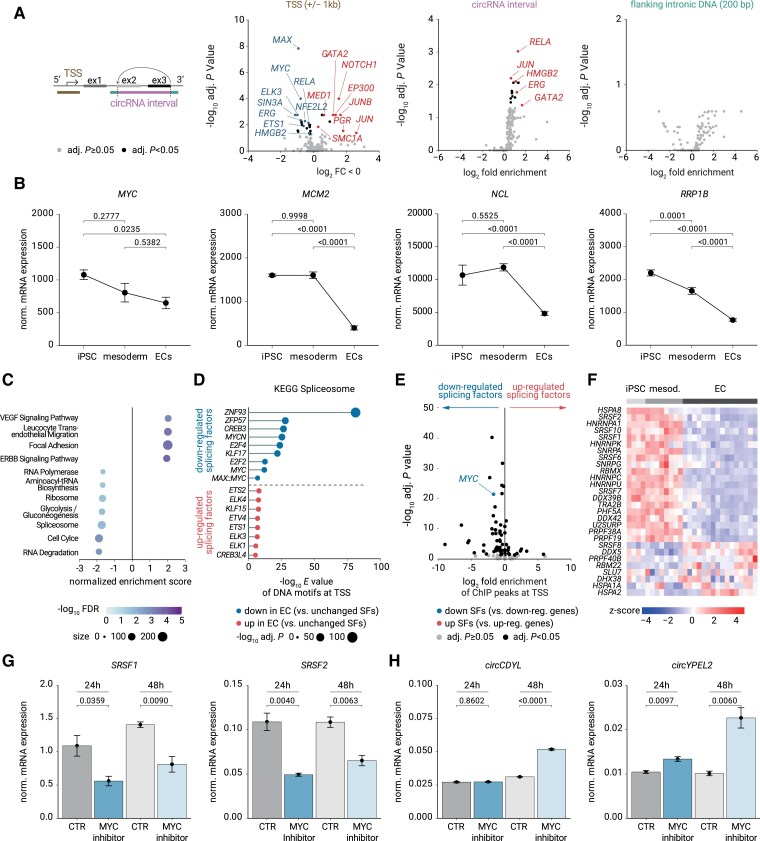
MYC regulates vascular SFs and circRNAs. (*A*) Scheme for ChIP peak enrichment analysis: genomic regions of circRNA host genes were inspected that increased in EC during iPSC > EC differentiation (log_2_FC > 1, adjusted *P* < 0.05) compared with host loci for unchanged circRNAs. Transcription start sites (TSS), or 200 bp flanking the BSJs, or backspliced exons were explored by chip-atlas from NCBI-SRA endothelial ChIP-seq data sets. (*B*) RNA-seq-based mRNA expression of *MYC* and E-box-containing MYC target genes as mean ± SEM. *P*-values from analysis of variance with Tukey's *post hoc* test. (*C*) Results of gene set enrichment analysis (GSEA) of linear mRNAs during RNA-seq of iPSC > EC differentiation trajectory using KEGG annotated human gene sets. (*D*) Local DNA motif enrichment from CentriMO analysis within ±2 kb around the TSS of SFs increasing in EC (red) or decreasing in EC (red). SFs were from *C* and compared with TSS of unchanged SFs, respectively (adjusted *P* > 0.05). Top results are shown (*E*-value cut-off < 10e^−5^ and hit ranking by Fisher *E*-value). (*E*) Global enrichment chip-atlas analysis of MYC protein association with genomic regions ±2 kb around the TSS of SFs (from *D*) to the TSS of all expressed genes. (*F*) Heatmap showing mRNA levels during *in vitro* iPSC > EC differentiation for the top 20 down-regulated and all (8) up-regulated SFs that share MYC and E2F motifs from *D*. Note members of the SRSF family. (*G*) qRT-PCR for changes in *SRSF1* and *SRSF2* mRNA in immortalized HUVEC cell line (HUVEC*-MYC/ID1/ID2*) after MYC inhibition with 30 μM MYC inhibitor 10058-F4. (*H*) Effect of MYC inhibition with 30 μM 10058-F4 on selected circRNAs in HUVEC*-MYC/ID1/ID2*. Data of *n* = 3 cell culture replicates measured by qRT-PCR in quadruplicates are given as mean ± SEM. *P*-values from ANOVA with Tukey's *post hoc* test (*G*, *H*). SF, splice factor.

To determine how differentiation-associated circRNA host genes were regulated, potentially in response to proliferation rate, we examined genomic and epigenomic regulatory features at their loci. To this end, we first performed ChIP peak calling from published ChIP-seq data sets of vascular ECs in chip-atlas.^29^ We compared the 212 host genes representing the 397 induced EC-circRNAs with the 1831 host genes encoding the 3079 unchanged circRNAs (*Figure 4A*). Within ±1 kb around the transcription start sites of genes encoding up-regulated vascular circRNAs and in the circRNA interval but not in the introns flanking the region encoding the circRNAs, we found a disproportionate enrichment of binding by well-known EC morphogenesis- and fate-specifying^30^ transcription factors GATA2, JUN, ERG, and RELA (*Figure 4A*). Conversely, DNA binding by the cell proliferation and growth-stimulating MYC and MAX proteins^31^ was the top depleted feature (*Figure 4A*).

Concerning up-regulated factors from ChIP at circRNA loci, we found that mRNA levels of these potential upstream regulators increased upon differentiation (see Supplementary material online, *Figure S14A*). In addition, specific epigenetic coactivators, such as histone acetyltransferase and mediator complexes (EP300, MED1), cohesin subunits (SMC1A) (*Figure 4A*), and the corresponding histone modification H3K27 acetylation were enriched at circularizing exons (see Supplementary material online, *Figure S14B*). This indicates that host genes for EC-enriched circRNAs are molecularly distinguishable by preferential association with EC cell fate-determining transcription factors, suggesting a new model of circRNA biogenesis. Another novel observation was that epigenetic processes related to chromatin topology and histone modifications were associated with backsplicing.

Concerning down-regulated factors from ChIP at circRNA loci, we focused on MYC because timely down-regulation of MYC, which controls many transcription-coupled processes in proliferation control,^32^ is known to contribute to arterialization *in vivo*^33^ and changes splice factor expression^34,35^ and splice site fidelity.^36^ Thus, we began to take into consideration a potential role of MYC as circRNA regulator not only at DNA-level but also at RNA and splicing levels. For that, first, we tested the correlation of expression between different circRNAs and compared this with the correlation of expression of circRNAs and their linear host transcripts. Thereby, we found that circRNAs were indeed also regulated at the RNA level (see Supplementary material online, *Figure S14C*). Corroborating our findings on MYC in ChIP analyses, ingenuity pathway analysis of our RNA-seq data identified MYC as a strongly inhibited upstream regulator during EC differentiation (activation *z*-score = −8.98, *P*-value of overlap = 1.01 × 10^−27^). Thereby, expression levels of 282 of 490 known target genes were consistent with an inhibition of MYC. Consistently, we saw a decline in mRNA levels of *MYC* (log_2_FC = −0.5), its family member *MYCN* (log_2_FC = −5.5), and MYC target genes. These targets were involved in features supporting cell proliferation and growth (*MCM2*), ranging from rRNA biogenesis (*NCL*) over ribosomal biogenesis (*RPP/RPL* factors) to protein translation (*EIF4A1*) (*Figure 4B*; Supplementary material online, *Figure S14D*). This decrease coincided with significant increases of cell quiescence stimulators such as *FOXO1*^37^ (log_2_FC = 2.1, activation *z*-score = 1.1) and *ZEB1*^38^ (log_2_FC = 6.2, activation *z*-score = 2.7), an antiproliferative increases of cyclin-dependent kinase inhibitors (*p21/CDKN1A* (log_2_FC = 2.5, activation *z*-score = 1.2), *p27/CDKN1B* (log_2_FC = 1.1, activation *z*-score = 1.6), and *P16-INK4A/CDKN2B* (log_2_FC = 3.0, activation *z*-score = 0.7). At the same time, a decrease of markers for active cell cycling, such as *PCNA* or mitotic *Cyclin B*, was seen, consistent with the entry of EC into a cell cycle- and growth-related quiescence during differentiation (see Supplementary material online, *Figure S14E*). Extending these results, gene set enrichment analysis (GSEA) showed a broad decline in even broader proliferation-related terms in EC differentiation, ranging from cell cycle over ribosome biogenesis to biosynthetic terms such as tRNA biosynthesis and metabolic terms like glycolysis (*Figure 4C*). Of note, the ‘spliceosome’ pathway also decreased in this context of biosynthetic quiescence (*Figure 4C*). We found that the transcription start sites for the affected down-regulated splice factors were enriched in DNA-binding motifs of the central cell cycle transcription factor E2F family (E2F2/E2F4, *E* < 4.0 × 10^−13^) and the larger growth-regulatory MYC family (MYC/MYCN/MYC:MAX, *E* < 1.5 × 10^−7^) compared with promoters of unregulated splice factor loci (*Figure 4D*; Supplementary material online, *Table S6*). ChIP data from ECs at promoter regions corroborated that promoters of down-regulated splicing factors were more strongly associated with MYC than other down-regulated genes in the genome (*Figure 4E*). Other physical features, such as the distance between splice sites or exon size (see Supplementary material online, Figure  *S15*) and microRNA-binding sites (see Supplementary material online, Figure  *S16*), did not substantially distinguish between EC and SMC circRNA host loci. In addition, we found that splice factors that decreased during EC differentiation shared E2F and MYC binding to promoters (see Supplementary material online, *Figure S17A*). These included several members of the serine–arginine-rich splicing factor family (SRSF1, 2, 6, 7, and 10) (see Supplementary material online, *Figure S17B*). Conversely, induced splicing factors exhibited motifs for EC transcription factors and several E2F motifs (*Figure 4D*; Supplementary material online, *Figure S17C* and *D*). These data indicate that both down- and up-regulation of many splice factor mRNAs occurred during vascular differentiation (*Figure 4F*) and that the relevant splice factor genes may be regulated by the proliferative context of cells via E2F and MYC.

To functionally test whether the proliferation context affected vascular splice factors and if it also affected circRNAs, we applied low doses of the MYC::MAX dimerization inhibitor 10058-F4,^39^ which lowers the activity and protein levels of MYC so that low MYC activity becomes limiting for cell proliferation.^39^ To avoid influences from a dynamic differentiation process (as in the iPSC model), we blocked MYC activity in an immortalized, proliferative EC cell line that had robust MYC levels and a validated EC differentiation state.^40^ We found that MYC inhibition affected mRNA expression of splice factors and caused, for example, a decrease of *SRSF1* and *SRSF2* mRNA (*Figure 4G*). Of note, MYC inhibition also impacted backsplicing in selective ways and, for example, led to increased levels of *circYPEL2* and *circCDYL* (*Figure 4H*). These functional data reveal that levels of splicing factors and vascular circRNAs are molecularly determined by MYC. Because MYC levels are limiting for cell proliferation,^31^ we conclude that the levels of vascular differentiation state-associated circRNAs are coupled to the cellular proliferation state.

Next, we asked if circRNA regulation by proliferation was a more general feature and also valid in other cardiovascular cell types. To test this model in relation to SMC, we analysed our vascular circRNAs in total RNA from a recently published cohort of primary SMCs from 151 genetically diverse donors.^41,42^ We found that proliferative primary SMC had significantly lower levels of circ*HSPG2* (adjusted *P* = 0.035) and *circYPEL2* (adjusted *P* < 0.001) and a trend towards down-regulation of circRNAs for *COL4A1* and *COL4A2* compared with their serum-starved quiescent SMC cell state (see Supplementary material online, *Figure S18A* and *Table S7*). The effect sizes were relatively small, even for *circHSGP2* and *circYPEL2*, in this specific model of serum starvation/re-addition (log_2_FC = 0.38 and 0.47, respectively) (see Supplementary material online, *Figure S18A* and *Table S7*). Therefore, we tested changes in circRNA abundance in a similar, but distinct experimental quiescence model for SMC: we found that physical contact inhibition between primary SMC for 5 days led to significant up-regulation with larger effect sizes for all of the four circRNAs and their linear host mRNAs (*COL4A1*, *COL4A2*, *HSPG2*, and *YPEL2*) (see Supplementary material online, *Figure S18B*, yellow bars). In contrast, when these contact-inhibited SMC were released from each other and seeded again at low density, which allowed proliferation to resume, circRNA and host mRNA levels dropped robustly and significantly, supporting that density-dependent proliferative cues were at least one factor contributing to their regulation (see Supplementary material online, *Figure S18B*, blue bars). Additionally, to stay within the cardiovascular system, we also focused on cardiomyocytes, which execute a proficient cell cycle exit during differentiation.^43^ Indeed, analysing circRNAs in a published data set,^44^ we found more significantly up- than significantly down-regulated circRNAs (adjusted *P* < 0.05: *n* = 34 up, *n* = 4 down) after 14 days of cardiomyocyte differentiation from iPSC, reminiscent of global circRNA induction during vascular EC/SMC differentiation (see Supplementary material online, *Table S8*). Moreover, although different genes were induced in cardiomyocytes compared with EC/SMC, circRNAs from *YPEL2*, *COL4A1*, *COL4A2*, and *HSPG2* also showed a trend towards induction in cardiomyocytes (see Supplementary material online, *Figure S18C*). Also, the respective linear host mRNAs of these circRNAs were induced, corroborating our earlier findings in EC and SMC (see Supplementary material online, *Figure S18C*). Together, these data on MYC inhibition, serum starvation, contact inhibition, and differentiation-associated cell cycle exit suggest the existence of a set of circRNAs and their linear host mRNAs that molecularly respond to changes in cell proliferation rate more generically in cardiovascular cells.

### 3.5 circRNAs that increase in vascular differentiation are down-regulated during atherosclerosis and serve as biomarkers for vascular disease

Next, we investigated whether circRNAs identified during differentiation of iPSC to EC and SMC *in vitro* were also present in human tissues and may be changing levels in the course of vascular disease *in vivo*.

**Figure 5:**
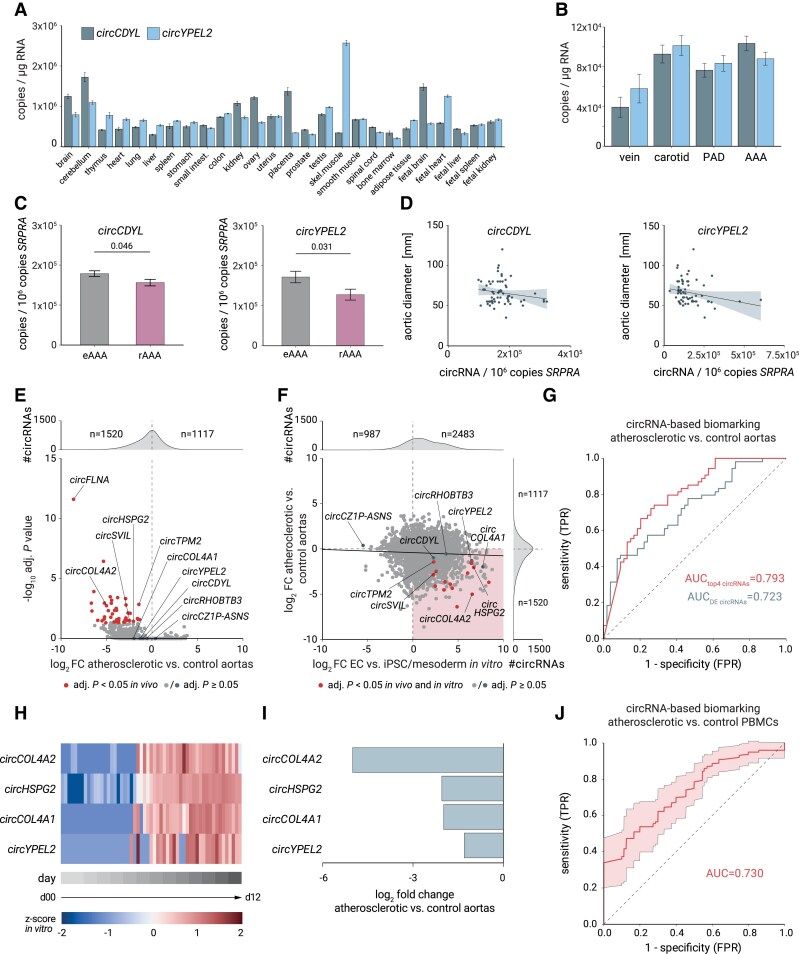
circRNAs that increase during vascular differentiation/quiescence are down-regulated in atherosclerosis and serve as biomarkers for vascular disease. (*A*) qRT-PCR analysis for top differentiation-associated circRNAs (*circCDYL*, *circYPEL2*) in various human adult and foetal tissues. Mean and SEM are shown. (*B*) circRNA expression by qRT-PCR in healthy or diseased blood vessel tissue samples from veins, carotids, PAD, and AAA. (*C*) Association of EC- or SMC-associated circRNA levels with abdominal aortic tissue samples from patients with elective repair surgery (eAAA) (*n* = 14) or emergency ruptured aortas (rAAA) (*n* = 49). (*D*) Correlation of AAA disease severity (aortic diameter dilation) and circRNA copy number by qRT-PCR. (*E*) Volcano plot of changes in circRNAs detected by RNA-seq comparing atherosclerotic and healthy arterial tissue (*n* = 54 patients). Only circRNAs that we also detected by *in vitro* profiling during iPSC-EC differentiation are shown. (*F*) Correlation of RNA-seq-based differences in circRNA levels from arterial tissue profiling and *in vitro* EC differentiation. (*G*) Results of ROC analyses, showing the performance of all differentially expressed EC differentiation state-linked circRNAs from iPSC > EC *in vitro* profiling (grey) and the performance of top four circRNAs (red) as markers for classifying atherosclerotic from healthy arterial tissue (*n* = 54/54). (*H*) Heatmap showing expression increase of the four best-classifying circRNAs as *z*-scores during *in vitro* differentiation from iPSC to EC. (*I*) Expression of the four best-classifying circRNAs in atherosclerotic tissue decreased compared with healthy arterial tissue. (*J*) Expression levels of *circYPEL2* and circRNAs of *HSPG2* determined by RNA-seq of human PBMCs enables binary classification of atherosclerosis patients (*n* = 89) and non-atherosclerosis subjects (*n* = 77) with an AUC = 0.730 using 10-fold cross-validation. AAA, abdominal aortic aneurysm; PAD, peripheral artery disease; FPR, false positive rate; TPR, true positive rate.

We first profiled representative differentiation-associated circRNAs in various adult and foetal human tissues and found that they were expressed (*Figure 5A*). We also found that these circRNAs were present in human primary EC and SMC from different donors and different vascular beds (see Supplementary material online, *Figure S19*). In vascular cells, circRNAs were mainly found in the cytoplasm, with a certain fraction also in the nucleoplasm, as typical for circRNAs (see Supplementary material online, *Figure S20*). Of note, we detected robust expression of these circRNAs not only in healthy but also in diseased vessels, such as from patients with aneurysm or peripheral atherosclerosis (*Figure 5B*). Thus, differentiation-associated circRNAs from *in vitro* profiling were robustly detectable in human tissue and specifically also in vascular tissue from healthy and diseased individuals.

We then investigated the abundance of vascular differentiation fate-associated circRNAs in tissues from individuals with and without vascular disease. We focused on aneurysm and atherosclerosis, as these diseases are known to involve not only dedifferentiation but also changes in SMC and EC proliferation and growth as major drivers of the disease processes.^12,13^ We found that *circCDYL* (*P* = 0.046) and *circYPEL2* (*P* = 0.031) showed lower levels in aneurysms of more severely diseased terminal AAA patients (stemming from patients undergoing emergency vessel surgery due to ruptured AAA; *n* = 14) compared with less advanced aneurysms (stemming from patients undergoing elective aneurysmal surgery; *n* = 49) (*Figure 5C*). Corroborating these findings, correlation analysis determined a trend for a negative relationship between aortic diameter as a measure of AAA disease grade and copies of the tested circRNAs, with circYPEL2 showing the strongest anticorrelation (*circCDYL*: *R* = −0.24, *P* = 0.28; *circYPEL2*: *R* = −0.24, *P* = 0.06) (*Figure 5D*).

Next, we investigated levels of EC/SMC-linked circRNAs in atherosclerotic and healthy arterial tissue samples from individuals undergoing cardiovascular surgery (*n* = 54). To this end, we performed RNA-seq from the tissues and quantified the expression of those subsets of the 3476 and 2246 circRNAs we found to be associated with differentiation to vascular EC and SMC, respectively. Importantly, we found a significant global down-regulation of circRNA levels in the atherosclerotic state (1.4- and 1.6-fold down for EC and SMC circRNAs) (*Figure 5E*; Supplementary material online, *Figure S21A* and *Tables S9* and *S10*). Comparison of the *in vitro* and the *in vivo* circRNA data from RNA-seq profiling revealed a major global change (*Figure 5F*, red quadrant), with up-regulation in vascular differentiation and down-regulation in atherosclerosis (*Figure 5F*; Supplementary material online, *Figure S21B*).

We then explored the predictive value of the identified vascular differentiation-linked circRNAs for molecularly characterizing atherosclerotic tissue *in vivo*. To this end, we performed ROC analyses by machine learning. The most informative circRNAs were identified by (i) using different feature selection methods and (ii) by testing them combinatorially using a SVM. We evaluated the AUC using our transcriptomic data set from arterial tissue profiling. We found that circRNA profiling using the entire set of differentially expressed circRNAs from our iPSC differentiation model conferred a substantial predictive value for atherosclerosis disease state profiling in tissue samples with an AUC of 0.723 (*Figure 5G*). The highest discrimination was obtained by a set of four circRNAs stemming from the *Perlecan* (*HSPG2*), the bidirectionally regulated *Collagen Type IV Alpha 1*/*2* locus (*COL4A1*, *COL4A2*) and the *Yippee-like YPEL2* gene locus. These four circRNAs discriminated between healthy and atherosclerotic aortic tissue *in vivo* with an AUC of 0.793 (*Figure 5G*). We are studying these circRNAs are molecular markers of aortic tissue state, and in their operational use as biomarkers, they do not necessarily have to be functional regulators of biological processes,^45^ which is consistent with the absence of vascular cell phenotypes after exemplary knockdown of *circYPEL2* (see Supplementary material online, *Figure S22*).

Therefore, we first continued to assess the identified four circRNAs in their potential capacity as operational non-diagnostic biomarkers of tissue state. All these four circRNAs stem from the class of circRNAs whose expression increased during vascular differentiation in our original *in vitr*o profiling (*Figure 5H*) and decreased in the atherosclerotic state compared with the healthy state (*Figure 5I*). To determine from which cell types the circRNAs derived *in situ*, we resorted to SmartSeq2-based single-cell transcriptomic data from 7688 cells from carotid atherosclerotic plaques derived from 15 patients.^46^ By providing our vascular BSJ library (*Figure 1*) as resource for circRNA mapping in single cells, we detected 2757 circRNAs in ECs, SMCs, macrophages, T cells, or B cells, including the four circRNAs from our signature (see Supplementary material online, *Figure S23A*–*F*). For these cell types, we found an enrichment in the combined cluster of ECs and SMCs for *circCOL4A1* (log_2_FC = 4.1, adjusted *P* = 1.0 × 10^−54^), *circCOL4A2* (log_2_FC = 4.2, adjusted *P* = 2.0 × 10^−66^), and *circHSPG2* (log_2_FC = 4.7, adjusted *P* = 2.0 × 10^−101^) (see Supplementary material online, *Figure S23G* and *Table S11*). *circYPEL2* was more ubiquitously expressed and is also present in numerous other non-vascular cell types, as documented in the circSC library.^47^ All four circRNAs had low BSJ read counts per cell and were expressed in only 1.3–37.6% of ECs and SMCs (see Supplementary material online, *Figure S23G*,*H*), in contrast to their more robustly and broadly expressed host mRNAs (see Supplementary material online, *Figure S23I* and *J*). This indicates that although the single-cell analysis described above is possible in principle, it operates at the limit of detectability, and cell-type specificity and enrichment must be interpreted accordingly. In summary, our signature circRNAs distinguish the state of atherosclerotic blood vessels and, with vascular tissue, originate largely from ECs and SMCs.

Finally, we explored whether any of the four identified circRNAs from our signature, apart from basic research-oriented tissue analysis, could also be useful in a clinically relevant diagnostic setting. Therefore, we explored circRNA levels in PBMCs from an independent cohort of *n* = 89 atherosclerosis patients and *n* = 77 individuals without atherosclerosis. We quantified the levels of the 4071 robustly expressed circRNAs from our initial vascular circRNA profiling using RNA-seq (see *Figure 1C*; Supplementary material online, *Table S12*). Of the four circRNAs with the highest discriminatory ability concerning atherosclerosis in aortic tissues (see *Figure 5G*), circ*COL4A1* and circ*COL4A2* were not expressed in PBMCs, but circRNAs from *YPEL2* and *HSPG2* were detectable. Binary classification with 10-fold cross-validation showed that circRNAs from *YPEL2* (chr17: 57 430 575–57 430 887) and *HSPG2* (chr1: 22 160 308–22 162 130; chr1: 22 206 599–22 207 995; chr1: 22 222 414–22 222 803) discriminated atherosclerotic from non-atherosclerotic individuals by our blood PBMC profiling with an AUC of 0.730 (*Figure 5J*).

Taken together, a large part of circRNAs identified during EC and SMC differentiation were detectable in human vascular tissues from different vascular beds *in vivo*, and some of these can even be quantified in blood PBMCs. A specific set of circRNAs was capable of distinguishing atherosclerotic from healthy arterial tissue in individual patients, and circRNAs from *YPEL2* and *HSPG2* served to distinguish patients with atherosclerosis from non-atherosclerosis individuals by blood sampling.

## 4. Discussion

In this study, and with a systems view of disease processes, we provide detailed comparative expression profiles of circRNAs during the differentiation of human iPSC to vascular EC and SMC, in aortic tissue, and in blood PBMC samples.

A major finding was that circRNAs from our profiling detected atherosclerosis status in blood PBMC samples of patients. We have narrowed the thousands of detectable circRNAs from global profiling to the most informative. This biomarker signature comprises circRNAs from *YPEL2* and *HSPG2*.

In our study, many circRNAs followed linear splicing rates, but ∼30% of all circRNAs in EC and SMC showed unique changes deviating from linear mRNA changes, which corresponds to approximately a hundred circRNAs showing specific alterations in CLRs. Given this complexity, our approach, including machine learning, generally highlights the granular features in circRNAs and their potential for health and disease profiling.

### 4.1 Global vascular circRNA changes are a salient feature for vascular disease biomarking

By tracking circRNA diversity, levels, and effect sizes during vascular differentiation, we revealed a global increase in circRNAs associated with a decline in proliferation-linked splicing. Global circRNA changes *per se* are reminiscent of some previous reports of global induction of circRNAs in non-dividing differentiated neuronal tissues.^28,48^ However, these other studies referred to circRNAs with different identities and did not report on developmentally controlled upstream regulators of circRNAs. Previously reported global circRNA increases have been ascribed to passive macromolecular accumulation, but so far, only correlatively.^27,28,49,50^ This reasoning is based on the fact that intracellular linear mRNA half-life is only 4–9 h,^51^ whereas circRNA half-life is 1–2 days.^52,53^ Therefore, over time, circRNAs should passively accumulate more than linear RNA in non-dividing cells. This may be particularly important in the vasculature: not only does vascular EC and SMC differentiation coincide with withdrawal from cell cycling but also with metabolic changes and metabolic rerouting actively enforces quiescence.^54^ Consequently, 99% of ECs in blood vessels *in vivo* are quiescent in terms of core cell cycle and growth activity.^54^ Indeed, the half-life of EC is 6 years when measured by ^14^C labelling in the heart.^55^ Our study does not formally rule out that circRNAs might be passively accumulating more than host mRNAs over periods much longer than our experiments (e.g. weeks or months of quiescence in blood vessels *in vivo*). Nonetheless, our data from contact inhibition, serum starvation, or MYC inhibition reinforce that circRNAs may be controlled more actively by cell proliferation rate. In particular, we show that MYC, an essential anabolic growth activator, affects circRNA levels. The finding that MYC affects circRNAs is broadly relevant because MYC is part of a larger vascular regulatory node. Thereby, VEGF, NOTCH, and FOXO1 suppress MYC to lock in vascular endothelial quiescence.^37^ MYC is limiting for proliferative capacity,^31^ and we suggest that MYC adjusts the level of RNA-related processes (including splicing and backsplicing) to the current biosynthetic cellular demand to support a certain level of cell proliferation (*Figure 4*). We, therefore, propose a molecular model of active global circRNA control by cell proliferation rate, which is not equivalent to but is also not mutually exclusive with the passive dilution model.

The reason selected circRNAs decrease during atherosclerosis in blood PBMC needs to be determined by future work. We do not have direct evidence that a decrease in our signature stands for a proliferative stimulus or dedifferentiation in either tissue or blood *in situ*. Our *in vitro* data only show that proliferative stimulation can, in principle, cause a decrease in circRNAs independent of differentiative changes in usually quiescent vascular cell states. *In situ*, the situation will be more complex, and proliferation and differentiation pathways or inflammation pathways will likely jointly contribute to the modulation of circRNA and circRNA host gene levels. This may also explain why the effect sizes of circRNA changes *in viv*o are larger and not identical to our *in vitro* experiments with cultured cells, where we modelled only certain aspects of proliferative control. Furthermore, differences in experimental design of achieving quiescence (e.g. serum levels vs. culture density) are expected to mediate non-equivalent quiescence states,^56^ which likely affected effect sizes of circRNA changes. Also, it must be taken into consideration that cell density impacts not only the cell cycle but also affects a number of other cell–cell signalling, biomechanical, migratory, and cell-size-dependent pathways that can impinge on molecular networks with influence on cell identity, at least to some degree. Thus, cell cycling and identity are never completely separable *in vitro*, either, and which of these above signals control the tested circRNAs and host genes will be important to determine in future work.

Our presented single-cell profiling using SmartSeq2 starts to address the origin of our signature circRNAs in major cell types of the vascular wall, in principle. The recorded low levels of circRNAs per single cell and the relatively small fraction of circRNA-expressing cells within a given cell type indicate that, despite the substantial overall depth of sequencing, current single-cell RNA-seq technology might only be scratching on the surface of information potentially embedded in circRNA profiling. New more sensitive (single-cell) circRNA-seq detection technologies would be helpful to increase the number of single cells with detectable circRNAs. This would be required to more robustly determine from which specific sub-cell types and activation states in blood vessel tissue or blood PBMC our circRNAs originate. However, most other current methods for single-cell RNA-seq, including those that were used to investigate linear mRNA changes during vascular cell and blood cell transitions,^57–60^ are unfortunately not suited to capture circRNAs.^47^ Furthermore, live imaging or genetic tagging *in situ* would be required to address a scenario in which circRNAs from any tissue were released into the blood. Additionally, immune cells are known to take up cell-free circRNAs,^61^ which complicates the hunt for the origin of changes in circRNAs in blood samples. In another testable hypothesis from our study, genetic atherosclerosis risk variants that affect gene expression and splicing with the same direction across different organ systems^62^ might be one of the reasons why *circHSPG2* and *circYPEL2* showed a shared down-regulation in both atherosclerotic blood vessel tissue and blood PBMC. Alternatively, these two genes, especially *YPEL2*, might be expressed with little tissue specificity in many cell systems and be linked to proliferative changes more generically, which was substantiated by the finding that *circYPEL2/YPEL2* were also increased in maturing cardiomyocytes.

### 4.2 circRNA host gene control is critical for circRNA changes

Our study shows that up- and down-regulation of circRNA levels mirrors, to a large extent, the changes in linear host RNA expression, for both up- and down-regulation. This is unexpected, considering the previously reported antagonism between linear splicing and backsplicing, where gene induction should lead to a decrease in circRNAs.^5–7,50,63–65^ Our DNA motif analyses also point to a positively determining role of host gene expression for circRNAs and show that circRNAs that increase during EC differentiation can be captured by features in their host loci (*Figure 4*). These include motifs for the EC fate-regulating GATA2 or NOTCH1, which are known to be activated and required for adhesion between specific EC subtypes,^66^ and VEGFA-dependent arterialization.^67^ Interestingly, the other identified transcription factors for our EC circRNA host genes are also known EC drivers. In particular, ERG, JUN, and EP300 are known to cooperate in the activation of endothelial super-enhancers, and such regions carry risk alleles for CAD.^30^ We have not investigated whether EC circRNAs were related to enhancer activity in our system, but it is conceivable that the identified coactivators may activate host genes and induce or reduce splice site utilization in a context-dependent manner because of the expected kinetic coupling between transcription elongation rate and spliceosome function.^64^ These transcription factors could equally open chromatin around certain introns and exons, which then regulates RNA splicing.^68^

A major finding of our study was that while circRNA levels increased, many splice factors with known roles as linear splicing enhancers, such as SRSF molecules, decreased during differentiation. Diminished linear splicing is known to be a substrate for more backsplicing.^7,65^ Splice factors from our study included *SRSF1* and *SRFS2*, two archetypal linear splicing enhancers. SRSF proteins have broad functions in transcription, RNA transport, and translation and are oncogenes that affect viability and proliferation,^69^ making it difficult to assess whether and how SRSFs directly affect circRNA backsplicing. Yet, *SRSF1* has also already been knocked down in cell lines in humans and the fruit fly.^7,65^ Loss of *SRSF1* or other core spliceosome factors such as SF3B, which we also picked up, caused repression of linear splicing and concomitant activation of backsplicing.^7,65^ This validates our model for circRNA regulation by splice factors, in principle. Given that we detected coordinated changes in many splice factors in our system (*Figure 4*), we expect, however, that regulatory dependencies will be more complex in differentiating EC and SMC compared with static cell conditions in cell lines. We also do not rule out other scenarios. For example, cell quiescence could be another independent determinant, as it can shape global chromatin dynamics, and altered chromatin state propagation can secondarily change how EC transcription and splicing factors act on gene loci.^33^

Overall, it becomes clear from our study that there is no single molecular determinant of changes in differentiation-linked circRNA levels. Instead, a complex compendium of DNA and RNA-regulatory factors is at work.

### 4.3 circRNAs as novel biomarkers for vascular disease

The set of four circRNAs in our biomarker signature used for the classification of healthy and diseased states in blood PBMC has not previously been reported as a group of biomarkers for cardiovascular disease. Some of these genes have been associated with vascular disease independently. For example, the heparan sulfate proteoglycan *HSPG2* and the collagens *COL4A1/2* serve as atheroprotective factors in cardiovascular tissue and repeatedly appeared as CAD risk loci in human genome-wide association studies.^70,71^ Thus, while not an aim of our study, one testable hypothesis is that these three circRNAs are not only biomarkers but might also be more intimately involved in CAD pathomechanisms. Proteins encoded by these three loci promote the structural integrity of vessel walls and mediate antiproliferative, differentiative, and anti-inflammatory signalling in EC and SMC,^70,71^ but circRNAs could be functional in multiple independent ways.^9^ Testing the function of circRNAs from *HSPG2*, *COL4A1*, and *COL4A2* was beyond the scope of our study because we detected dozens of distinct circRNAs for each locus (39, 23, and 19 distinct circRNA species, respectively), showcasing the dimension of molecular information embedded in circRNA splicing decisions. The fourth locus is *YPEL2* from the larger Yippee family of proteins and expresses a single circRNA. The YPEL2 protein is located at the centrosome and mitotic spindle and has been linked to cell proliferation (mostly based on overexpression analysis)^72^ but is generally not well studied, and its function in the cardiovascular system is unknown. We knocked down *circYPEL2* but did not find noticeable phenotypes in vascular cells. There might be redundancy in the *YPEL* family or phenotypes that we did not test, but our current data speak against strong regulatory effects by *YPEL2* and indicate that molecular information related to the presented circRNAs in these four genes provides a level of information that had not been obvious from existing profiling of linear gene products.

Besides the many complex immune cell changes, pathological changes in vascular tissue in cardiovascular disease are known, among others, to include dedifferentiation and proliferation of mature EC and SMC.^12,13^ Under atherosclerotic stress conditions *in vivo*, ECs become highly plastic as they are reprogrammed into mesenchymal, inflammatory, and immune cell-like phenotypes.^73^ In parallel, both endothelial-to-mesenchymal transition and SMC de- and transdifferentiation (e.g. into fibroblast-like or dysfunctional macrophage-like phenotypes) coincide with an aberrant re-entry into the cell cycle, again documenting the inherently complicating nexus between proliferation and differentiation.^12,13,74–76^ Already, we observed that vascular proliferative transitions coincided with changes in EC and SMC circRNAs but not with reactivation of mesodermal or stem cell-like circRNA profiles. This clarifies that we are not observing a full dedifferentiation to an embryonic-like mesodermal progenitor or stem cell state in adult vascular disease. However, in the absence of sufficiently sensitive single-cell circRNA-seq methods, the cellular heterogeneity in adult vascular tissue and blood, the contribution of multiple and interconverting cell types to disease, and the partial overlap in low-copy circRNA profiles make it difficult to delineate precisely which cellular transitions are represented by the observed decrease in circRNAs. Despite these challenges, our approach to mapping circRNAs is a new avenue with potential, and circRNAs from blood sampling emerge as meaningful *in vivo* markers for pathologically altered vascular disease states. It is at this point an equally interesting possibility that the global circRNA change is indicating beneficial restorative cellular responses in the face of adverse vascular stress.^77^

Overall, time-resolved comparative circRNA profiling helped to pinpoint circRNAs as biomarkers of vascular health and disease transitions in blood PBMC and put novel circRNAs, new host gene loci and their regulators on the map.

**Translational perspective**

The authors identified circular RNAs (circRNAs) in a stem cell-based vascular differentiation model and used them as a profiling tool for vascular disease. circRNA levels increased during endothelial cell and smooth muscle cell differentiation and decreased in arterial tissue and in blood peripheral blood mononuclear cell samples from patients with atherosclerosis. From the tens of thousands of circRNAs, the study distilled circRNAs that allowed blood sampling-based discrimination between healthy individuals and atherosclerosis patients with an area under the receiver operating characteristic curve of 0.73. The study highlights the potential of circRNAs for future diagnostic applications.

## Supplementary Material

cvaf013_Supplementary_Data

## Data Availability

The RNA-seq data sets supporting the conclusions of this article are available in the Gene Expression Omnibus (GEO) repository under IDs GSE210361 (bulk RNA sequencing) and GSE280608 (single-cell circRNA analysis), and the main results are provided in unshortened form in the Supplementary material. Primer and probe sequences are listed in Supplementary material online, *Table S13*. The code for the study has not been deposited in a public repository because no new methods were developed, and only pre-existing packages have been used.
