# Supplementary material for: Circular RNAs increase during vascular cell differentiation and are biomarkers for vascular disease
Source: Cardiovasc Res. 2025 Feb 4;121(3):405–23. doi: 10.1093/cvr/cvaf013 (PMC12038242; doi:10.1093/cvr/cvaf013)
Supplement: cvaf013_Supplementary_Data [file cvaf013_supplementary_data.zip › Northoff et al - Suppl Figures.pdf]

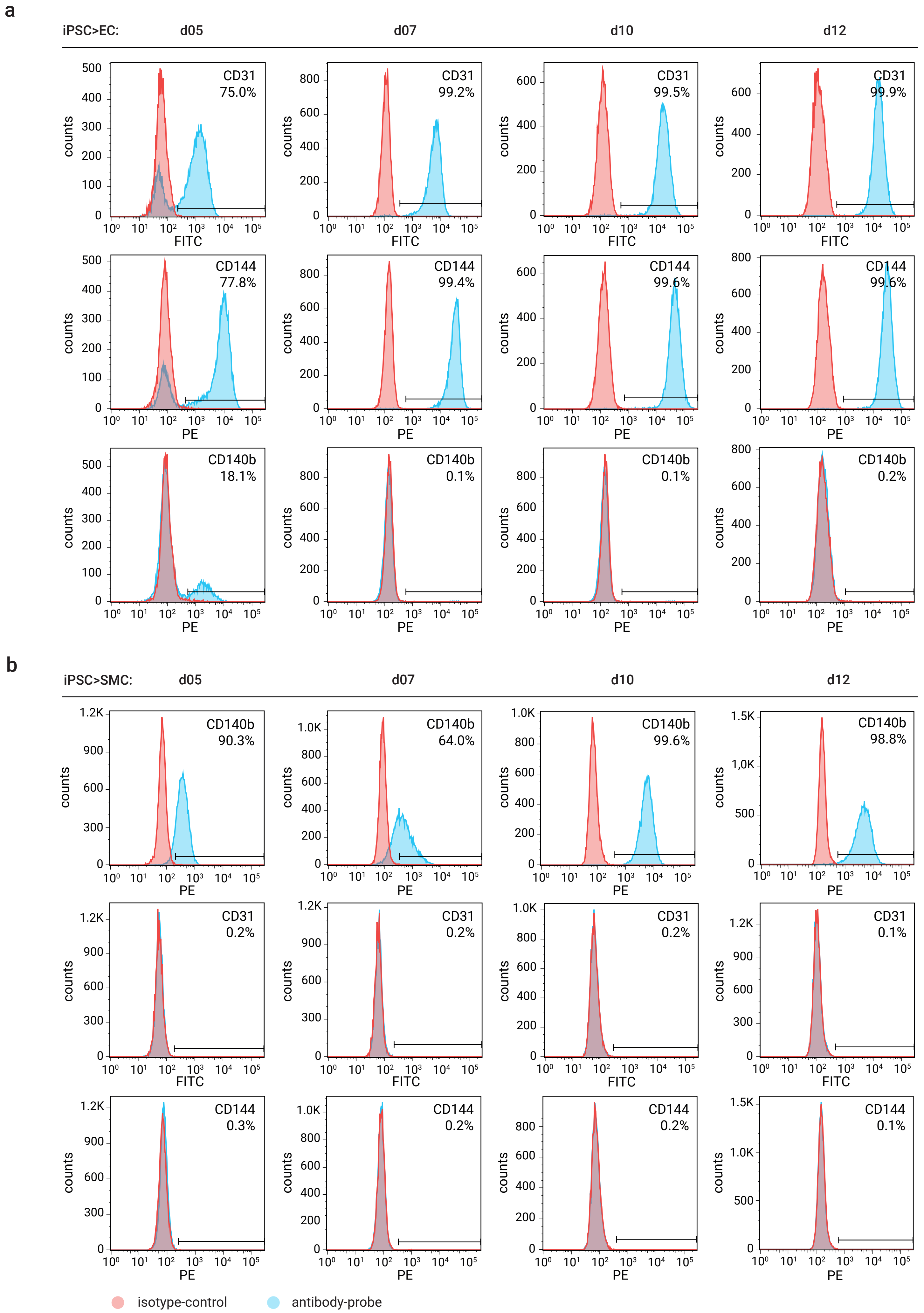
a
iPSC>EC:
d05
d07
d10
d12
CD31
75.0%
CD31
99.2%
CD31
99.5%
CD31
99.9%
CD144
77.8%
CD144
99.4%
CD144
99.6%
CD144
99.6%
CD140b
18.1%
CD140b
0.1%
CD140b
0.1%
CD140b
0.2%
counts
FITC
PE
b
iPSC>SMC:
d05
d07
d10
d12
CD140b
90.3%
CD140b
64.0%
CD140b
99.6%
CD140b
98.8%
CD31
0.2%
CD31
0.2%
CD31
0.2%
CD31
0.1%
CD144
0.3%
CD144
0.2%
CD144
0.2%
CD144
0.1%
counts
FITC
PE
isotype-control
antibody-probe

Fig. S1

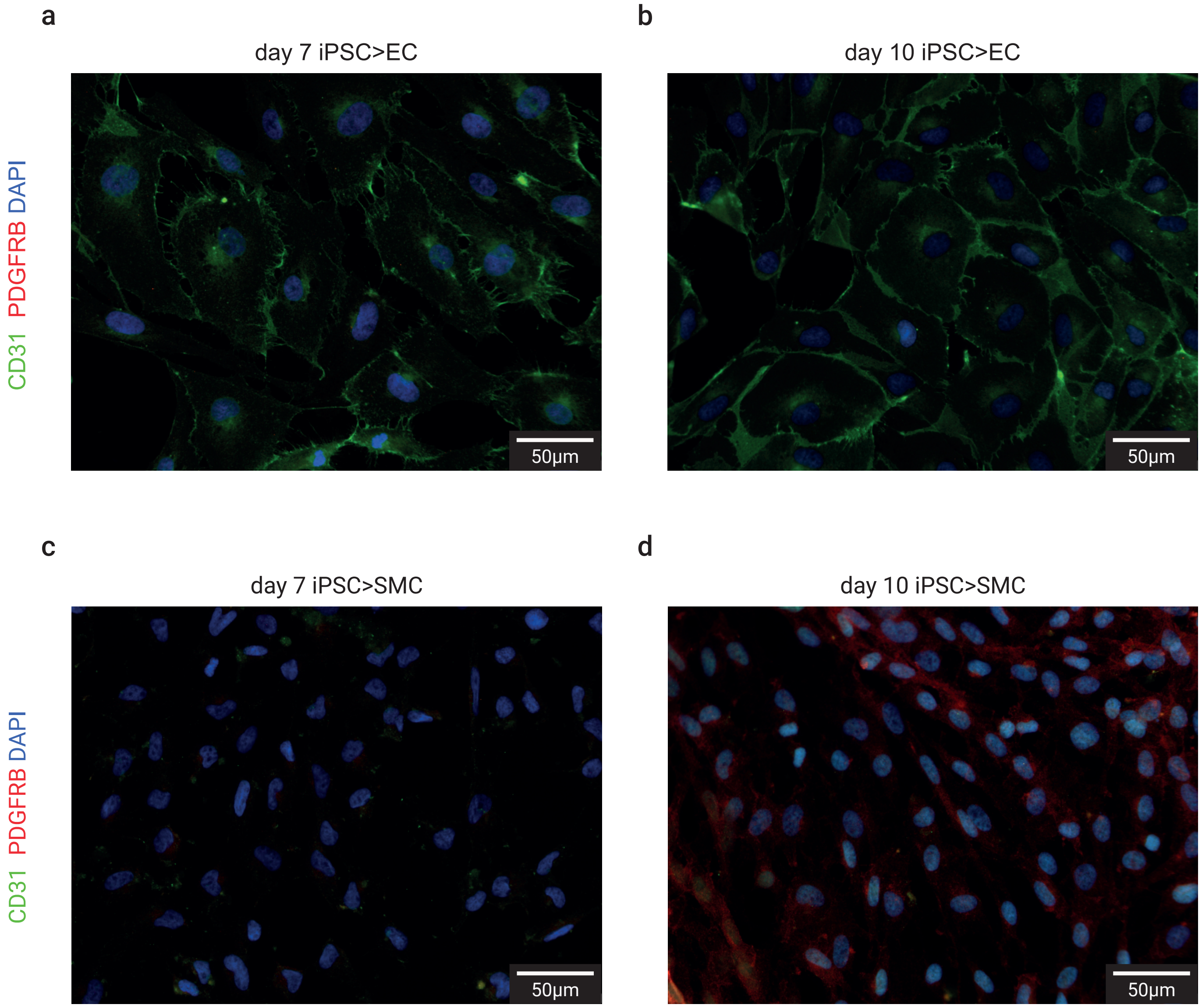
a
day 7 iPSC>EC
b
day 10 iPSC>EC
c
day 7 iPSC>SMC
d
day 10 iPSC>SMC
CD31 PDGFRB DAPI
CD31 PDGFRB DAPI
50μm
50μm
50μm
50μm

Fig. S2

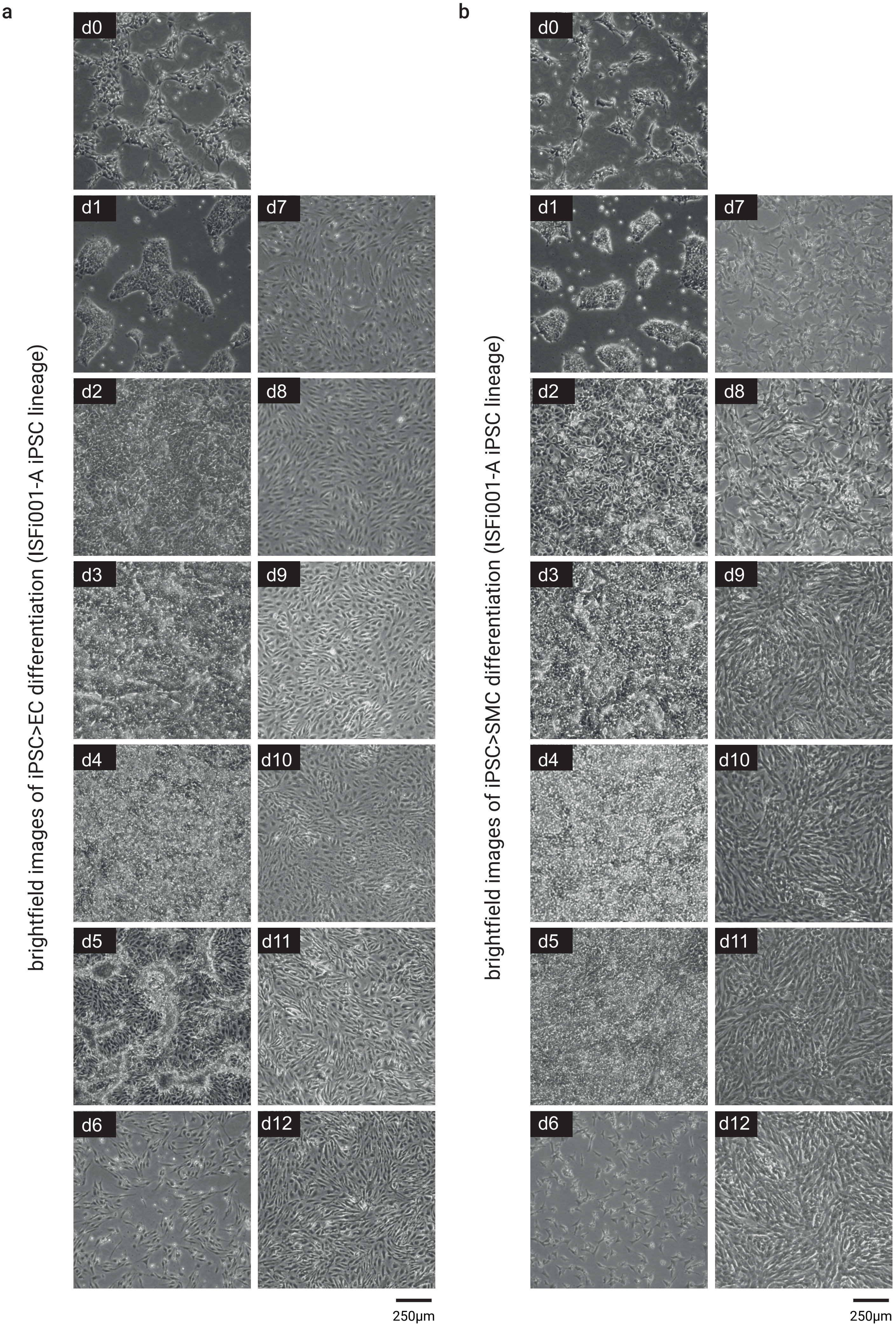
a
b
brightfield images of iPSC>EC differentiation (ISFi001-A iPSC lineage)
brightfield images of iPSC>SMC differentiation (ISFi001-A iPSC lineage)
d0
d1
d2
d3
d4
d5
d6
d7
d8
d9
d10
d11
d12
250µm
250µm

Fig. S3

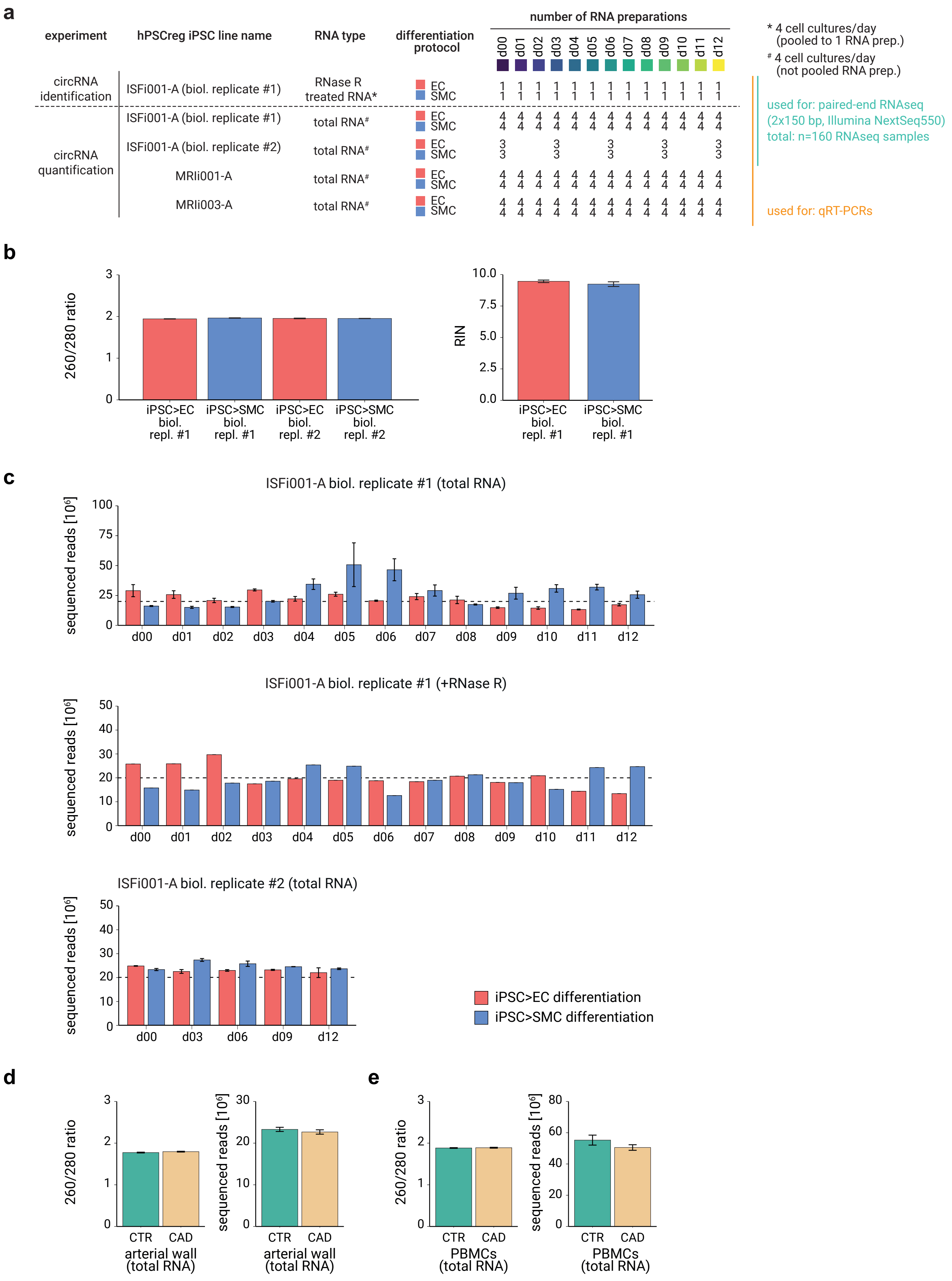
a
experiment
hPSCreg iPSC line name
RNA type
differentiation protocol
number of RNA preparations
d00 d01 d02 d03 d04 d05 d06 d07 d08 d09 d10 d11 d12
* 4 cell cultures/day (pooled to 1 RNA prep.)
# 4 cell cultures/day (not pooled RNA prep.)
circRNA identification
ISFi001-A (biol. replicate #1)
RNase R treated RNA*
EC
SMC
circRNA quantification
ISFi001-A (biol. replicate #1)
ISFi001-A (biol. replicate #2)
MRIi001-A
MRIi003-A
total RNA#
used for: paired-end RNAseq (2x150 bp, Illumina NextSeq550) total: n=160 RNAseq samples
used for: qRT-PCRs
b
260/280 ratio
RIN
iPSC>EC biol. repl. #1
iPSC>SMC biol. repl. #1
iPSC>EC biol. repl. #2
iPSC>SMC biol. repl. #2
c
ISFi001-A biol. replicate #1 (total RNA)
ISFi001-A biol. replicate #1 (+RNase R)
ISFi001-A biol. replicate #2 (total RNA)
sequenced reads [10⁶]
iPSC>EC differentiation
iPSC>SMC differentiation
d
e
CTR
CAD
arterial wall (total RNA)
PBMCs (total RNA)

Fig. S4

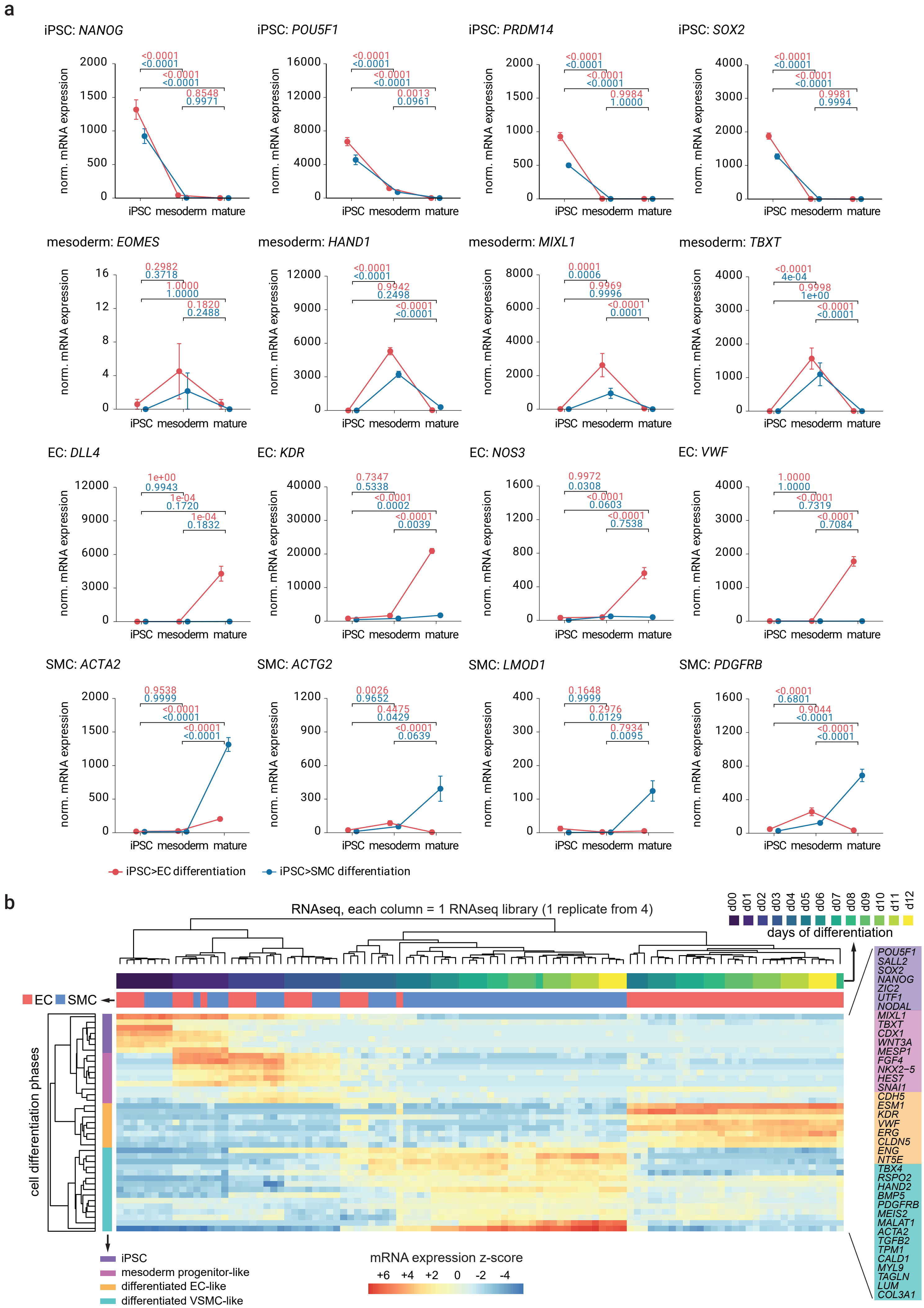
a
iPSC: NANOG
norm. mRNA expression
<0.0001
<0.0001
<0.0001
<0.0001
0.8548
0.9971
iPSC mesoderm mature
iPSC: POU5F1
<0.0001
<0.0001
<0.0001
<0.0001
0.0013
0.0961
iPSC: PRDM14
<0.0001
<0.0001
<0.0001
<0.0001
0.9984
1.0000
iPSC: SOX2
<0.0001
<0.0001
<0.0001
<0.0001
0.9981
0.9994
mesoderm: EOMES
0.2982
0.3718
1.0000
1.0000
0.1820
0.2488
mesoderm: HAND1
<0.0001
<0.0001
0.9942
0.2498
<0.0001
<0.0001
mesoderm: MIXL1
0.0001
0.0006
0.9969
0.9996
<0.0001
0.0001
mesoderm: TBXT
<0.0001
4e-04
0.9998
1e+00
<0.0001
<0.0001
EC: DLL4
1e+00
0.9943
1e-04
0.1720
1e-04
0.1832
EC: KDR
0.7347
0.5338
<0.0001
0.0002
<0.0001
0.0039
EC: NOS3
0.9972
0.0308
<0.0001
0.0603
<0.0001
0.7538
EC: VWF
1.0000
1.0000
<0.0001
0.7319
<0.0001
0.7084
SMC: ACTA2
0.9538
0.9999
<0.0001
<0.0001
<0.0001
<0.0001
SMC: ACTG2
0.0026
0.9652
0.4475
0.0429
<0.0001
0.0639
SMC: LMOD1
0.1648
0.9999
0.2976
0.0129
0.7934
0.0095
SMC: PDGFRB
<0.0001
0.6801
0.9044
<0.0001
<0.0001
<0.0001
iPSC>EC differentiation
iPSC>SMC differentiation
b
RNAseq, each column = 1 RNAseq library (1 replicate from 4)
d00 d01 d02 d03 d04 d05 d06 d07 d08 d09 d10 d11 d12
days of differentiation
EC SMC
cell differentiation phases
iPSC
mesoderm progenitor-like
differentiated EC-like
differentiated VSMC-like
mRNA expression z-score
+6 +4 +2 0 -2 -4
POU5F1
SALL2
SOX2
NANOG
ZIC2
UTF1
NODAL
MIXL1
TBXT
CDX1
WNT3A
MESP1
FGF4
NKX2−5
HES7
SNAI1
CDH5
ESM1
KDR
VWF
ERG
CLDN5
ENG
NT5E
TBX4
RSPO2
HAND2
BMP5
PDGFRB
MEIS2
MALAT1
ACTA2
TGFB2
TPM1
CALD1
MYL9
TAGLN
LUM
COL3A1

Fig. S5

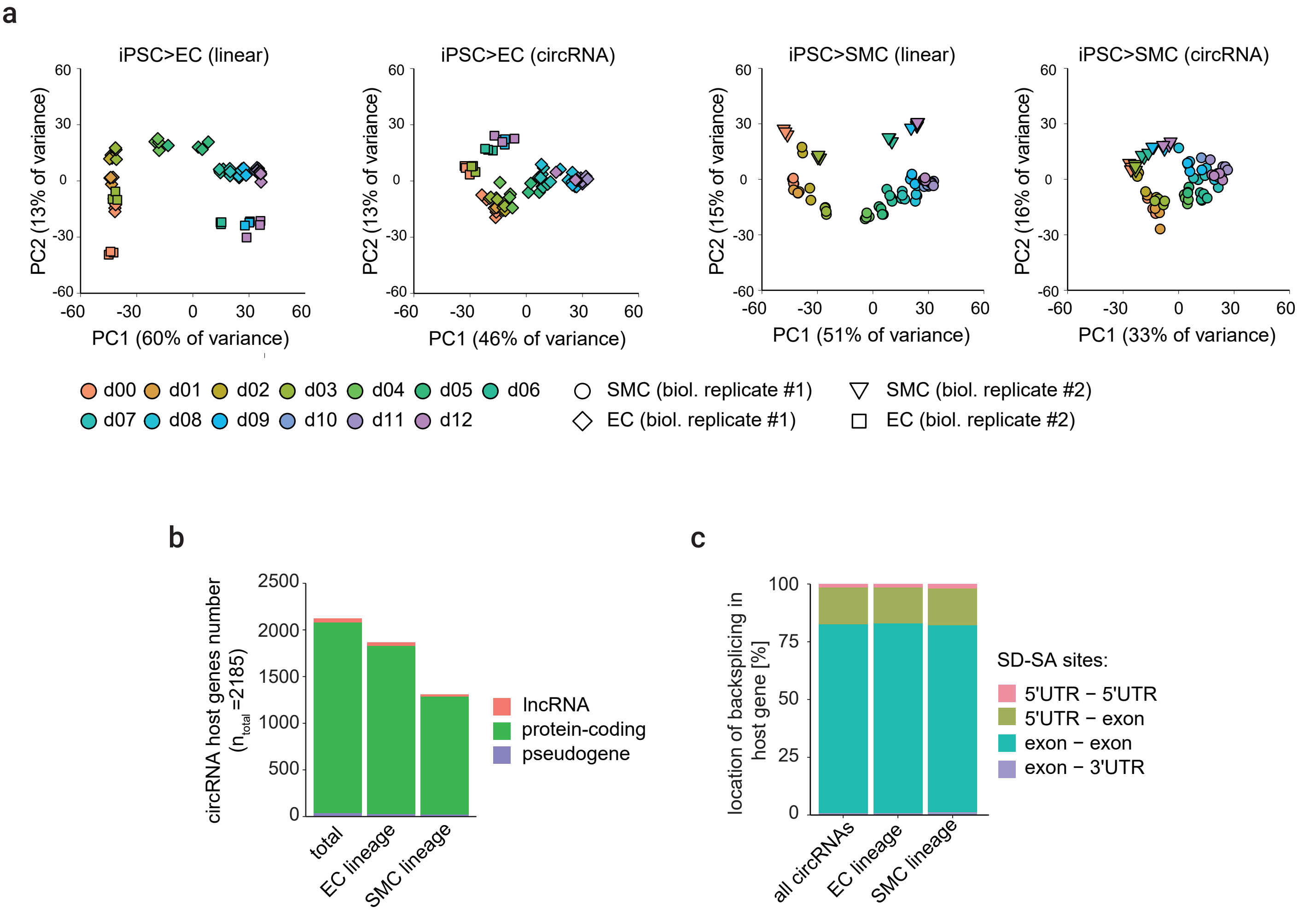
a
iPSC>EC (linear)
iPSC>EC (circRNA)
iPSC>SMC (linear)
iPSC>SMC (circRNA)
PC2 (13% of variance)
PC2 (13% of variance)
PC2 (15% of variance)
PC2 (16% of variance)
PC1 (60% of variance)
PC1 (46% of variance)
PC1 (51% of variance)
PC1 (33% of variance)
d00 d01 d02 d03 d04 d05 d06
d07 d08 d09 d10 d11 d12
SMC (biol. replicate #1)
SMC (biol. replicate #2)
EC (biol. replicate #1)
EC (biol. replicate #2)
b
circRNA host genes number
(n_{total}=2185)
lncRNA
protein-coding
pseudogene
total
EC lineage
SMC lineage
c
location of backsplicing in host gene [%]
all circRNAs
EC lineage
SMC lineage
SD-SA sites:
5'UTR − 5'UTR
5'UTR − exon
exon − exon
exon − 3'UTR

Fig. S6

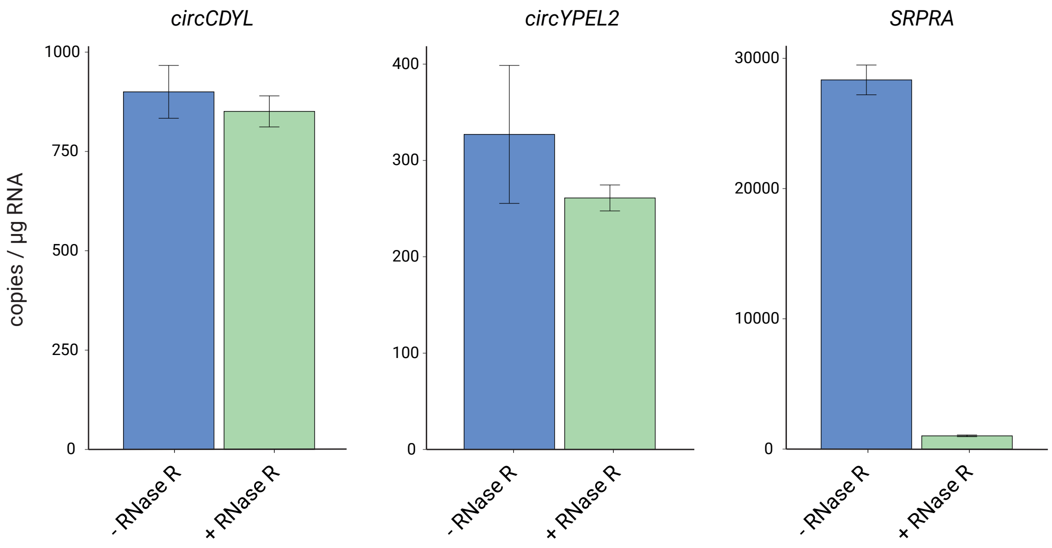

circCDYL
circYPEL2
SRPRA
copies / µg RNA
1000
750
500
250
0
400
300
200
100
0
30000
20000
10000
0
- RNase R
+ RNase R
- RNase R
+ RNase R
- RNase R
+ RNase R

Fig. S7

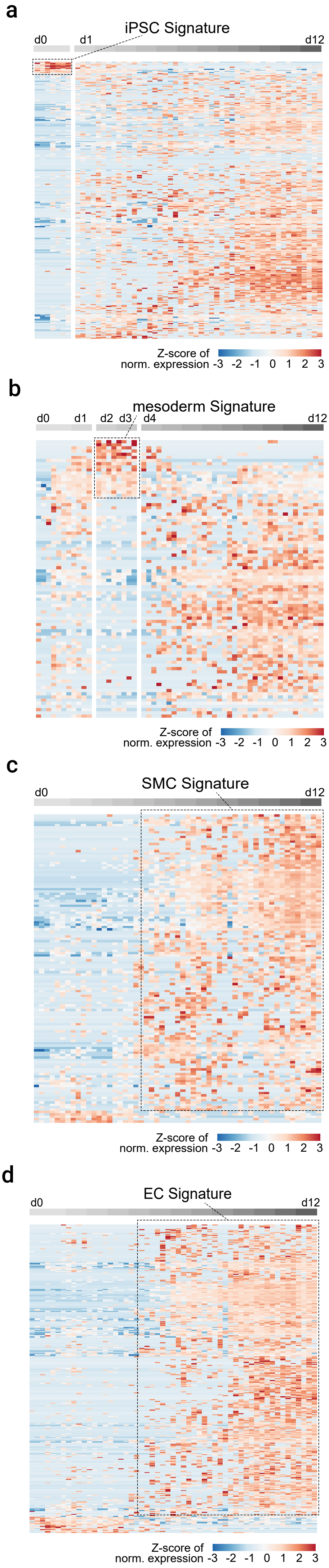

iPSC Signature

chrX:130889625−130902724
chr20:31389083−31393213
chr20:31383214−31388104
chr20:31375035−31376818
chr20:31368123−31376818
chr20:31376659−31380576
chr20:31368123−31375257
chr15:25320881−25328674
chr15:25330336−25332746
chr15:25304603−25307235
chr16:3900297−3901010
chr5:149754179−149754374

mesoderm Signature

chr22:29517344−29521404
chr16:50655512−50655619
chr16:50642204−50655619
chr16:50655512−50666319
chr9:92220581−92220795
chr4:108984778−109010413
chr7:97557393−97558089
chr4:108984778−109000770
chr6:26056053−26056299
chr6:26056122−26056299
chr8:144662675−144672251
chr17:34171549−34171990
chr17:34171753−34172014
chr17:34171753−34171912
chr19:3980838−3981450
chr1:8926337−8927309
chr17:34171840−34172014
chr17:34171708−34171822

SMC Signature

chr12:24678696−24678935
chr6:160819010−160831878
chr9:137666704−137676942
chr9:137674512−137676942
chr9:137619111−137620653
chr3:112348959−112357113
chr2:189859266−189866176
chr8:38947565−38948865
chr2:189872610−189873947
chr14:52495439−52527074
chr17:48271933−48272189
chr7:45960336−45960619
chr14:52485778−52486896
chr7:94040367−94041433
chr9:137619111−137666758
chr5:151052702−151054205
chr2:216242897−216242985
chr9:137676834−137677894
chr17:60741908−60742310
chr7:94052268−94053755
chr7:94054428−94055161
chr17:48265907−48266844
chr7:94039553−94039839
chr2:216248050−216248206
chr2:189866122−189867788
chr2:189864010−189864303
chr2:189859771−189860516
chr2:189871662−189872333
chr2:189859449−189859557
chr2:189859266−189859557
chr2:189861123−189862479
chr2:216229601−216230353
chr2:216232585−216240116
chr2:216256354−216259359
chr17:48268177−48268851
chr17:48266102−48266371
chr17:48265236−48266156
chr2:216271849−216272920
chr17:48267361−48267741
chr2:216279559−216293061
chr7:94048809−94054536
chr17:48265456−48266899
chr7:94043205−94045815
chr17:48266737−48266899
chr7:94037158−94041994
chr17:48266528−48266899
chr17:39974633−39975651
chr17:48267219−48267469
chrX:139865339−139866824
chr13:111109689−111114731
chr14:52508822−52509068
chr20:32981596−33001685
chr19:49425083−49425189
chr9:117835881−117836145
chr1:103540173−103548528
chr9:137696820−137698142
chr8:131164981−131193126
chr19:49407603−49409142
chr15:63351761−63351879
chr17:79813017−79813462
chr2:189874903−189875091
chrX:139866257−139866473
chr2:216279559−216279681
chr14:52520296−52527074
chr6:16326624−16328701
chr1:117944807−117984947
chr4:153332454−153333681
chr7:107566627−107566722
chr11:65269241−65269431
chr14:52486776−52494062
chr5:16877717−16877816
chrX:139865879−139866297
chrX:139866135−139866387
chr4:83791477−83802075
chr21:16386664−16415895
chr2:187503003−187506315
chr7:6509226−6513915
chr10:17277167−17277888
chr7:5567911−5568350
chr13:111156193−111156340
chr15:63349183−63351879
chr11:65266809−65267071
chr2:85133509−85133786
chr2:216251411−216259442
chr2:187511412−187516816
chr5:127607690−127611852
chr10:73579996−73585650
chr14:52520296−52520656
chr3:171969049−171969331
chr3:112326022−112335705
chr13:111158814−111158953
chr2:10931920−10933328
chr14:32559707−32586493
chr11:65267869−65268013
chr11:65266743−65266983
chr9:137582757−137582925
chr2:216245533−216245669
chr3:145838898−145842016
chr21:47545179−47545531
chr1:16891301−16893846
chr11:65267869−65268102
chr11:65267964−65268121
chr11:65272657−65272846
chr15:48757764−48760731
chr8:38911999−38940592
chr17:79813017−79817263
chr11:65267869−65268203
chr7:47520700−47568745
chrX:139865589−139865754
chr12:124071293−124074996
chr5:127685646−127693087
chr15:30033534−30064369
chr5:34796043−34818956
chr15:63336225−63354844
chr7:148711995−148716289
chr15:76152218−76165909
chr4:183836075−183836728
chr17:37070577−37070728
chr21:46910188−46913139
chr17:79804304−79805223
chr12:90035934−90049884
chr9:35685430−35689214
chr15:89448987−89453154
chr11:85725912−85742653
chr15:58971322−58974513

EC Signature

[illegible]

Fig. S8

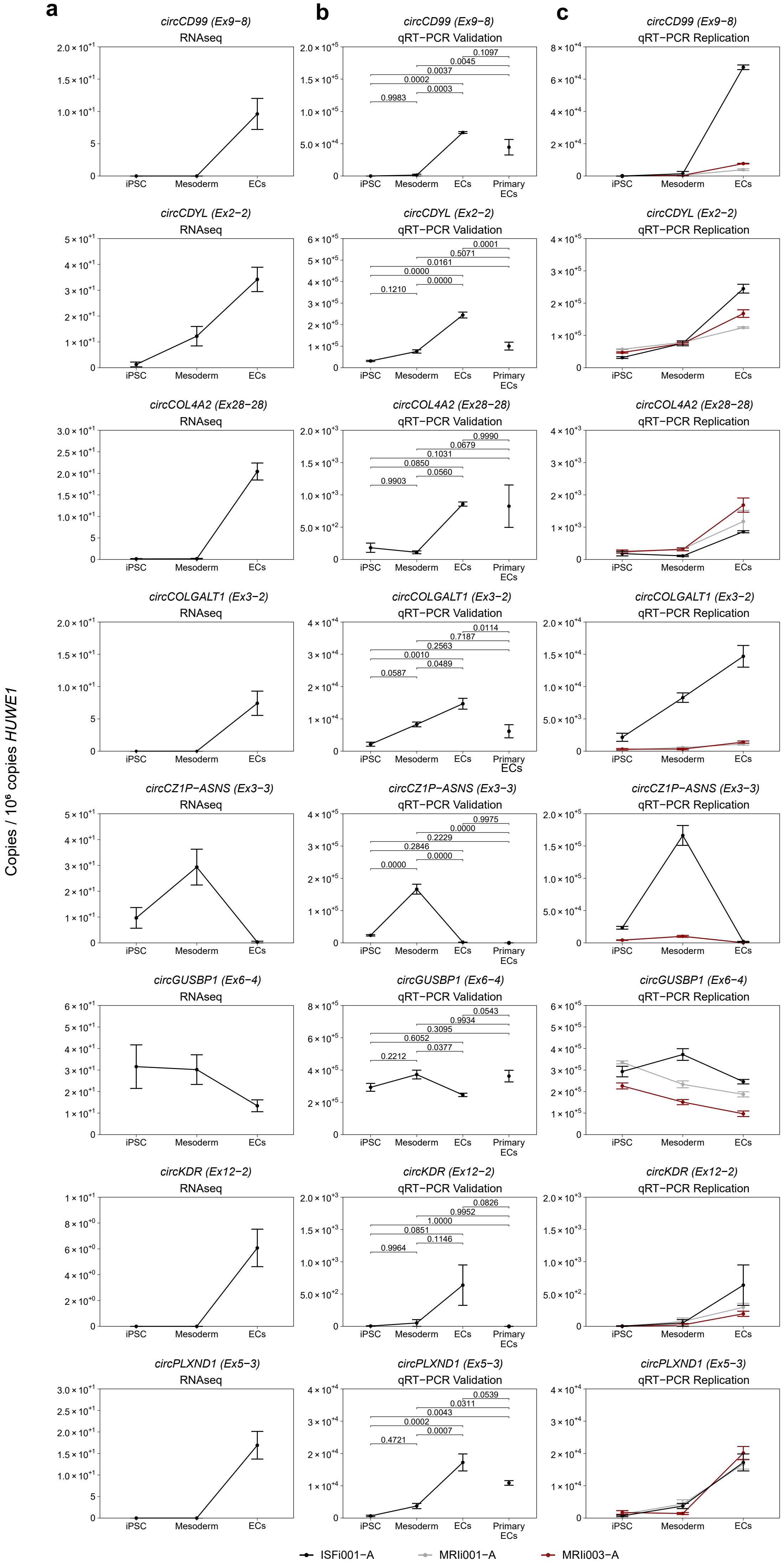
a
b
c
Copies / 10^6 copies HUWE1
circCD99 (Ex9−8)
RNAseq
qRT−PCR Validation
qRT−PCR Replication
0.1097
0.0045
0.0037
0.0002
0.0003
0.9983
circCDYL (Ex2−2)
0.0001
0.5071
0.0161
0.0000
0.0000
0.1210
circCOL4A2 (Ex28−28)
0.9990
0.0679
0.1031
0.0850
0.0560
0.9903
circCOLGALT1 (Ex3−2)
0.0114
0.7187
0.2563
0.0010
0.0489
0.0587
circCZ1P−ASNS (Ex3−3)
0.9975
0.0000
0.2229
0.2846
0.0000
0.0000
circGUSBP1 (Ex6−4)
0.0543
0.9934
0.3095
0.6052
0.0377
0.2212
circKDR (Ex12−2)
0.0826
0.9952
1.0000
0.0851
0.1146
0.9964
circPLXND1 (Ex5−3)
0.0539
0.0311
0.0043
0.0002
0.0007
0.4721
iPSC
Mesoderm
ECs
Primary ECs
ISFi001−A
MRIi001−A
MRIi003−A

Fig. S9

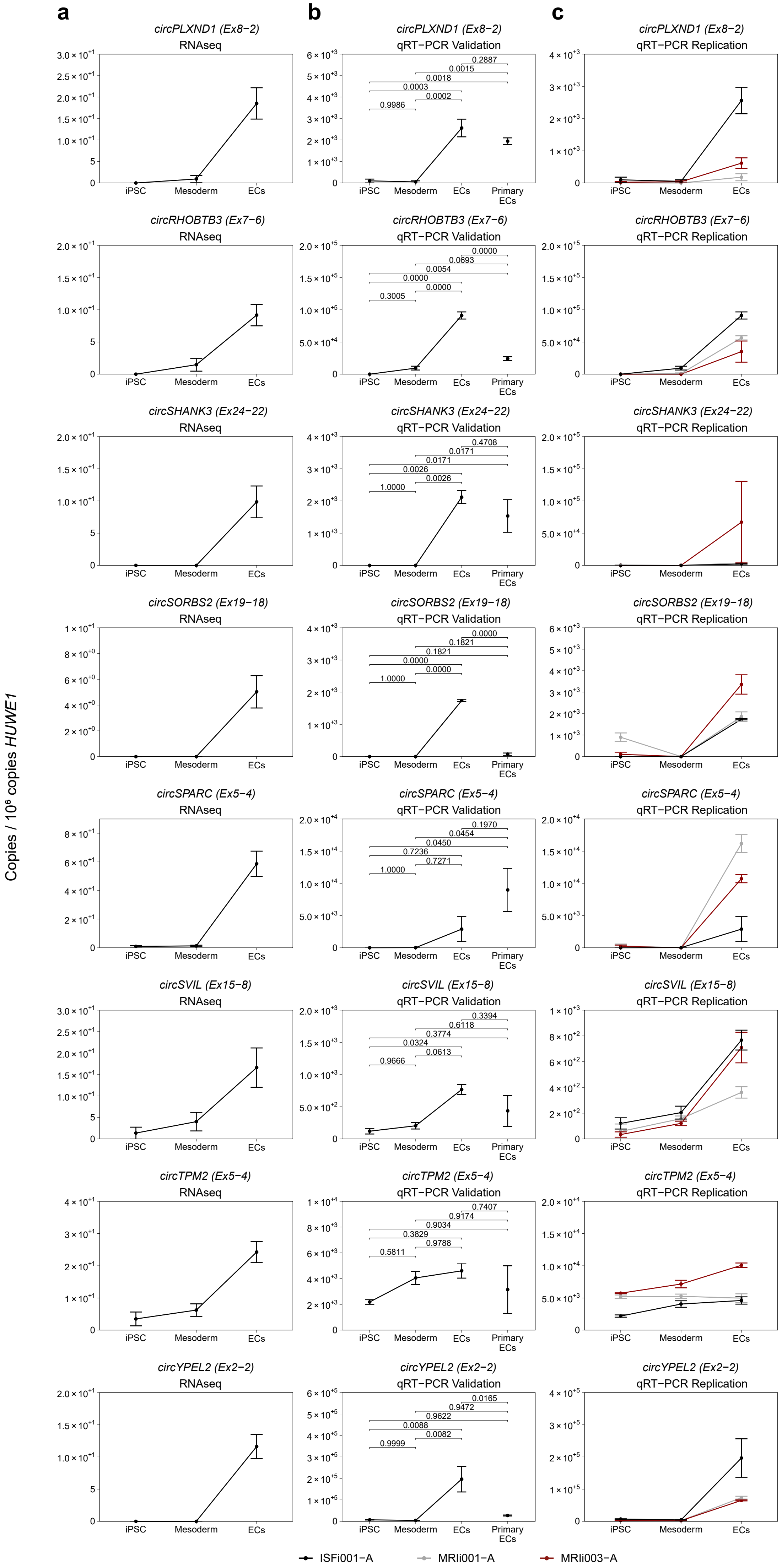

a
b
c
Copies / 10^6 copies HUWE1
circPLXND1 (Ex8−2)
RNAseq
qRT−PCR Validation
0.2887
0.0015
0.0018
0.0003
0.0002
0.9986
qRT−PCR Replication
circRHOBTB3 (Ex7−6)
RNAseq
qRT−PCR Validation
0.0000
0.0693
0.0054
0.0000
0.0000
0.3005
qRT−PCR Replication
circSHANK3 (Ex24−22)
RNAseq
qRT−PCR Validation
0.4708
0.0171
0.0171
0.0026
0.0026
1.0000
qRT−PCR Replication
circSORBS2 (Ex19−18)
RNAseq
qRT−PCR Validation
0.0000
0.1821
0.1821
0.0000
0.0000
1.0000
qRT−PCR Replication
circSPARC (Ex5−4)
RNAseq
qRT−PCR Validation
0.1970
0.0454
0.0450
0.7236
0.7271
1.0000
qRT−PCR Replication
circSVIL (Ex15−8)
RNAseq
qRT−PCR Validation
0.3394
0.6118
0.3774
0.0324
0.0613
0.9666
qRT−PCR Replication
circTPM2 (Ex5−4)
RNAseq
qRT−PCR Validation
0.7407
0.9174
0.9034
0.3829
0.9788
0.5811
qRT−PCR Replication
circYPEL2 (Ex2−2)
RNAseq
qRT−PCR Validation
0.0165
0.9472
0.9622
0.0088
0.0082
0.9999
qRT−PCR Replication
iPSC
Mesoderm
ECs
Primary ECs
ISFi001−A
MRIi001−A
MRIi003−A

Fig. S10

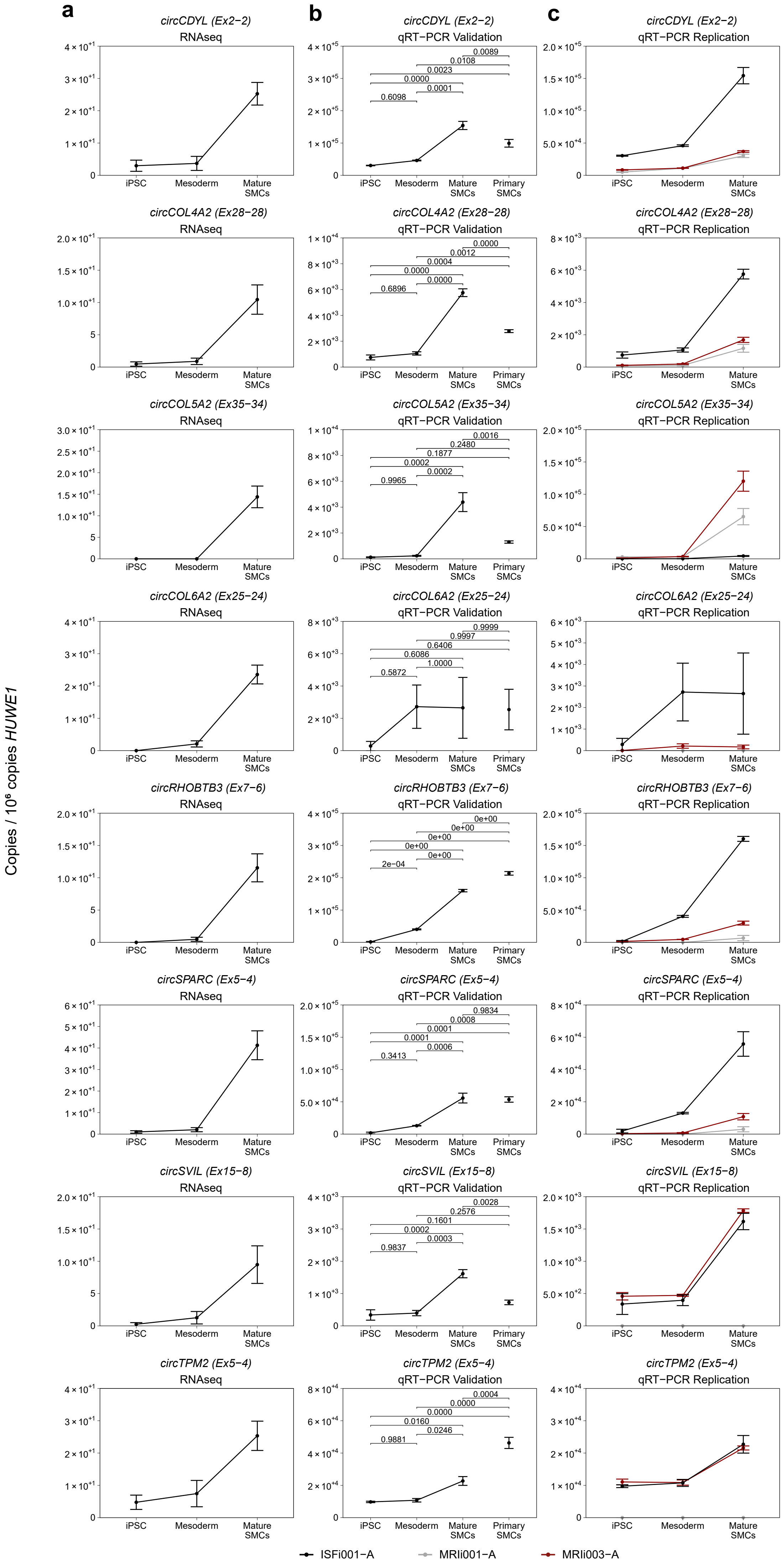
a
b
c
Copies / 10⁶ copies HUWE1
circCDYL (Ex2−2)
RNAseq
qRT−PCR Validation
qRT−PCR Replication
0.0089
0.0108
0.0023
0.0000
0.0001
0.6098
circCOL4A2 (Ex28−28)
0.0000
0.0012
0.0004
0.0000
0.0000
0.6896
circCOL5A2 (Ex35−34)
0.0016
0.2480
0.1877
0.0002
0.0002
0.9965
circCOL6A2 (Ex25−24)
0.9999
0.9997
0.6406
0.6086
1.0000
0.5872
circRHOBTB3 (Ex7−6)
0e+00
0e+00
0e+00
0e+00
0e+00
2e−04
circSPARC (Ex5−4)
0.9834
0.0008
0.0001
0.0001
0.0006
0.3413
circSVIL (Ex15−8)
0.0028
0.2576
0.1601
0.0002
0.0003
0.9837
circTPM2 (Ex5−4)
0.0004
0.0000
0.0000
0.0160
0.0246
0.9881
iPSC
Mesoderm
Mature SMCs
Primary SMCs
ISFi001−A
MRIi001−A
MRIi003−A

Fig. S11

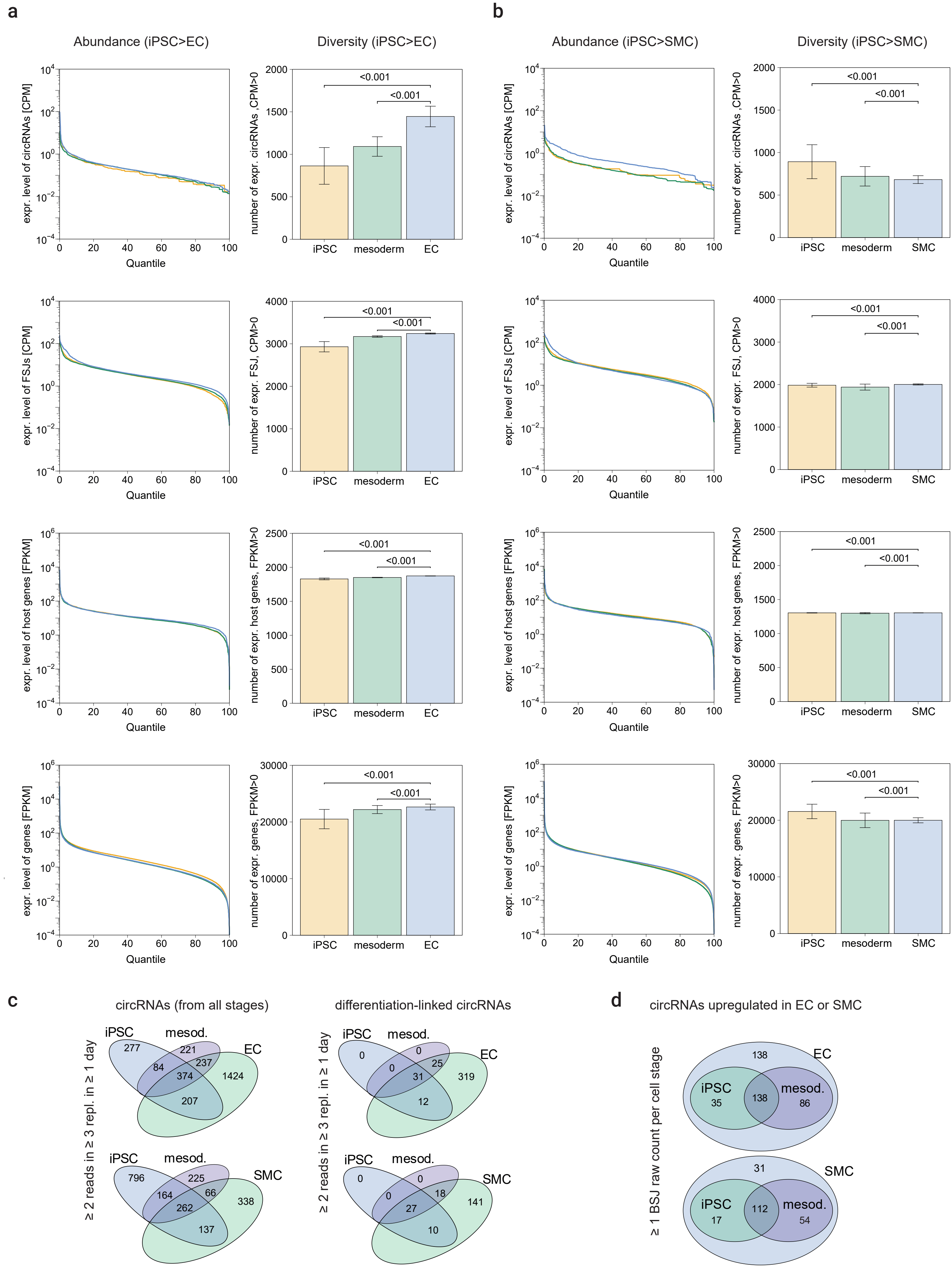
a
b
Abundance (iPSC>EC)
Diversity (iPSC>EC)
Abundance (iPSC>SMC)
Diversity (iPSC>SMC)
expr. level of circRNAs [CPM]
number of expr. circRNAs, CPM>0
expr. level of FSJs [CPM]
number of expr. FSJ, CPM>0
expr. level of host genes [FPKM]
number of expr. host genes, FPKM>0
expr. level of genes [FPKM]
number of expr. genes, FPKM>0
Quantile
iPSC
mesoderm
EC
SMC
<0.001
c
circRNAs (from all stages)
differentiation-linked circRNAs
≥ 2 reads in ≥ 3 repl. in ≥ 1 day
iPSC
mesod.
EC
SMC
277
221
84
237
374
1424
207
796
225
164
66
262
338
137
0
0
0
25
31
319
12
0
0
0
18
27
141
10
d
circRNAs upregulated in EC or SMC
≥ 1 BSJ raw count per cell stage
138
35
138
86
31
17
112
54

Fig. S12

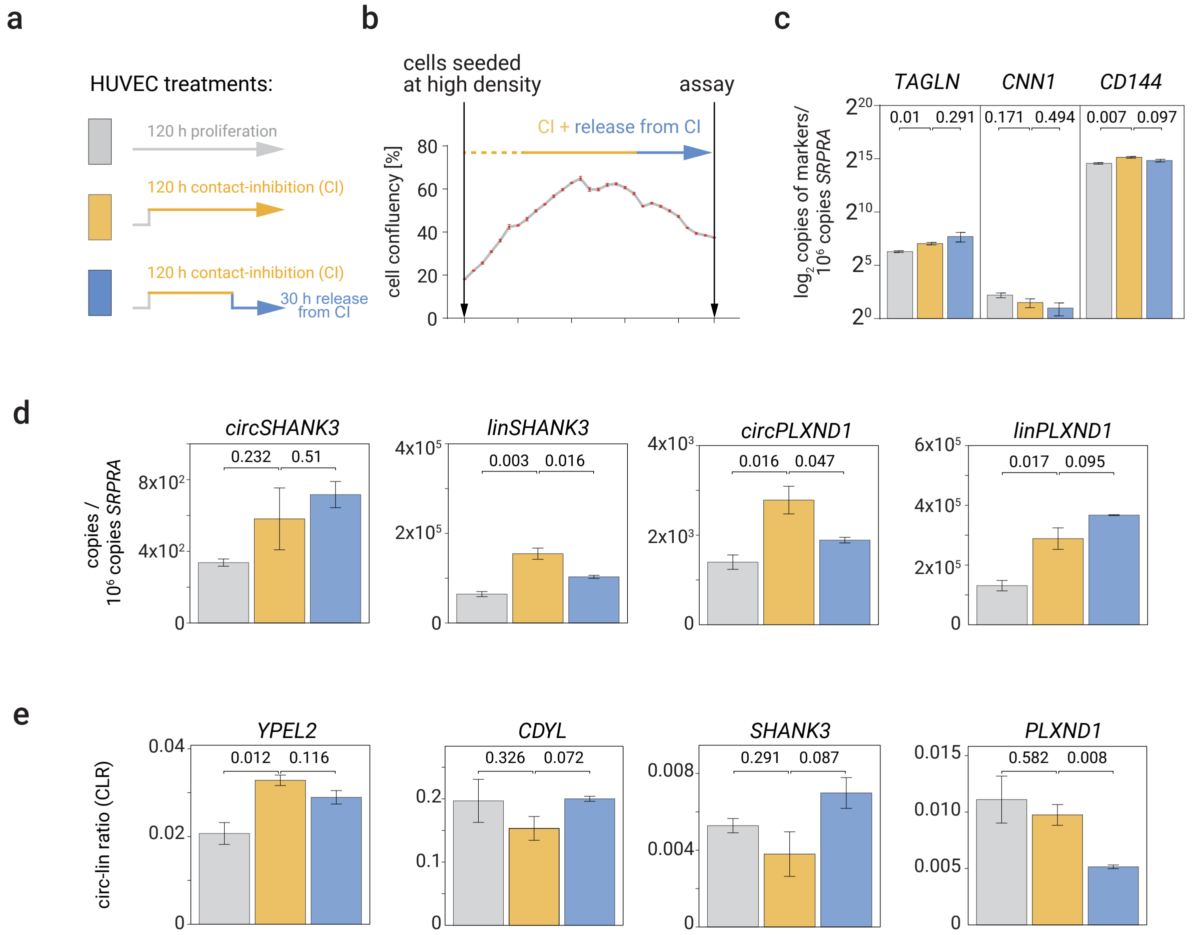
a
HUVEC treatments:
120 h proliferation
120 h contact-inhibition (CI)
120 h contact-inhibition (CI)
30 h release from CI
b
cells seeded at high density
assay
CI + release from CI
cell confluency [%]
c
TAGLN
CNN1
CD144
0.01 0.291 0.171 0.494 0.007 0.097
log2 copies of markers/ 10^6 copies SRPRA
d
circSHANK3
0.232 0.51
linSHANK3
0.003 0.016
circPLXND1
0.016 0.047
linPLXND1
0.017 0.095
copies / 10^6 copies SRPRA
e
YPEL2
0.012 0.116
CDYL
0.326 0.072
SHANK3
0.291 0.087
PLXND1
0.582 0.008
circ-lin ratio (CLR)

Fig. S13

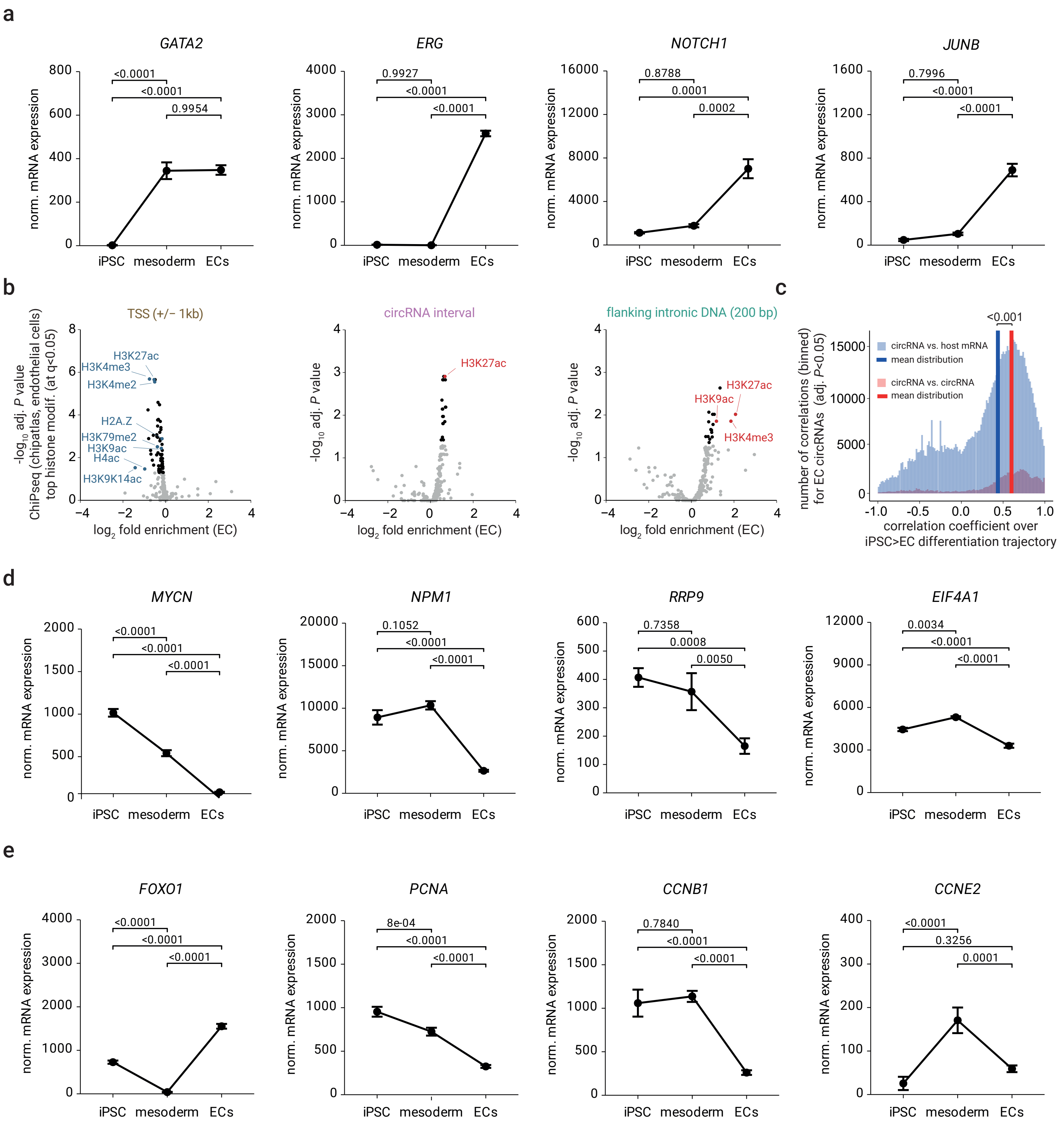

a
GATA2
ERG
NOTCH1
JUNB
norm. mRNA expression
<0.0001
<0.0001
0.9954
0.9927
<0.0001
<0.0001
0.8788
0.0001
0.0002
0.7996
<0.0001
<0.0001
iPSC mesoderm ECs
b
TSS (+/− 1kb)
circRNA interval
flanking intronic DNA (200 bp)
ChIPseq (chipatlas, endothelial cells) top histone modif. (at q<0.05)
-log10 adj. P value
H3K27ac
H3K4me3
H3K4me2
H2A.Z
H3K79me2
H3K9ac
H4ac
H3K9K14ac
H3K4me3
log2 fold enrichment (EC)
c
<0.001
circRNA vs. host mRNA
mean distribution
circRNA vs. circRNA
mean distribution
number of correlations (binned) for EC circRNAs (adj. P<0.05)
correlation coefficient over iPSC>EC differentiation trajectory
d
MYCN
NPM1
RRP9
EIF4A1
<0.0001
<0.0001
<0.0001
0.1052
<0.0001
<0.0001
0.7358
0.0008
0.0050
0.0034
<0.0001
<0.0001
e
FOXO1
PCNA
CCNB1
CCNE2
<0.0001
<0.0001
<0.0001
8e-04
<0.0001
<0.0001
0.7840
<0.0001
<0.0001
<0.0001
0.3256
0.0001

Fig. S14

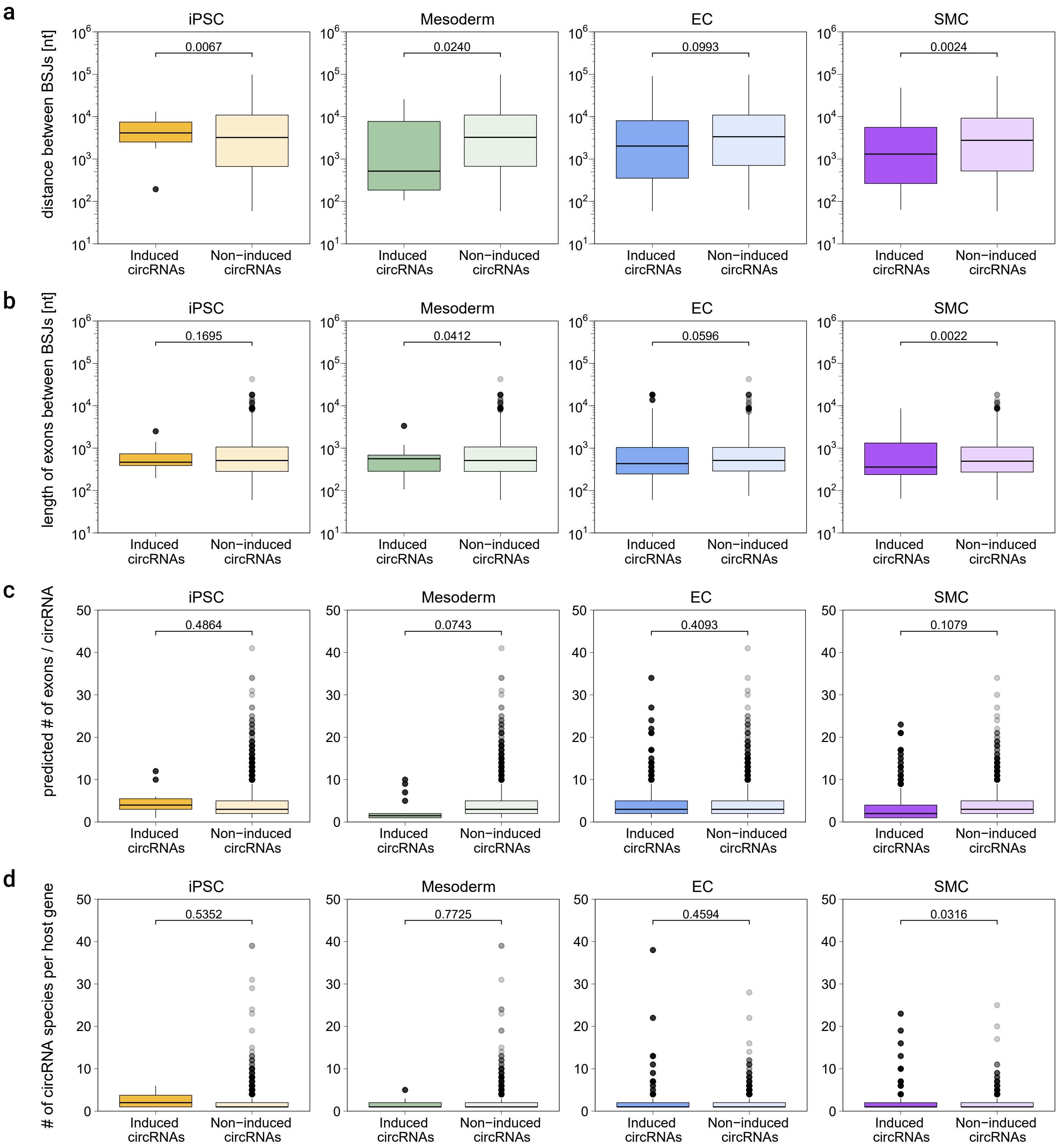
a
b
c
d
iPSC
Mesoderm
EC
SMC
distance between BSJs [nt]
length of exons between BSJs [nt]
predicted # of exons / circRNA
# of circRNA species per host gene
0.0067
0.0240
0.0993
0.0024
0.1695
0.0412
0.0596
0.0022
0.4864
0.0743
0.4093
0.1079
0.5352
0.7725
0.4594
0.0316
Induced circRNAs
Non−induced circRNAs

Fig. S15

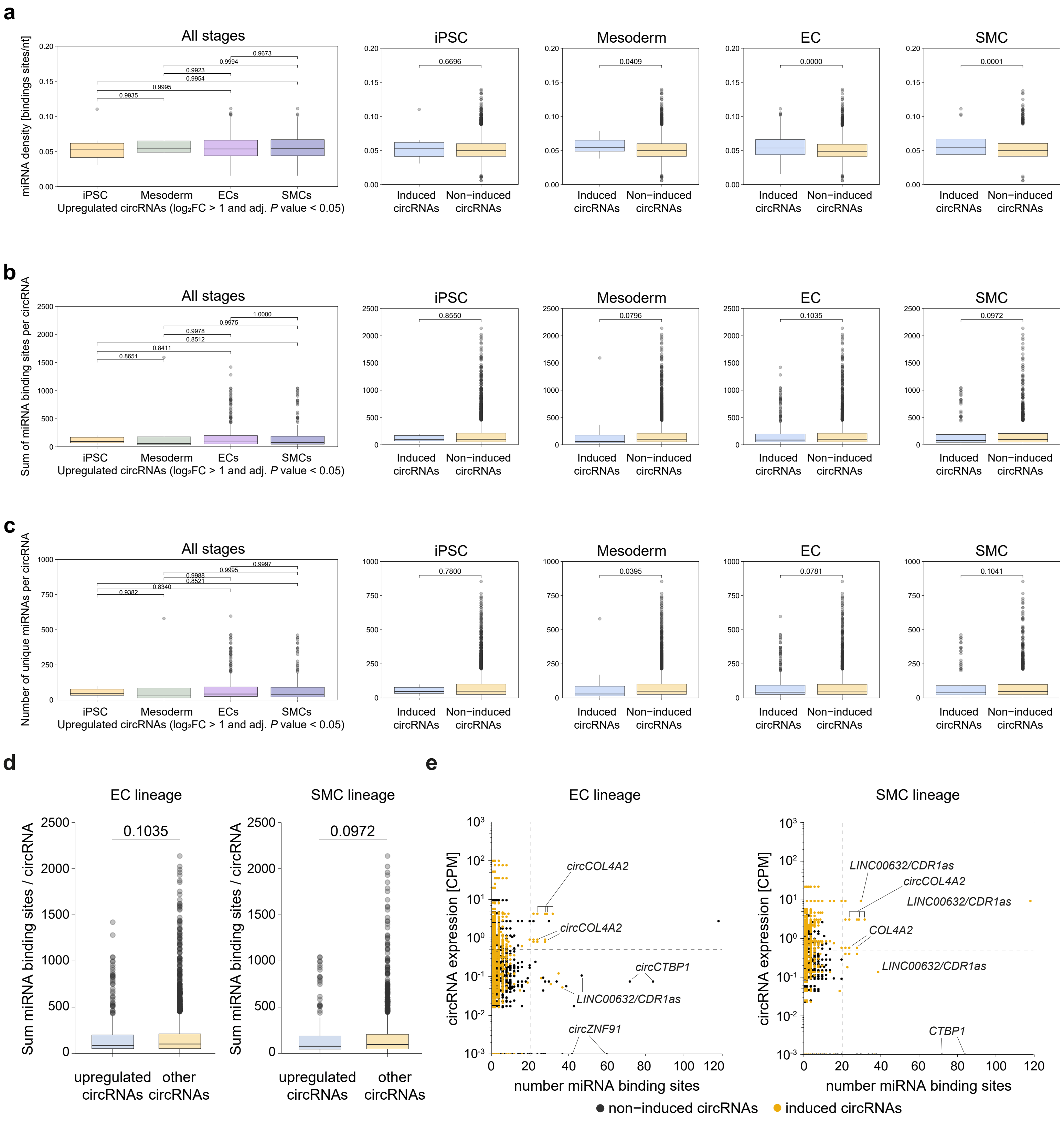
a
All stages
miRNA density [bindings sites/nt]
iPSC
Mesoderm
ECs
SMCs
Upregulated circRNAs (log₂FC > 1 and adj. P value < 0.05)
iPSC
Mesoderm
EC
SMC
Induced circRNAs
Non−induced circRNAs
0.6696
0.0409
0.0000
0.0001
b
Sum of miRNA binding sites per circRNA
0.8550
0.0796
0.1035
0.0972
c
Number of unique miRNAs per circRNA
0.7800
0.0395
0.0781
0.1041
d
EC lineage
SMC lineage
Sum miRNA binding sites / circRNA
upregulated circRNAs
other circRNAs
e
circRNA expression [CPM]
number miRNA binding sites
circCOL4A2
circCTBP1
LINC00632/CDR1as
circZNF91
COL4A2
CTBP1
non−induced circRNAs
induced circRNAs

Fig. S16

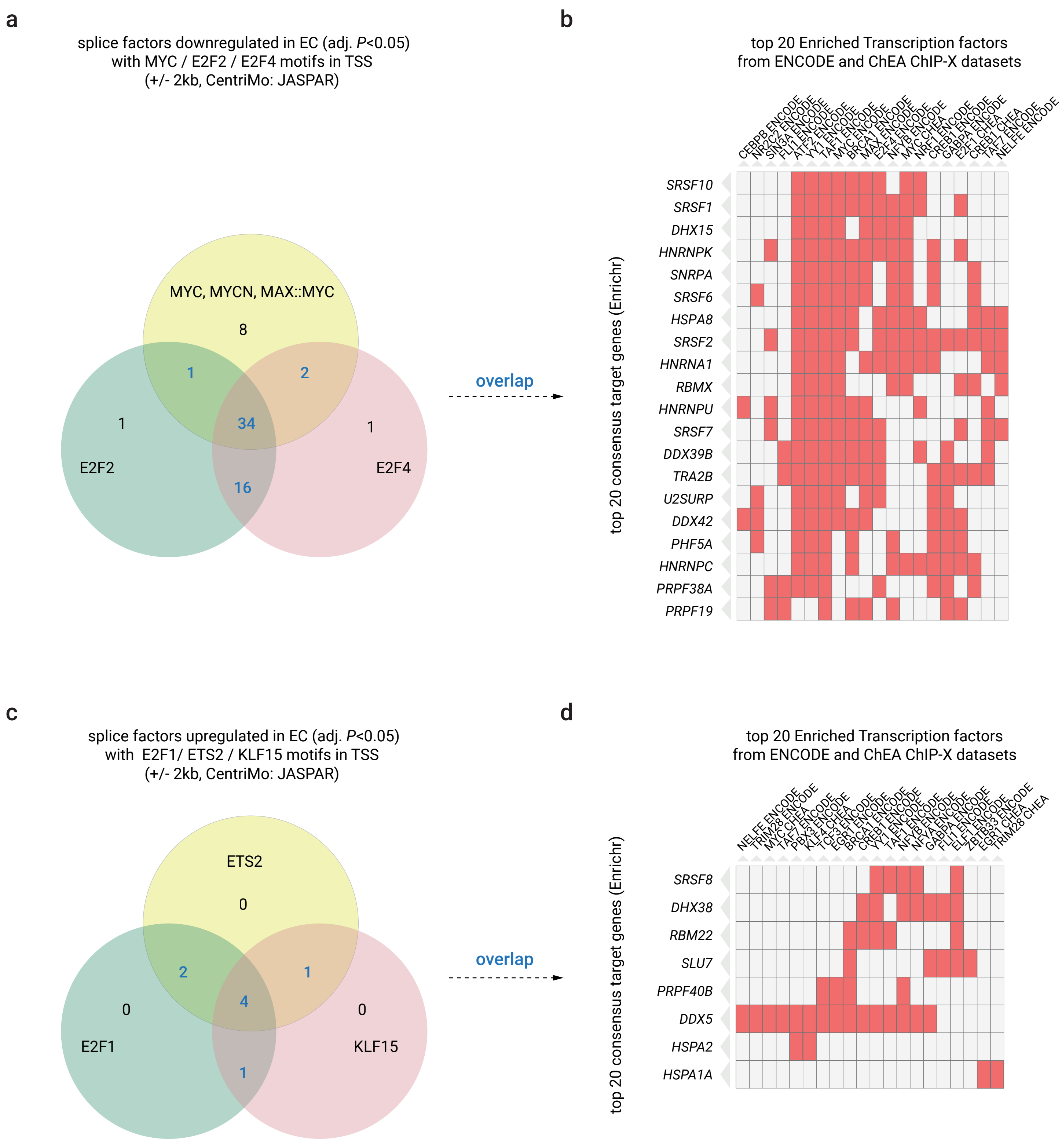
a
splice factors downregulated in EC (adj. P<0.05)
with MYC / E2F2 / E2F4 motifs in TSS
(+/- 2kb, CentriMo: JASPAR)
MYC, MYCN, MAX::MYC
8
1
2
1
34
1
E2F2
E2F4
16
overlap
b
top 20 Enriched Transcription factors
from ENCODE and ChEA ChIP-X datasets
CEBPB ENCODE
NR2C2 ENCODE
SIN3A ENCODE
FLI1 ENCODE
ATF2 ENCODE
YY1 ENCODE
TAF1 ENCODE
MYC ENCODE
BRCA1 ENCODE
MAX ENCODE
E2F4 ENCODE
NFYB ENCODE
MYC CHEA
NRF1 ENCODE
CREB1 ENCODE
GABPA ENCODE
E2F1 CHEA
CREB1 CHEA
TAF7 ENCODE
NELFE ENCODE
top 20 consensus target genes (Enrichr)
SRSF10
SRSF1
DHX15
HNRNPK
SNRPA
SRSF6
HSPA8
SRSF2
HNRNA1
RBMX
HNRNPU
SRSF7
DDX39B
TRA2B
U2SURP
DDX42
PHF5A
HNRNPC
PRPF38A
PRPF19
c
splice factors upregulated in EC (adj. P<0.05)
with E2F1/ ETS2 / KLF15 motifs in TSS
(+/- 2kb, CentriMo: JASPAR)
ETS2
0
2
1
0
4
0
E2F1
KLF15
1
overlap
d
top 20 Enriched Transcription factors
from ENCODE and ChEA ChIP-X datasets
NELFE ENCODE
TRIM28 ENCODE
MYC CHEA
TAF7 ENCODE
PBX3 ENCODE
KLF4 CHEA
TCF3 ENCODE
EGR1 ENCODE
BRCA1 ENCODE
CREB1 ENCODE
YY1 ENCODE
TAF1 ENCODE
NFYB ENCODE
NFYA ENCODE
GABPA ENCODE
ELF1 ENCODE
FLI1 ENCODE
ZBTB33 ENCODE
EGR1 CHEA
TRIM28 CHEA
top 20 consensus target genes (Enrichr)
SRSF8
DHX38
RBM22
SLU7
PRPF40B
DDX5
HSPA2
HSPA1A

Fig. S17

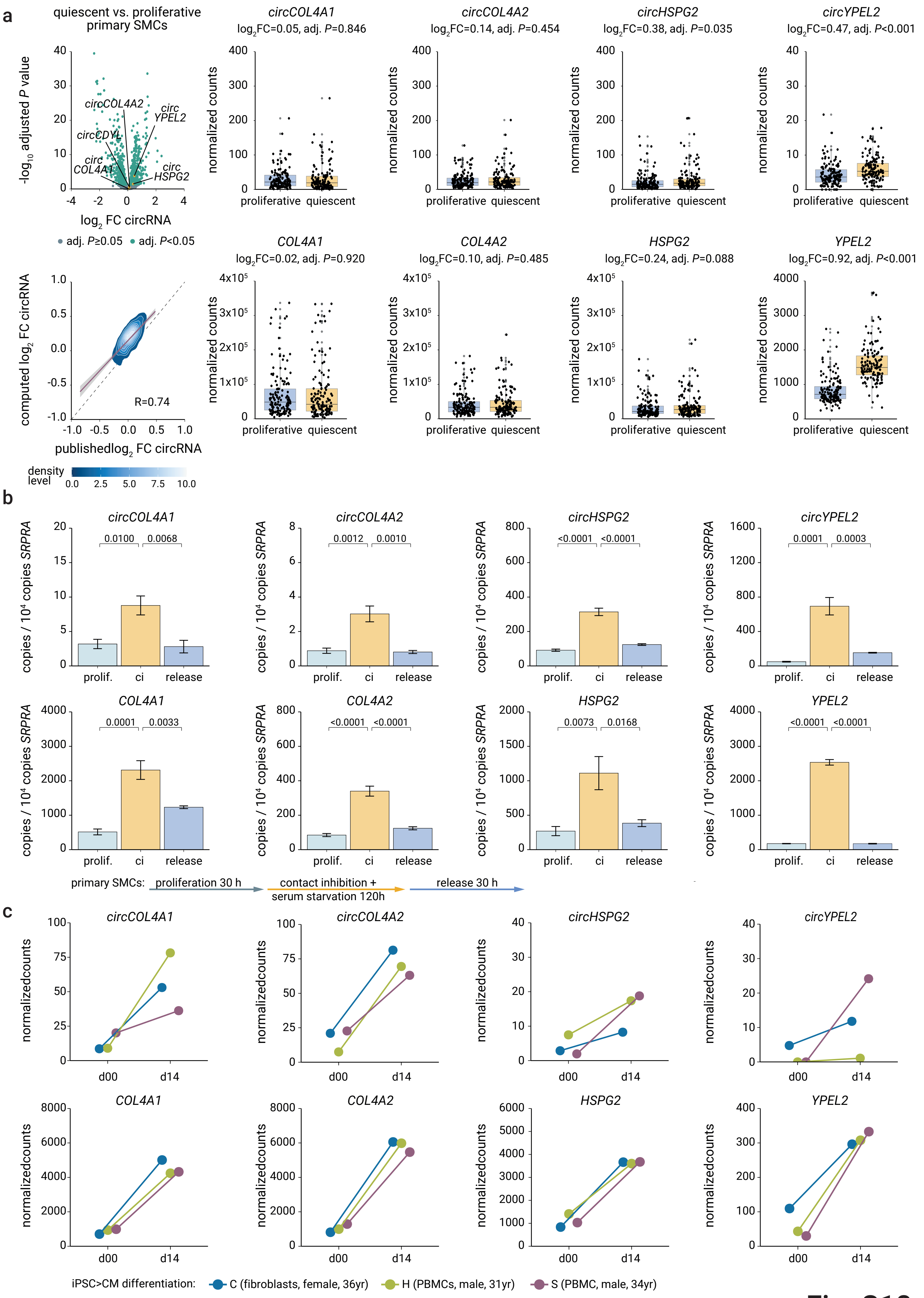

a
quiescent vs. proliferative primary SMCs
-log10 adjusted P value
log2 FC circRNA
circCOL4A2
circYPEL2
circCDYL
circCOL4A1
circHSPG2
adj. P≥0.05
adj. P<0.05
computed log2 FC circRNA
publishedlog2 FC circRNA
R=0.74
density level
circCOL4A1
log2FC=0.05, adj. P=0.846
circCOL4A2
log2FC=0.14, adj. P=0.454
circHSPG2
log2FC=0.38, adj. P=0.035
circYPEL2
log2FC=0.47, adj. P<0.001
COL4A1
log2FC=0.02, adj. P=0.920
COL4A2
log2FC=0.10, adj. P=0.485
HSPG2
log2FC=0.24, adj. P=0.088
YPEL2
log2FC=0.92, adj. P<0.001
normalized counts
proliferative
quiescent
b
copies / 10⁴ copies SRPRA
circCOL4A1
0.0100
0.0068
circCOL4A2
0.0012
0.0010
circHSPG2
<0.0001
<0.0001
circYPEL2
0.0001
0.0003
COL4A1
0.0001
0.0033
COL4A2
<0.0001
<0.0001
HSPG2
0.0073
0.0168
YPEL2
<0.0001
<0.0001
prolif.
ci
release
primary SMCs: proliferation 30 h
contact inhibition + serum starvation 120h
release 30 h
c
normalizedcounts
circCOL4A1
circCOL4A2
circHSPG2
circYPEL2
COL4A1
COL4A2
HSPG2
YPEL2
d00
d14
iPSC>CM differentiation:
C (fibroblasts, female, 36yr)
H (PBMCs, male, 31yr)
S (PBMC, male, 34yr)

Fig. S18

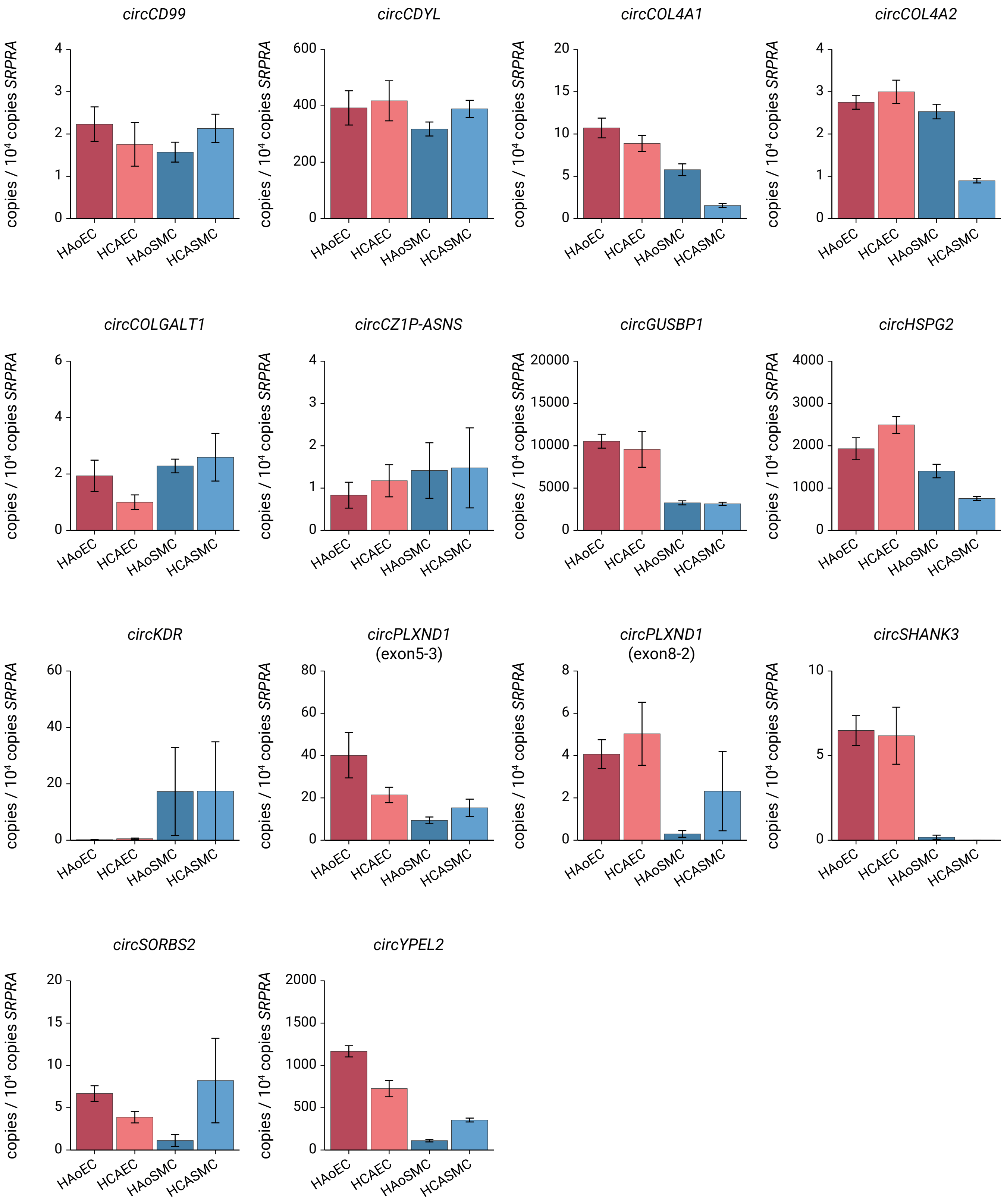

circCD99
circCDYL
circCOL4A1
circCOL4A2
circCOLGALT1
circCZ1P-ASNS
circGUSBP1
circHSPG2
circKDR
circPLXND1 (exon5-3)
circPLXND1 (exon8-2)
circSHANK3
circSORBS2
circYPEL2
copies / 10⁴ copies SRPRA
HAoEC
HCAEC
HAoSMC
HCASMC

Fig. S19

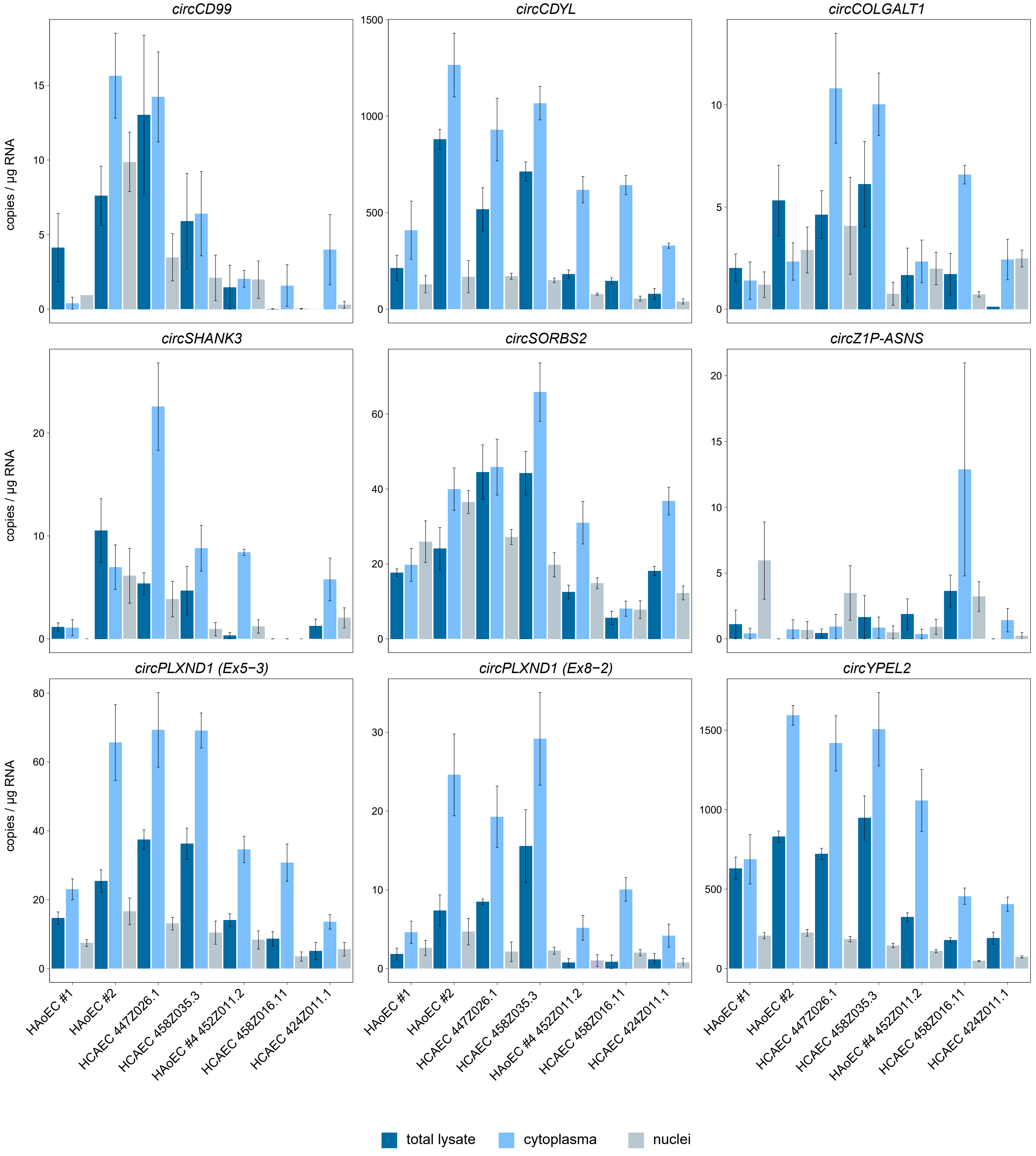
circCD99
circCDYL
circCOLGALT1
circSHANK3
circSORBS2
circZ1P-ASNS
circPLXND1 (Ex5−3)
circPLXND1 (Ex8−2)
circYPEL2
copies / µg RNA
HAoEC #1
HAoEC #2
HCAEC 447Z026.1
HCAEC 458Z035.3
HAoEC #4 452Z011.2
HCAEC 458Z016.11
HCAEC 424Z011.1
total lysate
cytoplasma
nuclei

Fig. S20

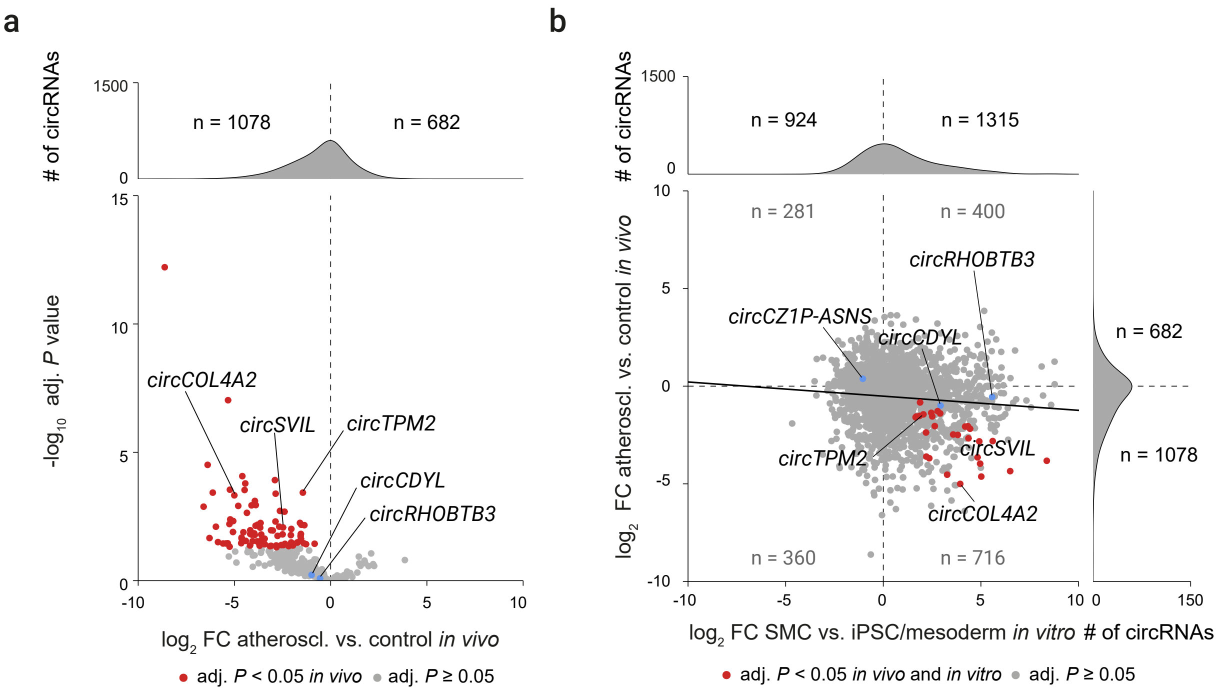
a
b
# of circRNAs
1500
0
n = 1078
n = 682
15
10
5
0
-log10 adj. P value
circCOL4A2
circSVIL
circTPM2
circCDYL
circRHOBTB3
-10
-5
0
5
10
log2 FC atheroscl. vs. control in vivo
adj. P < 0.05 in vivo
adj. P ≥ 0.05
n = 924
n = 1315
n = 281
n = 400
n = 360
n = 716
circRHOBTB3
circCZ1P-ASNS
circCDYL
circTPM2
circSVIL
circCOL4A2
log2 FC atheroscl. vs. control in vivo
log2 FC SMC vs. iPSC/mesoderm in vitro
# of circRNAs
150
n = 682
n = 1078
adj. P < 0.05 in vivo and in vitro
adj. P ≥ 0.05

Fig. S21

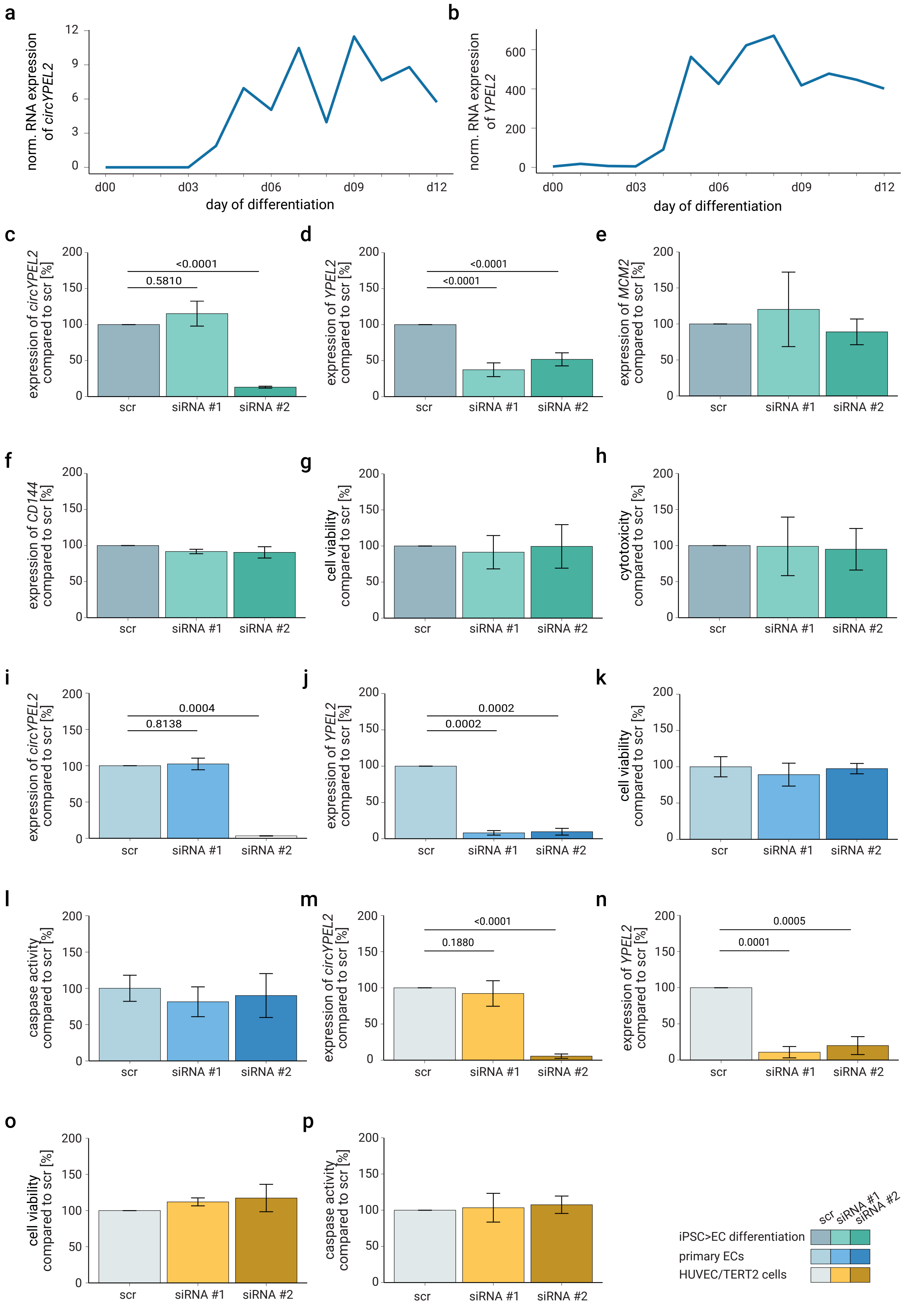
a
norm. RNA expression of circYPEL2
day of differentiation
d00
d03
d06
d09
d12
b
norm. RNA expression of YPEL2
c
expression of circYPEL2 compared to scr [%]
<0.0001
0.5810
scr
siRNA #1
siRNA #2
d
expression of YPEL2 compared to scr [%]
<0.0001
<0.0001
e
expression of MCM2 compared to scr [%]
f
expression of CD144 compared to scr [%]
g
cell viability compared to scr [%]
h
cytotoxicity compared to scr [%]
i
expression of circYPEL2 compared to scr [%]
0.0004
0.8138
j
expression of YPEL2 compared to scr [%]
0.0002
0.0002
k
cell viability compared to scr [%]
l
caspase activity compared to scr [%]
m
expression of circYPEL2 compared to scr [%]
<0.0001
0.1880
n
expression of YPEL2 compared to scr [%]
0.0005
0.0001
o
cell viability compared to scr [%]
p
caspase activity compared to scr [%]
iPSC>EC differentiation
primary ECs
HUVEC/TERT2 cells

Fig. S22

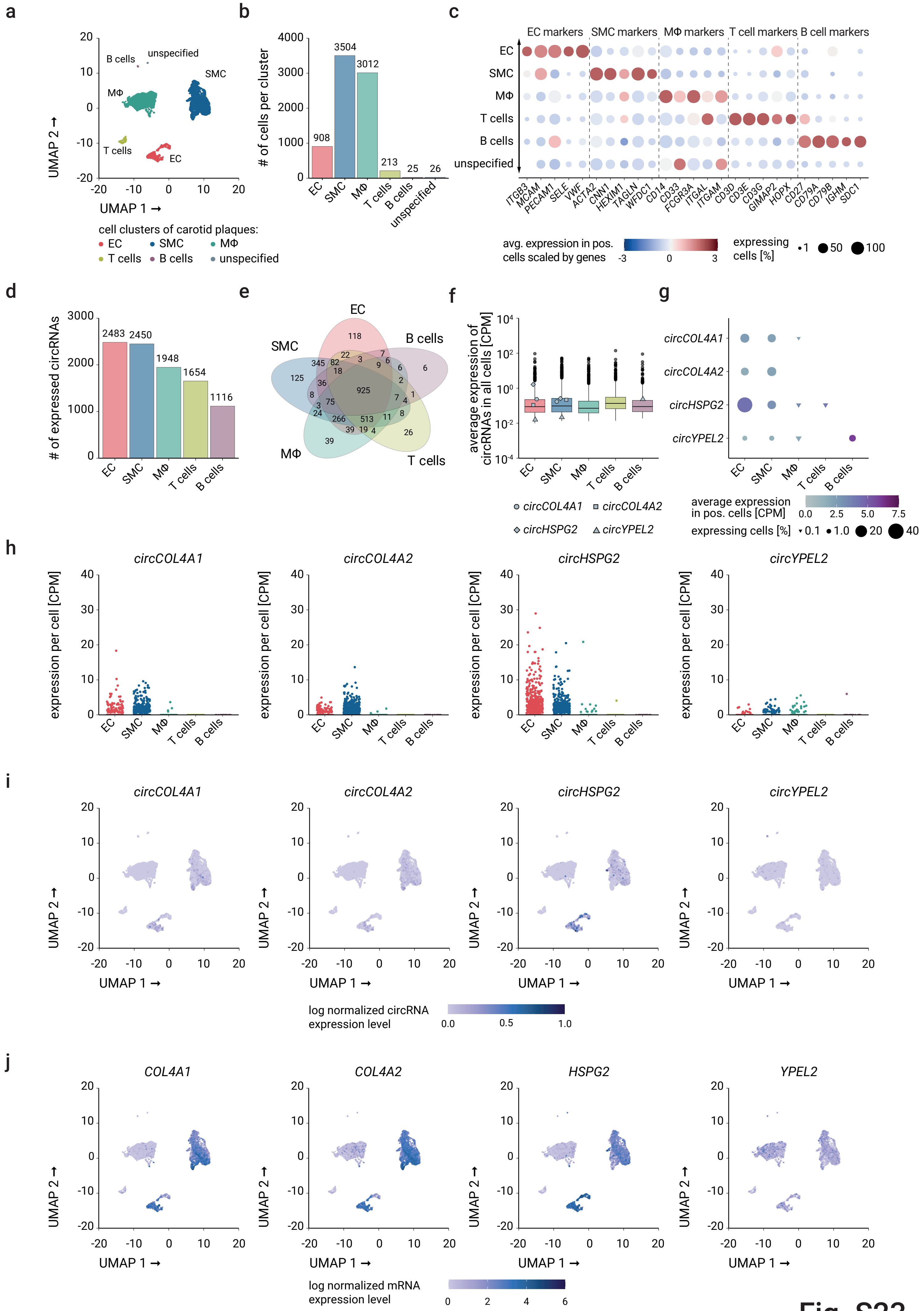

a
b
c
d
e
f
g
h
i
j
cell clusters of carotid plaques:
EC SMC MΦ T cells B cells unspecified
UMAP 1 →
UMAP 2 →
# of cells per cluster
908 3504 3012 213 25 26
EC markers SMC markers MΦ markers T cell markers B cell markers
avg. expression in pos. cells scaled by genes
expressing cells [%]
# of expressed circRNAs
2483 2450 1948 1654 1116
average expression of circRNAs in all cells [CPM]
circCOL4A1 circCOL4A2 circHSPG2 circYPEL2
average expression in pos. cells [CPM]
expressing cells [%]
expression per cell [CPM]
log normalized circRNA expression level
COL4A1 COL4A2 HSPG2 YPEL2
log normalized mRNA expression level

Fig. S23
