# Supplementary material for: Circular RNAs increase during vascular cell differentiation and are biomarkers for vascular disease
Source: Cardiovasc Res. 2025 Feb 4;121(3):405–23. doi: 10.1093/cvr/cvaf013 (PMC12038242; doi:10.1093/cvr/cvaf013)
Supplement: cvaf013_Supplementary_Data [file cvaf013_supplementary_data.zip › Northoff et al - Supplementary information.pdf]

Supplementary information

CircRNAs increase during vascular cell differentiation and are biomarkers for vascular disease

Bernd H. Northoff[1], Andreas Herbst[1], Catharina Wenk[1], Lena Weindl[1], Gabor Gäbel[2], Andre Brezski[3], Kathi Zarnack[3], Alina Küpper[1], Stefanie Dimmeler[4], Alessandra Moretti[5], Karl-Ludwig Laugwitz[5], Stefan Engelhardt[6], Lars Maegdefessel[7], Reinier Boon[4], Stefanie Doppler[8,9], Martina Dreßen[8,9], Harald Lahm[8,9], Rüdiger Lange[8,9,10], Markus Krane[8,9,10,11], Knut Krohn[12], Alexander Kohlmaier[1], Lesca M. Holdt[1]*& Daniel Teupser[1]*

[1] Institute of Laboratory Medicine, University Hospital, LMU Munich, Munich, Germany;

[2] Department of Vascular Medicine, HELIOS Klinikum Krefeld, Krefeld, Germany;

[3] Buchmann Institute for Molecular Life Sciences (BMLS) and Faculty of Biological Sciences, Goethe University Frankfurt, Frankfurt, Germany;

[4] Institute of Cardiovascular Regeneration, Centre of Molecular Medicine, Goethe University, Frankfurt, Germany;

[5] Department of Internal Medicine I, Cardiology, Klinikum rechts der Isar, School of Medicine & Health, Technical University of Munich (TUM), Germany;

[6] Institute of Pharmacology and Toxicology, Technical University of Munich (TUM), Munich, Germany;

[7] Department of Vascular and Endovascular Surgery, Technical University Munich, Germany;

[8] German Heart Center Munich, Department of Cardiovascular Surgery, Technical University Munich;

[9] Institute for Translational Cardiac Surgery (INSURE), German Heart Center Munich, Technical University Munich

[10] DZHK (German Center for Cardiovascular Research) - partner site Munich Heart Alliance, Munich, Germany

[11] Yale School of Medicine, Division of Cardiac Surgery, Department of Surgery, New Haven, CT, USA;

[12] Core Unit DNA Technologies, Medical Faculty, University of Leipzig, Leipzig, Germany.

Table of content

1. Supplementary Materials and Methods
2. List of Supplementary Figures and Tables
3. Legend for Supplementary Figures
4. Legend for Supplementary Tables

1. Supplementary Materials and Methods

1.1. Cell culture

Primary cells

Human aortic endothelial cell lines (452Z031.1, 458Z035.1, and 452Z011.2 from PromoCell, Heidelberg, Germany), as well as human coronary artery endothelial cell lines (424Z011.1, 447Z026.1, 458Z016.1, 458Z035.3 from PromoCell), were cultured in Endothelial Cell Growth Medium MV (PromoCell, Heidelberg, Germany) with Growth Medium MV Supplement (PromoCell, Heidelberg, Germany) and 1 % Pen/Strep.

Human aortic smooth muscle cell (HAoSMC) lines (335663, 369150, and 430741 from Lonza, Basel, Switzerland) were cultured in SmBM Smooth Muscle Cell Basal Medium (Lonza, Heidelberg, Germany), with SmGM-2 SingleQuots Supplement Pack 2 (Lonza, Basel, Switzerland). HAoSMC line PB-354-05a (Pelo Biotech, Martinsried, Germany) was cultured in Smooth Muscle Cell Basal Medium (Cell Applications, San Diego, CA, USA), and HAoSMC line ACBRI 716 (Pelo Biotech, Martinsried, Germany) was cultured in Complete Classic Medium (Pelo Biotech, Martinsried, Germany).

Human coronary artery smooth muscle cell line 452Z013.3 (PromoCell, Heidelberg, Germany) was cultured in Smooth Muscle Cell Growth Medium 2 (PromoCell, Heidelberg, Germany) with Growth Medium 2 SupplementMix (PromoCell, Heidelberg, Germany).

Cell lines

The immortalized cell lines HUVEC-*TERT2 (*Evercyte, Vienna, Austria) and HUVEC-*MYC/ID1/ID2*-expressing cell line (InSCREENex, Braunschweig, Germany, "*eHUVEC-2"* [1]) were cultured in Endothelial Cell Growth Medium (Lonza, Basel, Switzerland) supplemented with Endothelial Cell Growth Medium SingleQuots (Lonza, Basel, Switzerland), 10 % FCS, and 20 µg/ml G418 (Carl Roth, Karlsruhe, Germany).

FACS analysis of cultured cells

FACS was performed with fixed cells using the Foxp3/Transcription Factor Staining Buffer Set (#00-5523-00, eBioscience) on a BD FACSMelody™ Cell Sorter (BD Biosciences), and data were quantified using FlowJo software v10.9.0 (Tree Star Inc.). Cells were stained with FITC Mouse Anti-Human CD31 (Clone WM-59, #555445), PE Mouse anti-Human CD144 (Clone 55-7H1, #560410), PE Mouse Anti-Human CD140b (Clone 28D4, #558821) or isotype controls (BD Pharmingen).

Immunofluorescence

iPSC-derived EC or SMC were seeded onto glass coverslips (#9210140160, Hecht) coated with Geltrex or fibronectin, respectively, fixed with 4 % paraformaldehyde, permeabilized with 0.2 % Triton X-100, and blocked with 10 % goat serum in 0.3% Tween-20 in PBS. Primary antibodies included mouse monoclonal anti-human CD31 (1:100, Clone JC70A, #M0823, Agilent Technologies) and rabbit monoclonal anti-PDGF Receptor β (1:100, 28E1 #3169, CST) and were incubated in blocking solution at 4 °C overnight and subsequently detected with secondary antibodies AF488 Goat anti-Mouse IgG (1:500, A11029, Invitrogen) and AF568 Goat anti-Rabbit IgG (1:500, A11036, Invitrogen) for 1.5 h at room temperature. Nuclei were counterstained with DAPI (1:10,000, #28718-90-3, PanReac AppliChem), and specimens were mounted with Fluoromount-G™ Mounting Medium (#00-4958-02, Invitrogen). Images were acquired on a Leica DM6 B microscope (Leica).

Knockdowns

Linear *YPEL2* knockdown was achieved with siRNA #1 (D-030916-01, Dharmacon), (target sequence: GGACGAGCAUACCUCUUUA) and siRNA #2 (D-030916-18, Dharmacon), (target sequence: GUGAAGAUGACAAGAUCGA). *circYPEL2* was knocked down with siRNA #2 (D-030916-18, Dharmacon) (target sequence: GUGAAGAUGACAAGAUCGA). Silencer® Select Negative Control siRNA (#4390843, Ambion) was used as a control. siRNAs at a final concentration of 10 nM were delivered into HUVEC-TERT2 cells or primary EC with Lipofectamine™ RNAiMAX (#13778075, Invitrogen) in serum-free medium. 4.5 h after lipofection, fresh whole medium was added.

siRNA-mediated silencing in C6 iPSC-derived EC was performed with siRNA oligos at a final concentration of 100 nM by electroporation using a 4D-Nucleofector® (Lonza Bioscience, program P5, pulse CA-167).

Analyses were performed 48 and 72 hours after lipofection/electroporation for RNA analysis and cell assays, respectively.

Functional cellular experiments

7500 HUVEC-TERT2 cells were seeded per 96-well, pre-coated with 0.1 % gelatine (96-well plate #136101, Thermo Scientific). The next day cells were lipofected with siRNAs as described above. 48 hours post-lipofection CellTiter-Glo Luminescent Cell Viability Assay (#G7571; Promega) and Caspase-Glo 3/7 Assay Systems (#G8091 Promega) were performed according to manufacturer's protocols. Luminescence signals were measured with a SpectraMax Paradigm Multi-Mode Microplate Reader (Molecular Devices).

For C6 iPSC-derived EC, 5000 cells were seeded per 96-well pre-coated with fibronectin (96-well plates #136101, Thermo Scientific, or #3935, Corning). 72 hours post-electroporation of siRNAs the CellTiter-Glo Luminescent Cell Viability Assay (#G7571; Promega) or MultiTox-Fluor Multiplex Cytotoxicity Assay (#G9200, Promega) were performed.

Nuclear, cytoplasmic and whole-cell protein extracts

Nuclear and cytoplasmic fractions for Western blotting were prepared using the PARIS Kit (Ambion, Thermo Fisher Scientific, Waltham, MA, USA) according to the manufacturer's instructions. 24 Mio. primary human EC and SMC, respectively, were transfected with either circRNAs or scrambled RNA using Nucleofector technology (Lonza, Basel, Switzerland).

1.2. High throughput RNA sequencing for combined linear and circular RNA quantification

Pre-processing of raw reads

Paired-reads were pre-processed to trim sequences of adapter (-a AGATCGGAAGAGCACACGTCTGAACTCCAGTCA -A AGATCGGAAGAGCGTCGTGTAGGGAAAGAGTGT) and to filter for low-quality bases (--nextseq-trim=20) and a minimum length of reads (--minimum-length 20) using cutadapt software version 2.5. Trimming and filtering were proven using FastQC software version 0.11.8 and MultiQC software version 1.7.

Mapping of RNase R-treated samples

Segemehl algorithm was forced to map paired-end reads using high accuracy (--accuracy 95) and enabled to detect spliced reads (--splits). The segemehl's *testrealign* tool was used to identify splice sites of mapped reads. STAR mapped sequences reads uniquely (-outFilterMultimapNmax 1) with a maximum of 2 of mismatches per pair (--outFilterMismatchNmax 2) to the reference genome and was enabled to detect of chimeric alignments --chimSegmentMin 15 --chimScoreMin 15 --chimScoreSeparation 10 --chimJunctionOverhangMin 15). Bowtie 2 was constrained to map reads sensitively (--very-sensitive --score-min=C,-15,0) and unmapped reads were saved.

Single-cell RNA sequencing (scRNA-seq) analysis

Expression levels of annotated genes were taken from GSE260657 [2]. For circRNA quantification, fastq files were obtained from the NCBI Sequence Read Archive (accession id: PRJNA1082625). Mapping and quantification of circRNAs was performed as described in the methods section above.

Raw counts of annotated genes were analysed using the R package Seurat [3]. Cells expressing less than 300 features and genes detected in less than three cells were excluded. In addition, cells with ≤ 50,000 or > 750,000 total reads or a relative content of mitochondrial or ERCC genes ≥ 10% were excluded. Counts were normalized using the LogNormalize function and multiplied by a scaling factor of 10,000. Principal component analysis was performed using 2,000 most variable features, followed by Uniform Manifold Approximation and Projection (UMAP) dimensional reduction. Nearest-neighbor graph construction was used for clustering. Clusters were annotated based on the differentially expressed genes and annotation from PanglaoDB [4].

For circRNA analysis, circRNAs that were robustly expressed during iPSC differentiation and had ≥ 2 BSJ reads in ≥ 3 cells of one cell type were considered. Count matrices of the pre-filtered genes and circRNAs were merged and analysed with Seurat. Normalization and feature selection were performed as before. The UMAP structure of annotated genes was projected onto the merged data set.

DNA motif enrichment analysis

Enrichment of ChIP peaks among regions of interest was performed by chip-atlas, with a threshold for MACS2 (-10*Log10 [MACS2 Q value]) at Q value < 1E-05. Retrieved experiment types were either "ChIP: TF and others (15155)" or "ChIP: Histones (16612)". Enrichr[5] was used to explore splice factor genes with given CentriMo motifs for enrichment among consensus targets of transcription factors from ENCODE and ChEA ChIP-X datasets. Visualization as clusterograms shows top 20 transcription factors and top 20 input genes after clustering by a combined score (log P value multiplied by z score of the deviation from expected rank)[5].

Cumulative correlation analysis

Correlation coefficients from Spearman correlation of DESeq2-normalized counts of circRNAs and their cognate host mRNA or of a pair of circRNAs (from the group of circRNAs with increased expression during EC differentiation from iPSC) were calculated and plotted as a histogram showing binned distribution.

2. List of Supplementary Figures and Tables:

1 PDF file combining Supplementary Figures S1-S23.pdf

1 EXCEL files of Supplementary Tables S1, S2, S3, S4, S5, S6, S7, S8, S9, S10, S11, S12, S13, S14

3. Legend for supplementary figures

Suppl. Fig S1, accompanying Figure 1

Homogeneity of EC and SMC cell fates assessed by FACS.

Percentage of CD31 (PECAM1)+, CD144 (VE-Cadherin)+, CD140b (PDGFRB)+ positive cells during differentiation into EC (a) or SMC (b) compared to isotype control.

Suppl. Fig S2, accompanying Figure 1

Homogeneity of EC and SMC cell fates assessed by immunofluorescence stainings.

Immunofluorescence stainings of CD31 (PECAM1)+, and CD140b (PDGFRB)+ in EC (a,b) or SMC (c,d) differentiation trajectories from iPSC.

Suppl. Fig S3, accompanying Figure 1

Brighfield images of EC and SMC differentiation trajectories.

Brightfield images of unfixed iPSC, mesoderm progenitors, and maturing EC (a) or SMC (b) in a 12-day long differentiation path. See Figure 1a for description of growth media and cell culture procedures.

Suppl. Fig S4, accompanying Figure 1

Description of RNAseq workflow, quality controls, and general circRNA classification.

(a) Overview of RNAseq samples and experimental design. A total of 160 separate RNAseq libraries were sequenced from 2 separate differentiations of human iPSC to either EC or SMC. Data in the main figures are from biological replicate #1, with 4 samples per day (d1-12) unless otherwise noted. These represent 4 separately grown cell culture dishes, but they are formally technical replicates because they were differentiated in parallel. Selected days were sequenced in another round of differentiation (d0, d3, d6, d9, d12), which are true biological replicates. Separate qRT-PCRs were performed on two separate samples of iPSC lines from different donors (MRIi003-A, MRIi001-A). (b) RNA quality control from cellular samples prior to RNAseq. Shown spectrophotmetric absorbance ratios at indicate wavelengths (nanometer) (left) and Bioanalyzer RNA integrity numbers (RIN) (right) as means and SEM. (c) RNA-sequencing depths of total RNA samples and RNase R-treated RNA samples (+RNase R) for all 160 samples are shown as mean and SEM for each sample. (d-e) RNA quality control by spectrophotometry or based on number of sequenced reads from RNAseq of aortic tissue samples ((control (CTR) and atherosclerotic (CAD)) (d) and of blood peripheral mononuclear cells (PBMC) (e). See Fig. 5 for tissue-level and blood-level analysis. Shown are mean and SEM.

Suppl. Fig S5, accompanying Figure 1

Expression of cell differentiation markers.

(a) mRNA levels of published markers for stem cells, mesodermal progenitor cells, EC, and various EC subtypes in the iPSC-mesoderm-EC differentiation trajectory based on RNAseq datasets from biological replicate #1. Mean +/- SEM of expression levels normalized to 10^6 copies *HUWE1* are given. Statistical testing was performed using ANOVA corrected for multiple testing with Tukey's post hoc test. (b) Non-supervised hierarchical clustering at gene-level RNAseq of EC and SMC differentiation trajectories (biological replicate #1), with a set of 7-14 linear mRNA markers expected to distinguish the 4 cell fates based on evidence from the literature. These markers cluster iPSC and the 12 days of *in vitro* differentiation in their correct temporal sequence (iPSC, fetal lateral-plate mesoderm-like, early PDGF-BB- induced vascular SMC lineage, and nonhemogenic EC lineage) and more generally separate SMC from EC (Fig. 1a for reference).

Suppl. Fig S6, accompanying Figure 1

Comparison of linear and circRNA datasets and genomic context of backsplicing.

(a) PCA of RNAseq confirms the similarities of linear and circRNA datasets in distinguishing cell type and cell-differentiation phase in biological replicates #1 and #2. (b) Genomic context of backsplicing. Most circRNAs from iPSC-derived EC and iPSC-derived SMC differentiation (total) derive from protein-coding genes. (c) These circRNAs mainly represent backsplicing between exons within gene bodies. SD (splice donor), SA (splice acceptor), UTR (untranslated region).

Suppl. Fig S7, accompanying Figure 1

Validation of circularity of circRNA candidates.

Validation of circularity of circRNA candidates identified by RNAseq (from Fig. 1). circRNAs are resistant to exonucleolytic digestion by RNase R, whereas linear mRNA of the *SRPRA* gene is degraded under these conditions. Expression levels are normalized to 1 µg RNA input and are reported as mean +/- SEM.

Suppl. Fig S8, accompanying Figure 2

circRNA signatures for each state in the vascular differentiation trajectory.

Heatmaps showing non-hierarchical clustering of normalized circRNA BSJ reads from RNAseq during differentiation from iPSC to SMC or EC. The temporal trajectory is schematized by shades of grey from light (d0, iPSC) to dark (d12, differentiated). Each column represents an independent RNAseq sample. Each row corresponds to a different circRNA with an identifier given on the right. (a) Signature of circRNAs (boxed) unique

to the iPSC state at d0 (iPSC>EC differentiation). (b) Signature of circRNAs (boxed) unique to mesoderm state (iPSC>EC differentiation). (c) Signature of circRNAs (boxed) unique to the differentiated SMC state from the SMC-differentiation trajectory. (d) Signature of circRNAs (boxed) unique to the differentiated EC state from the EC-differentiation trajectory. Coordinates for circRNAs from the circRNA signatures boxed in (a-d) are shown on the right in the same order (top to bottom) as in the heatmap.

Suppl. Fig S9, accompanying Figure 2

Validation of circRNA trajectories from RNAseq by qRT-PCR and levels in primary EC.

The expression levels of *circCD99*, *circCDYL*, *circCOL4A2*, *circCOLGALT1*, *circCZ1P-ASNS*, *circGUSBP1*, *circKDR* and *circPLXND1* were validated (a-c). For these circRNAs, trajectories of circRNA abundance from DESeq2-normalized counts from RNAseq (a) were validated by qRT-PCR in the iPSC line ISFi001-A and primary endothelial cells (b). Note the close agreement of temporal changes in the two data sets. Further, note that circRNA levels in iPSC-derived EC are comparable to levels in independent primary EC. Mean +/- SEM of expression levels normalized to 10^6 copies *HUWE1* are given. Statistical tests were performed using ANOVA and corrected for multiple testing with Tukey's post hoc test. (c) The temporal trajectory of each of the 8 circRNAs was recapitulated by differentiating two additional iPSC lines from different donors to EC *in vitro* (MRIi003-A, MRIi001-A). Note the close agreement in circRNA abundance measured by qRT-PCR compared with the original iPSC cell line ISFi001-A. Expression levels are normalized to 10^6 copies *HUWE1* and are reported as mean +/- SEM.

Suppl. Fig S10, accompanying Figure 2

Validation of circRNA trajectories from RNAseq by qRT-PCR and levels in primary EC.

The expression levels of *circPLXND1*, *circRHOBTB3*, *circSHANK3*, *circSORBS2*, *circSPARC*, *circSVIL*, *circTPM2* and *circYPEL2* were validated (a-c). For these circRNAs, trajectories of circRNA abundance from DESeq2-normalized counts from RNAseq (a) were validated by qRT-PCR in the iPS cell line ISFi001-A and in primary endothelial cells (b). Note the close agreement of temporal changes in the two data sets. Further, note that circRNA levels in iPSC-derived EC are comparable to levels in independent primary EC. Mean +/- SEM of expression levels normalized per 10^6 copies *HUWE1* are given. Statistical tests were performed using ANOVA, corrected for multiple testing with Tukey's post hoc test. (c) The temporal trajectory of each of the 8 circRNAs was recapitulated by differentiating two additional iPSC lines from different donors to EC *in vitro* (MRIi003-A, MRIi001-A). Note the close agreement in circRNA abundance measured by qRT-PCR compared with the original iPSC cell line ISFi001-A. Expression levels are normalized per 10^6 copies *HUWE1* and are reported as mean +/- SEM.

Suppl. Fig S11, accompanying figure 2

Validation of circRNA trajectories from RNAseq by qRT-PCR and levels in primary SMC.

The expression levels of *circCDYL*, circCOL4A2, *circCOL5A2*, *circCOL6A2*, *circRHOBTB3*, *circSPARC*, *circSVIL*, and *circTPM2* were validated (a-c). For these circRNAs, trajectories of circRNA abundance from DESeq2-normalized counts from RNAseq (a) were validated by qRT-PCR in the iPS cell line ISFi001-A and in primary smooth muscle cells (b). Note the close agreement of temporal changes in the two data sets. Further, note that circRNA levels in iPSC-derived SMC are comparable to levels in independent primary SMC. Mean +/- SEM of expression levels normalized per 10^6 copies *HUWE1* are given. Statistical tests were performed using ANOVA and corrected for multiple testing with Tukey's post hoc test. (c) The temporal trajectory of each of the 8 circRNAs was recapitulated by differentiating two additional iPSC lines from different donors to SMC *in vitro* (MRIi003-A, MRIi001-A). Note the close agreement in circRNA abundance measured by qRT-PCR compared with the original iPSC cell line ISFi001-A. Expression levels are normalized per 10^6 copies *HUWE1* and are reported as mean +/- SEM.

Suppl. Fig S12, accompanying Figure 3

Circular and linear RNA expression during differentiation at different thresholds for minimal detection.

(a-b) Waterfall plots showing expression levels and number of expressed circRNAs for iPSC>EC differentiation (a) and iPSC>SMC differentiation (b). Left: Median expression levels of each circRNA, FSJ, gene-level host mRNAs, and gene-level host mRNAs per cell stage (yellow: iPSC at d0; green: mesoderm at d2, d3; blue: differentiated cells at d6, d7, d10, d12) were ranked according to expression. Right: Mean +/- SEM for the numbers of expressed circRNAs, FSJs, host genes, and gene-level host mRNAs are given. Statistical tests were performed using the Wald test based on a generalized linear regression analysis using Poisson distribution. (c) The numbers of circRNAs at each stage of differentiation are given in the left panel. In the right panel, for circRNAs expressed in differentiated EC or SMC, the numbers in the less differentiated phases are given. (d) For circRNAs expressed in differentiated EC or SMC, the numbers of circRNAs in the less differentiated phases are given at the absolute baseline detection limit (when a single RNAseq read was quantified on any day of iPSC or mesoderm phase, this circRNA was taken into account), indicating low-level spurious circRNA production predating a future differentiation decision.

Suppl. Fig S13, accompanying Figure 3

CircRNA changes during contact inhibition of HUVEC.

(a) Scheme describing the experimental design for deriving RNA samples after contact inhibition or after release from contact inhibition in an immortalized HUVEC line (HUVEC-*TERT2*). (b) Cell confluence measurements using IncuCyte Zoom live imaging as a proxy for proliferation rate. The example shows cells that were first contact inhibited and then released. (c) qRT-PCR of two mesenchymal mRNA markers (*TAGLN*, *CNN1*) and *CD144* as EC

marker, ruling out de-differentiation of HUVEC during contact inhibition. (d) qRT-PCRs of exemplary circRNAs and their cognate linear host mRNAs during contact inhibition and release. (e) qRT-PCRs showing CLRs for *YPEL2* , *CDYL*, *SHANK3* and *PLXND1* circRNAs during contact inhibition and release. Absolute expression levels are normalized to 10^6 copies *SRPRA*. Data show mean +/- SEM of n=3 cell culture replicates with qRT-PCR measurements in quadruplicates (n=4). *P* values in bar graphs indicate significance from ANOVA corrected for multiple testing using Tukey's post hoc test.

Suppl. Fig S14, accompanying Figure 4

Regulation of vascular splice factors.

(a) mRNA levels from RNAseq for transcription factors showing enriched binding to promoters of circRNA host gene promoters (from Fig. 4a). (b) Enrichment analysis by chip-atlas from endothelial ChIPseq datasets deposited at NCBI SRA regarding histone modifications. Shown is the association with genomic regions hosting circRNAs that increased in EC ($log_2FC > 1$, adj. *P* value < 0.05) compared to host loci for unchanged circRNAs (see Fig. 4a). (c) Histogram showing binned distributions of Spearman correlation coefficients (see methods) between pairs of circRNA and host mRNA (blue), or pairs of circRNAs (red), in both cases for circRNAs with increased expression during EC differentiation. Note the positive rightward shift in the mean of circRNA-circRNA correlations (red) compared to the mean of circRNA-host mRNA correlations (blue). (d) mRNA levels from RNAseq for MYC family member *MYCN* and additional growth-regulatory MYC target genes. (e) mRNA levels from RNAseq for quiescence-activator *FOXO1*, and levels of three cell cycle-regulated genes, *PCNA*, *Cyclin B1*, and *Cyclin E2*. Statistical significance was tested using Student's t-test. *** represents *P* value < 0.001.

Suppl. Fig S15, accompanying Figure 4

Genomic features of circularization events during EC and SMC differentiation.

Comparison of physical features of circRNA splicing in different groups of circRNAs during iPSC differentiation to EC or SMC. (a) Changes in the distance between backsplice donor and backsplice acceptor exon. (b) Length of any individual exon within the coordinates of the backsplice event. (c) Total number of all host gene exons within the genomic range of the circRNA coordinates. (d) Number of distinct circRNA backsplice events per host gene ID. Note that differentiation-linked circRNAs tended to be from splice sites closer to each other than unchanged circRNAs and had smaller exons, but the effect sizes were small. Statistical comparisons were performed using the two-tailed Student's t-test.

Suppl. Fig S16, accompanying Figure 4

Analysis of microRNA binding sites in EC- and SMC-associated circRNAs.

(a) MicroRNA binding site density: Number of microRNA binding sites normalized to circRNA length. (b) Total sum of all different microRNA binding sites per circRNA. (c) Number of unique microRNA binding sites per circRNA. Statistical testing was performed using Mann–Whitney U test or ANOVA corrected for multiple testing using Tukey's post hoc test. (d) Analysis of microRNA binding sites in circRNAs enriched in EC or SMC differentiation *in vitro* ($\log_2$FC > 1, adj. *P* value < 0.05). The sums of miRNA binding sites per circRNA are given for each group and compared by Student's t-test. (e) Correlation between the number of microRNA binding sites per circRNA and the expression level of this circRNA during the differentiation trajectory to EC or SMC. circRNAs enriched during differentiation are labelled in yellow.

Suppl. Fig S17, accompanying Figure 4

Analysis of combinations of transcription factor binding motifs in promoters of vascular splicing factors.

(a) Venn diagram showing splicing factors downregulated during differentiation of iPSC to EC sharing motifs for E2F2 and/or E2F4 and/or the MYC protein family from CentriMo analysis using the JASPAR database (from Fig. 4d). (b) Clusterogram from Enrichr analysis showing the ENCODE and ChEA ChIP-X peak associations of transcription factors with the promoter of splicing factors from the overlaps (blue) in (a). The top 20 splicing factors and the top 20 associated transcription factors are shown after ranking by *P* value and z-score, see methods. (c) Venn diagram showing splicing factors upregulated in EC sharing motifs for E2F1 and/or known EC-fate-controlling transcription factors (ETS2 and/or KLF15 from Fig 4d). (d) Clusterogram from Enrichr analysis as in (b) for the overlaps (blue) from (c).

Suppl. Fig S18, accompanying Figure 4

circRNA levels in serum-starved primary SMC and during cardiomyocyte differentiation from iPSC

(a) Volcano plot (top left) of circRNAs quantified by RNAseq in serum-depleted primary SMC lines (n=139) and corresponding proliferative SMCs (n=145) from 151 multiethnic donors. circRNAs were quantified *de novo* using the described algorithm in total RNAseq data from GSE193817 [6]. The correlation plot (bottom left) shows the overall congruence when comparing differential expression of *de novo* quantified circRNAs with differential expression of circRNAs from external publication in which a different algorithm was used[6]. The bar graphs (right) show level of expression of signature circRNAs in the quiescent and proliferative states. Each dot represents a unique SMC line from a distinct individual. (b) Expression levels of top differentiation-associated circRNAs quantified by qRT-PCR in primary human aortic SMCs during regular proliferative culture (grey), after 5 days in contact-inhibition (yellow), and 30 hours after release and re-seeding of previously contact-inhibited cells into proliferative conditions (blue). Mean +/- SEM of expression levels are normalized to 10^4 copies *SRPRA*. (c) circRNA profiling during differentiation of cardiomyocytes from iPSCs at day 0 and day 14. Expression levels of circRNAs (top) and their linear host mRNAs (bottom) at day 0 and day 14 of iPSC differentiation [7] determined by total

RNAseq analysis with the described quantification algorithm. Different colors show results from 3 genetically different donors (n=1 each).

Suppl. Fig S19, accompanying Figure 5

circRNA steady-state levels in vascular ECs and SMCs from different vascular beds.

Expression levels of top differentiation-associated circRNAs in primary human aortic endothelial cells (HAoEC), primary human coronary artery endothelial cells (HCAEC), primary human aortic smooth muscle cells (HAoSMC) and primary human coronary artery smooth muscle cells (HCASMC) quantified by qRT-PCR. Mean +/- SEM of expression levels are normalized to 10^4 copies *SRPRA*.

Suppl. Fig S20, accompanying Figure 5

Subcellular distribution of circRNAs in nucleus and cytoplasm in EC and SMC cell lines.

qRT-PCR expression analysis of the subcellular distribution of top differentiation-associated circRNAs in nuclear and cytoplasmic fractions of primary human endothelial cells. Total cell extract serve as reference. Note that most circRNAs are predominantly cytoplasmic but that every circRNA is at least partially present in the nucleus. Mean and SEM of expression levels normalized to µg input RNA are given.

Suppl. Fig S21, accompanying Figure 5

SMC differentiation-associated circRNAs in atherosclerotic tissue samples from patients.

(a) Volcano plot of changes of SMC-linked circRNAs in bulk tissue RNAseq analysis comparing atherosclerotic aorta (atherosclerosis) and healthy reference aortic tissue (atherosclerosis-free mammarian aorta, control) from human patients of the MyTi cohort (n=54 patients). CircRNAs highlighted in red reached significance ($log_2FC > 1$, adj. *P* value < 0.05). (b) Correlation of RNAseq-based circRNA levels in MyTi tissue profiling and *in vitro* SMC differentiation. Red-labelled circRNAs reached significance in both data sets ($log_2FC > 1$, adj. *P* value < 0.05). These data from SMC accompany data from EC in the main figure 5.

Suppl. Fig S22, accompanying Figure 5

Knockdown of *circYPEL2* or linear*YPEL2* in EC.

(a-h) Knockdown in the course of EC differentiation from iPSC. (a) Expression levels of *circYPEL2* during the course of EC differentiation from iPSC. (b) Abundance of the major linear *YPEL2* mRNA transcript of EC differentiation from iPSC. (c-d) Specificity of *circYPEL2* knockdown with siRNA#2 (see Suppl. Methods). (e-h) Effects of knockdown on MCM2 mRNA levels (a marker as proxy for proliferation state), on EC differentiation

marker CD144 mRNA, and on viability and cell death. (i-l) Knockdown results from primary EC, and (m-p) knockdown results from HUVEC-TERT2 cell line. Graphs show mean +/- SD. P values shown within bar graphs indicate significance from ANOVA corrected for multiple testing using Dunnett's *post hoc* test. N= 3 biological replicates with n ≥4 technical quadruplicates for all qRT-PCRs, and for viability and apoptosis assays in HUVEC–TERT2. N=2 biological replicates and n=4 technical replicates for viability and apoptosis assay in primary EC. N=2 biological replicates and n=4 technical replicates for viability and cytotoxicity assay in iPSC-derived EC.

Suppl. Fig S23, accompanying Figure 5

Single-cell RNA sequencing to determine the cellular origin of circRNAs in human atherosclerotic carotid plaques.

(a) Dissociated cells of human carotid plaques from 15 individuals were clustered into ECs (red), SMCs (blue), macrophages (MΦs, green), T cells (yellow) and B cells (purple) based on linear RNA expression patterns (from GSE260657) and visualized with Uniform Manifold Approximation and Projection (UMAP). (b) The bar chart shows the number of cells per cluster from (a). (c) Average expression levels of the annotated cell marking linear mRNAs (PangloaDB) are shown per cluster. The colour of the dots indicates the average expression level and the dot size indicates the percentage of expressing cells, considering only cells with detectable expression. (d) Number of circRNAs detected per cluster, expressed in at least 1% of cells per cluster. (e) Detected circRNAs are expressed in different cell types. (f) Boxplot shows the average expression of circRNAs in all cells. All cells per cluster were considered to calculate the average CPM values. The expression levels of the four signature circRNAs (*circCOL4A1*, *circCOL4A2*, *circHSPG2*, *circYPEL2*) are highlighted with symbols. (g) Dotplot shows the expression levels of circRNAs in each cluster. The colour of the dots represents the average expression indicated as CPM in the expressing cells. The dot size reflects the ratio of expressing cells. circRNAs expressed in less than 1% of the cells in the cluster are shown as a triangle. See panel (h) for interpretation of signals. (h) Expression levels of circRNAs per single cell, as shown by CPM values with clusters colour-coded. (i) Quantified BSJ reads of circRNAs per single cell visualized using the UMAP plot, where blue indicates high circRNA expression. (j) Linear mRNA abundance per single cell according to UMAP analysis, of the four host mRNAs for signature circRNAs from (i). Visualized by colour coding, with blue indicating high expression.

4. Legend for supplementary tables

Suppl. Table S1

Differential expression of circRNAs in the EC lineage.

Expression levels of circRNAs of differentiated ECs (d6, d7, d10, d12) compared to the iPSC stage (d0) and mesoderm stage (d2, d3). Differential expression is given by $\log_2$ fold change ($\log_2$FC), *P* value and adjusted *P* value (adj. *P* value).

Suppl. Table S2

Differential expression of circRNAs in the SMC lineage.

Expression levels of circRNAs of differentiated SMCs (d6, d7, d10, d12) compared to the iPSC stage (d0) and mesoderm stage (d2, d3). Differential expression is given by $\log_2$ fold change ($\log_2$FC), *P* value and adjusted *P* value (adj. *P* value).

Suppl. Table S3

Differential circular-to-linear ratio in the EC lineage.

Circular-to-linear ratio of circRNAs of differentiated ECs (d6, d7, d10, d12) compared to the iPSC stage (d 0) and mesoderm stage (d2, d3). Differential CLR is given by $\log_2$ fold change ($\log_2$FC), *P* value and adjusted *P* value (adj. *P* value).

Suppl. Table S4

Differential circular-to-linear ratio in the SMC lineage.

Circular-to-linear ratio of circRNAs of differentiated SMCs (d6, d7, d10, d12) compared to the iPSC stage (d0) and mesoderm stage (d2, d3). Differential CLR is given by $\log_2$ fold change ($\log_2$FC), *P* value, and adjusted *P* value (adj. *P* value).

Suppl. Table S5

Description of iPSC lines used for *in vitro* differentiation.

Technical description of the three iPSC lines analysed with name of the human pluripotent stem cell registry (hPSCreg name), the type of source cells, information about the donors (sex, ethnicity and age), karyotype and known diseases.

Suppl. Table S6

Motifs of the spliceosome.

The ID, name, and consensus sequence of gene motifs enriched in TSS (+/- 2kb) of differentially regulated genes of the KEGG annotated spliceosome are given together with the corresponding E values, adj. *P* values and Fisher adj. *P* values computed by CentriMO. Analyses were performed separately for differentially upregulated (regulation: up) and downregulated (Regulation: down) splicing factors during differentiation from iPSC to EC.

Suppl. Table S7

Differential expression of circRNAs from RNAseq in quiescent vs. proliferative SMCs. (accompanying Suppl. Fig. S18a)

Expression levels of circRNAs in quiescent SMCs after serum starvation compared to expression in proliferative SMCs after serum re-stimulation (from analysis of published RNAseq dataset [6, 8]). Differential expression is given by $\log_2$ fold change ($\log_2$FC), *P* value, and adjusted *P* value (adj. *P* value).

Suppl. Table S8

Differential expression of cardiomyocyte-derived circRNAs.

Expression levels of circRNAs of differentiated cardiomyocytes (d14) compared to the iPSC stage (d0) (from analysis of published RNAseq dataset[7]). Differential expression is given by $\log_2$ fold change ($\log_2$FC), *P* value, and adjusted *P* value (adj. *P* value).

Suppl. Table S9

Differential expression of EC-derived circRNAs in human aortic tissue.

Expression levels of EC-associated circRNAs in atherosclerotic arteries compared to expression in healthy arteries. Differential expression is given by $\log_2$ fold change ($\log_2$FC), *P* value, and adjusted *P* value (adj. *P* value).

Suppl. Table S10

Differential expression of SMC-derived circRNAs in human aortic tissue.

Expression levels of SMC-associated circRNAs in atherosclerotic arteries compared to expression in healthy arteries. Differential expression is given by log_2 fold change (log_2FC), *P* value, and adjusted *P* value (adj. *P* value).

Suppl. Table S11

Cell type-specific enrichment of circRNAs in EC, SMC, macrophages, T cells and B cells from single-cell RNAseq analysis

(accompanying Suppl. Fig. S23)

Up-regulated circRNAs in different cell types of human carotid atherosclerotic plaque with log_2 fold change > 0 and adjusted $P<0.05$ are given with the log_2 fold change (log_2FC), the percentage of cells expressing the circRNA in the mentioned cell type (pct.1) compared to other cell types (pct.2), *P* value, and adjusted *P* value (adj. *P* value).

Suppl. Table S12

Differential expression of PBMC-derived circRNAs in atherosclerotic and non-atherosclerotic individuals.

Expression levels of circRNAs in PBMCs from patients with atherosclerosis compared to expression in PBMCs from individuals without atherosclerosis. Differential expression is given by log_2 fold change (log_2FC), *P* value, and adjusted *P* value (adj. *P* value).

Suppl. Table S13

Oligonucleotides.

Sequences and modifications of primer and probes are given.

Suppl. Table S14

Software.

The names of used software, R packages, and databases and their corresponding versions and releases are given.

1. Lipps C, Klein F, Wahlicht T, Seiffert V, Butueva M, Zauers J, Truschel T, Luckner M, Koster M, MacLeod R, Pezoldt J, Huhn J, Yuan Q, Muller PP, Kempf H, Zweigerdt R, Dittrich-Breiholz O, Pufe T, Beckmann R, Drescher W, Riancho J, Sanudo C, Korff T, Opalka B, Rebmann V, Gothert JR, Alves PM, Ott M, Schucht R, Hauser H, Wirth D, May T. Expansion of functional personalized cells with specific transgene combinations. *Nat Commun* 2018;**9**:994.
2. Mocci G, Sukhavasi K, Ord T, Bankier S, Singha P, Arasu UT, Agbabiaje OO, Makinen P, Ma L, Hodonsky CJ, Aherrahrou R, Muhl L, Liu J, Gustafsson S, Byandelger B, Wang Y, Koplev S, Lendahl U, Owens GK, Leeper NJ, Pasterkamp G, Vanlandewijck M, Michoel T, Ruusalepp A, Hao K, Yla-Herttuala S, Vali M, Jarve H, Mokry M, Civelek M, Miller CJ, Kovacic JC, Kaikkonen MU, Betsholtz C, Bjorkegren JLM. Single-Cell Gene-Regulatory Networks of Advanced Symptomatic Atherosclerosis. *Circ Res* 2024;**134**:1405-1423.
3. Butler A, Hoffman P, Smibert P, Papalexi E, Satija R. Integrating single-cell transcriptomic data across different conditions, technologies, and species. *Nat Biotechnol* 2018;**36**:411-420.
4. Franzen O, Gan LM, Bjorkegren JLM. PanglaoDB: a web server for exploration of mouse and human single-cell RNA sequencing data. *Database (Oxford)* 2019;**2019**.
5. Kuleshov MV, Jones MR, Rouillard AD, Fernandez NF, Duan Q, Wang Z, Koplev S, Jenkins SL, Jagodnik KM, Lachmann A, McDermott MG, Monteiro CD, Gundersen GW, Ma'ayan A. Enrichr: a comprehensive gene set enrichment analysis web server 2016 update. *Nucleic Acids Res* 2016;**44**:W90-97.
6. Aherrahrou R, Lue D, Civelek M. Genetic regulation of circular RNA expression in human aortic smooth muscle cells and vascular traits. *HGG Adv* 2023;**4**:100164.
7. Krane M, Dressen M, Santamaria G, My I, Schneider CM, Dorn T, Laue S, Mastantuono E, Berutti R, Rawat H, Gilsbach R, Schneider P, Lahm H, Schwarz S, Doppler SA, Paige S, Puluca N, Doll S, Neb I, Brade T, Zhang Z, Abou-Ajram C, Northoff B, Holdt LM, Sudhop S, Sahara M, Goedel A, Dendorfer A, Tjong FVY, Rijlaarsdam ME, Cleuziou J, Lang N, Kupatt C, Bezzina C, Lange R, Bowles NE, Mann M, Gelb BD, Crotti L, Hein L, Meitinger T, Wu S, Sinnecker D, Gruber PJ, Laugwitz KL, Moretti A. Sequential Defects in Cardiac Lineage Commitment and Maturation Cause Hypoplastic Left Heart Syndrome. *Circulation* 2021;**144**:1409-1428.
8. Aherrahrou R, Guo L, Nagraj VP, Aguhob A, Hinkle J, Chen L, Yuhl Soh J, Lue D, Alencar GF, Boltjes A, van der Laan SW, Farber E, Fuller D, Anane-Wae R, Akingbesote N, Manichaikul AW, Ma L, Kaikkonen MU, Bjorkegren JLM, Onengut-Gumuscu S, Pasterkamp G, Miller CL, Owens GK, Finn A, Navab M, Fogelman AM, Berliner JA, Civelek M. Genetic Regulation of Atherosclerosis-Relevant Phenotypes in Human Vascular Smooth Muscle Cells. *Circ Res* 2020;**127**:1552-1565.
